# Ultralong organic phosphorescence from isolated molecules with repulsive interactions for multifunctional applications

**DOI:** 10.1038/s41467-022-32029-1

**Published:** 2022-08-19

**Authors:** Xiaokang Yao, Huili Ma, Xiao Wang, He Wang, Qian Wang, Xin Zou, Zhicheng Song, Wenyong Jia, Yuxin Li, Yufeng Mao, Manjeet Singh, Wenpeng Ye, Jian Liang, Yanyun Zhang, Zhuang Liu, Yixiao He, Jingjie Li, Zixing Zhou, Zhu Zhao, Yuan Zhang, Guowei Niu, Chengzhu Yin, Shasha Zhang, Huifang Shi, Wei Huang, Zhongfu An

**Affiliations:** 1 Key Laboratory of Flexible Electronics & Institute of Advanced Materials, Nanjing Tech University, Nanjing, China; 2 Frontiers Science Center for Flexible Electronics, MIIT Key Laboratory of Flexible Electronics, Northwestern Polytechnical University, Xi’an, China; 3 State Key Laboratory of Organic Electronics and Information Displays & Institute of Advanced Materials (IAM), Nanjing University of Posts & Telecommunications, Nanjing, China

**Keywords:** Optical materials, Polymers, Organic molecules in materials science

## Abstract

Intermolecular interactions, including attractive and repulsive interactions, play a vital role in manipulating functionalization of the materials from micro to macro dimensions. Despite great success in generation of ultralong organic phosphorescence (UOP) by suppressing non-radiative transitions through attractive interactions recently, there is still no consideration of repulsive interactions on UOP. Herein, we proposed a feasible approach by introducing carboxyl groups into organic phosphors, enabling formation of the intense repulsive interactions between the isolated molecules and the matrix in rigid environment. Our experimental results show a phosphor with a record lifetime and quantum efficiency up to 3.16 s and 50.0% simultaneously in film under ambient conditions. Considering the multiple functions of the flexible films, the potential applications in anti-counterfeiting, afterglow display and visual frequency indicators were demonstrated. This finding not only outlines a fundamental principle to achieve bright organic phosphorescence in film, but also expands the potential applications of UOP materials.

## Abstract (web summary)

Despite great success in generation of ultralong organic phosphorescence (UOP) by suppressing non-radiative transitions through attractive interactions, consideration of repulsive interactions on UOP remains largely unexplored. Here, the authors introduce carboxyl groups into organic phosphors, enabling formation of the intense repulsive interactions between the isolated molecules and the matrix in rigid environment.

## Introduction

**Fig. 1:**
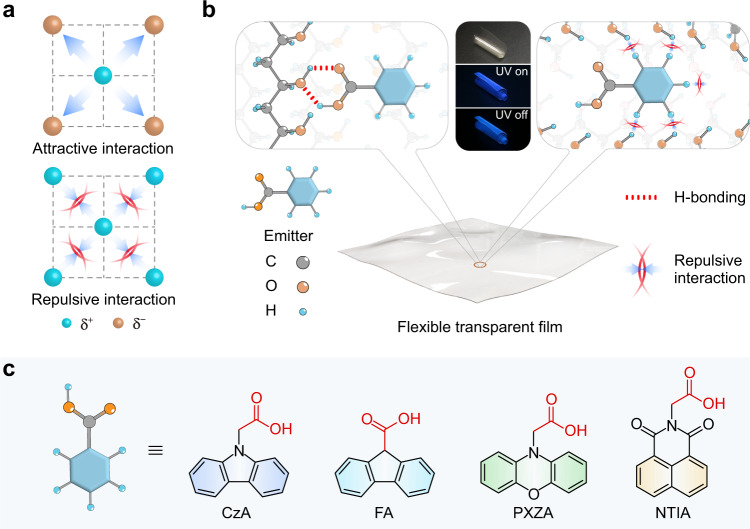
Schematic illustration of the isolated emitter doping into the film for UOP. **a** Demonstration of stabilizing molecules by attractive (top) and repulsive (bottom) interactions. **b** Intermolecular interactions between the emitters and polymer matrix in film. The schematic diagram of hydrogen bonding between the polymer chain and carboxylic group (left) as well as the repulsive interactions between the aromatic hydrogen of the emitter and the hydroxyl hydrogen in polymer (right). Inset: photographs of flexible transparent film with UOP taken under daylight (top), excitation by UV light (middle) and after the removal of the UV excitation (bottom), respectively. **c** Chemical structures of the emitters.

Ultralong organic phosphorescence (UOP), a type of persistent luminescence, has aroused scientific interest due to its long-lived luminescence lifetime and rich triplet state properties, which has been applied in various fields such as afterglow organic light-emitting diodes^1^, bio-imaging^2,3^, sensing^4,5^, anti-counterfeiting^6–8^ and so forth^9,10^. However, it is a challenge to obtain highly efficient UOP because of the weak spin-orbit coupling (SOC) and inevitable dissipation of the triplet excitons caused by molecular motions and quenchers in the surrounding environment, such as oxygen, moisture^11–13^. Recently, numerous efforts have been devoted to solve above issues following two ways. One is promoting the intersystem crossing (ISC) of excitons between singlet and triplet excited states by halogen atom^14–16^ and carbonyl groups^17–19^. The other is suppressing non-radiative transitions of excitons from excited triplet state to ground state by constructing a rigid molecular environment with crystal engineering^19–21^, self-assembly^22–25^, host-guest doping^26–34^, and polymerization^35,36^. Notably, abundant non-covalent interactions including hydrogen bonding^37–39^, electrostatic interaction^40–43^, etc. were used to restrict molecular motions. Therein, an attractive interaction plays a main role in the UOP generation (Fig. 1a top). A repulsive interaction, the counterpart of the attractive interaction, is also an important one to restrict the molecular motions in theory, which is seldom considered in the organic phosphorescence, however (Fig. 1a bottom).

Repulsive interactions widely exist in material science ranging from macroscopic matters to microscopic molecules^44–46^. For instance, a composite hydrogel with anisotropic mechanical properties developed, which is dominated by electrostatic repulsive force between negatively charged nanosheets embedded within it^46^. In macromolecules, the repulsive interactions play a vital role in stabilizing molecule conformations for functionalization, such as crystalline superstructures formed with soft clusters^47^, peptide folding between phosphorylated amino acids and tryptophan^48^, and the crystallization behavior of DNA-functionalized nanoparticles^49^. Despite great success in materials science, repulsive interactions have rarely been mentioned to improve phosphorescence properties of organic materials. It is worth noting that repulsion and attraction usually coexist in molecular systems^50^. Therefore, we reason that introduction of the carboxylic group and a short alkane may shorten the distance between the emitter and PVA polymer chain through hydrogen bonding, enabling the formation of the intense repulsive interactions between the aromatic hydrogens and the PVA polymer chains (Fig. 1b). Consequently, a rigid molecular environment was constructed to enhance phosphorescence at room temperature.

To validate our hypothesis, a series of carboxyl compounds, namely 2-(9*H*-carbazol-9-yl)acetic acid (CzA), 9*H*-fluorene-9-carboxylic acid (FA), 2-(10*H*-phenoxazin-10-yl)acetic acid (PXZA), and 2-(1,3-dioxo-1*H*-benzo[*de*]isoquinolin-2(3*H*)-yl)acetic acid (NTIA) (Fig. 1c) were designed and synthesized. Their chemical structures were thoroughly characterized by nuclear magnetic resonance spectroscopy (NMR), high-performance liquid chromatography (HPLC), high-resolution mass spectrometry (HRMS) and elemental analyses (Supplementary Figs. 1–28). As anticipated, after mixing the carboxyl molecules and polyvinyl alcohol (PVA) matrix, the films show colorful ultralong phosphorescence ranging from blue to orange after removal of the UV-light excitation under ambient conditions. Besides, as-prepared films also display self-healing capability, flexibility and tailorability, rendering the films with multifunctional applications.

## Results

### Photophysical properties for CzA doped film

**Fig. 2:**
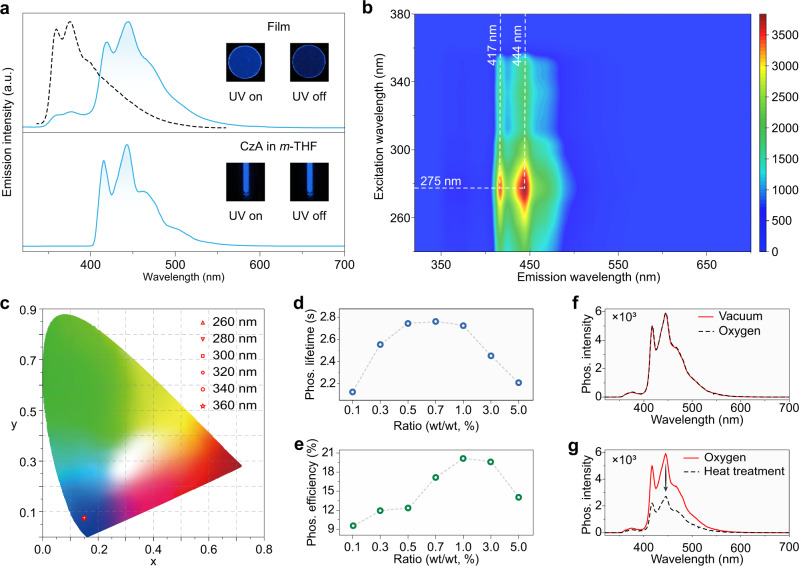
Photophysical properties of CzA in PVA film and dilute solution. **a** Normalized steady-state photoluminescence (dash line) and phosphorescence spectra (solid lines) of CzA in PVA film under ambient conditions (top) and in *m*-THF solution (1 × 10^−5 ^M) at 77 K (bottom). Inset: the left is photographs taken under 302 nm excitation, while the right is photographs of UOP after the removal of ultraviolet source. **b** Excitation-phosphorescence mapping of the CzA/PVA film under ambient conditions. **c** The CIE coordinates diagram for UOP of CzA/PVA film excited by UV light from 260 to 360 nm. The phosphorescence lifetimes **d** and efficiencies **e** of CzA/PVA films at various concentrations. **f** Phosphorescence spectra of CzA/PVA film (1.0 wt%) in vacuum and after exposed to oxygen for 30 min. **g** Phosphorescence spectra of CzA/PVA film (1.0 wt%) before and after heat treatment (333 K) in oxygen for 90 s.

Firstly, we selected CzA as a model emitter to investigate the photophysical properties of the CzA-doping PVA film (CzA/PVA). After removal of excitation with a 302 nm-light lamp, the CzA/PVA shows a remarkable blue afterglow, lasting for around 56 s by the naked eye (Supplementary Movie 1). As shown in Fig. 2a, the emission peaks of steady-state photoluminescence (PL) spectra for CzA/PVA locate at 357 and 373 nm, of which the lifetimes are in the nanosecond scale (Supplementary Fig. 29a and Supplementary Table 1). After a delay time of 8 ms, a structured spectrum emerges with main peaks at 417 and 444 nm. Impressively, the emission lifetimes were over 2.7 s. It is worth noting that the profile of the phosphorescence spectrum in film is consistent with that in dilute solution at 77 K, indicating the emitter CzA is isolated with the PVA matrix in single-molecule state (Supplementary Fig. 30). With the variation of the excitation wavelengths from 250 to 350 nm, there is no change to the profiles of the phosphorescence spectra (Fig. 2b). The corresponding Commission International de l’Eclairage (CIE) coordinates are at (0.15, 0.08), which are in the deep blue region (Fig. 2c).

In a further set of experiments, we investigated the influence of the ratio of CzA emitters doping into polymer matrix and atmospheres on phosphorescence performance at room temperature. With the concentration of CzA molecule increasing from 0.1 to 5.0 wt%, both phosphorescence lifetimes and efficiencies firstly increase and then decline in film (Fig. 2d, e, Supplementary Figs. 32 and 33 and Supplementary Table 3). Remarkably, the phosphorescence lifetime of CzA emitter reaches 2.76 s when the ratio of CzA is 0.7 wt.%. The highest phosphorescent quantum efficiency of CzA/PVA is 20.1%. Notably, the phosphorescence decay curves of films show multi-exponential decay characteristic, which was ascribed to the relaxation of the PVA polymer chains at room temperature^51,52^. Because we found that the phosphorescence decay curve of the CzA film becomes single exponential at 77 K (Supplementary Fig. 34 and Supplementary Table 4). From Fig. 2f, we found that phosphorescence intensity showed a negligible decrease for 30 min in oxygen atmosphere compared to that under vacuum at room temperature. After heating for 90 s, the phosphorescence intensity decreased about 55% owing to the permeation of oxygen (Fig. 2g), further indicating the phosphorescence nature of the afterglow emission. Additionally, under ambient conditions (298 K, HR: 41%), the phosphorescence intensity showed only a 5.6% decrease under continuous UV-light (300 nm) excitation for 30 min (Supplementary Fig. 35). However, when the humidity reaches 68%, the phosphorescence intensity of CzA/PVA films dramatically decreased to 50% within 4 min (Supplementary Fig. 36). However, there is no change in the profiles of phosphorescent spectra.

### Investigation for UOP mechanism of the molecules in film

**Fig. 3:**
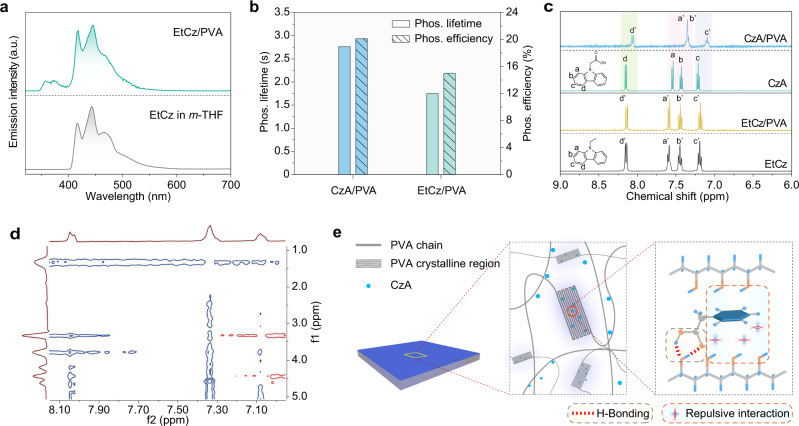
Mechanism investigation of ultralong phosphorescence in the emitter doped film. **a** Normalized phosphorescence spectra of EtCz in PVA film under ambient conditions (top) and in *m*-THF (1 × 10^−5 ^M) at 77 K (bottom). **b** Phosphorescence lifetimes and efficiencies of CzA and EtCz in PVA films. **c** ^1^H NMR spectra of the emitters within/without PVA matrix at 293 K. **d** Partial NOESY NMR spectrum of CzA/PVA in DMSO-*d*_*6*_ at 293 K. **e** A conceivable mechanism for UOP of the emitters in PVA film.

To gain deeper insight into high performance of phosphorescence for CzA/PVA, a set of control experiments on the change of the end groups and the alkane lengthen of the CzA molecule and matrix were conducted. As shown in Fig. 3a, b, the profiles of the phosphorescence spectra have little change, but the phosphorescence lifetimes and quantum efficiency exhibited a sharp fall. After concentration optimization, the control model of 9-ethyl-9*H*-carbazole (EtCz) without the carboxylic group showed an ultralong lifetime of 1.75 s and phosphorescence quantum efficiency of 15.0%. At 77 K, the profile of the phosphorescence spectrum for EtCz in dilute *m*-THF solution is consistent with CzA (Fig. 3a bottom), indicating there is no influence of the carboxylic group on triplet energy levels. Notably, the phosphorescence properties of CzA/PVA and EtCz/PVA based on synthesized carbazole are consistent with that by commercial carbazoles (Supplementary Fig. 41). For the model of 8-(9*H*-carbazol-9-yl)octanoic acid (CzOA) molecule, the phosphorescence lifetime becomes shorter than that of CzA (Supplementary Fig. 42). From PXRD analysis, it is found that there is no change of crystallinity for the PVA film after doping with CzA (Supplementary Fig. 43). The XRD diffraction peaks for the CzA/PVA and neat PVA films with specific values (19.59°) can be clearly identified. There is no change in the FT-IR spectra and the losing weight curves for the doped PVA films (Supplementary Figs. 44 and 45), indicating that the trace doping of the CzA molecules has little effect on the physical properties of the PVA matrix. Taken these results together, we speculated that the molecular interactions between CzA and PVA matrix were vital for the phosphorescence improvement of the isolated CzA molecules in PVA film.

Subsequently, we studied the supramolecular interactions between the emitters and polymer matrix by NMR spectrometer (Fig. 3c, d, Supplementary Figs. 46–55). In the aromatic region of CzA, there are four types of chemically equivalent hydrogen atoms. The signals for the aromatic protons shifts are at 7.55 (H_a_), 7.43 (H_b_), 7.21 (H_c_), and 8.16 ppm (H_d_), respectively. In the presence of PVA matrix, remarkably, the signals of such aromatic protons showed obviously upfield shifts of 0.16 (H_a_), 0.04 (H_b_), 0.09 (H_c_), and 0.07 ppm (H_d_), respectively (Fig. 3c and Supplementary Figs. 46–49), demonstrating the aromatic hydrogens of CzA were shielded with the hydroxyl hydrogen of PVA by the repulsive interaction^53–56^ (Fig. 3e). Inversely, there is no change of the signals of the aromatic protons in EtCz and CzOA molecules (Fig. 3c and Supplementary Fig. 53). Meanwhile, the correlation of the protons was further validated by a nuclear overhauser effect spectroscopy (NOESY) NMR spectrum of CzA/PVA (Fig. 3d). There indeed exist intense correlations between the hydroxyl of PVA and the H_a_ in CzA, as well as moderate correlations between the matrix and the H_b_, H_c_, and H_d_ in CzA, suggesting that aromatic hydrogens of CzA was efficiently restricted into the PVA matrix by repulsive interactions. In contrast, the ^1^H and NOESY NMR spectra of EtCz/PVA showed the absence of interaction between PVA and EtCz (Fig. 3c and Supplementary Fig. 55). Therefore, we concluded that the intense repulsive interactions between CzA and the PVA matrix play a critical role in phosphorescence enhancement.

Apart from the investigation of the influence of the end groups for the CzA molecule on phosphorescence, we further conducted a series of control experiments on different matrices, such as polyacrylic acid (PAA) and polyacrylamide (PAM). Like the molecules in PVA matrix, there is no change in the profiles of phosphorescence spectra for films with CzA and EtCz dopants (Supplementary Fig. 56a and b). For the phosphorescence lifetimes, the CzA-based films were longer, which was ascribed to the existence of the repulsive interaction between aromatic units and polymer matrices (Supplementary Fig. 56c–f and Supplementary Table 6). Notably, in comparison with the molecules in PAA matrix, there existed significant increase for phosphorescence lifetimes in the PAM matrix owing to intensive repulsive interactions (Supplementary Figs. 56e and 57). This finding further confirms the importance of the repulsive interactions for phosphorescence enhancement.

Given the above results, we proposed a conceivable mechanism for UOP of the CzA molecules in the PVA matrix, as shown in Fig. 3e. The introduction of the carboxylic group enables the formation of the intense repulsive interactions between carbazole units and the PVA matrix. After dispersed into the PVA matrix, the CzA molecules are isolated through repulsive interactions between carbazole groups and hydroxyl groups in PVA, further benefiting the suppression of non-radiative transitions. Thus, ultralong phosphorescence from the isolated molecules was obtained in CzA/PVA film under ambient conditions after photoexcitation (Supplementary Fig. 58).

### Extended experiments on the universality of the design principle

**Fig. 4:**
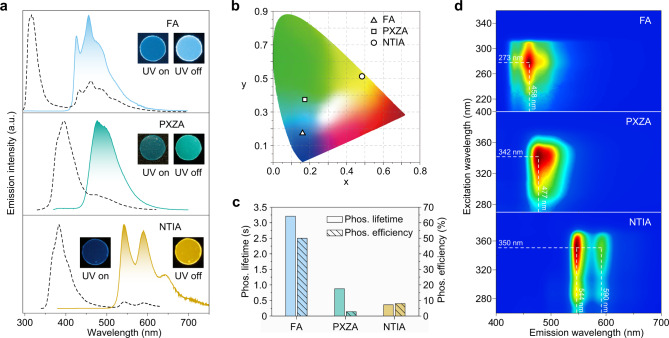
Photophysical properties of FA, PXZA and NTIA in PVA films under ambient conditions. **a** Normalized steady-state photoluminescence (dotted lines) and phosphorescence (solid lines) spectra of FA/PVA (top), PXZA/PVA (middle) and NTIA/PVA (bottle). Insets show the corresponding photographs of films taken before (left) and after (right) the ultraviolet source switching off. **b** The CIE coordinate diagram for the UOP colors of FA/PVA, PXZA/PVA and NTIA/PVA. **c** Phosphorescence lifetimes and efficiencies of FA/PVA (left), PXZA/PVA (middle) and NTIA/PVA (right). **d** Excitation-phosphorescence mapping of FA (top), PXZA (middle) and NTIA (bottom) in PVA film.

To prove the universality of our approach, a series of new molecules with carboxyl (FA, PXZA, and NTIA) were also mixed with the PVA polymers matrix, which were named FA/PVA, PXZA/PVA, and NTIA/PVA, respectively. Like CzA, we found that there also existed strong repulsive interactions between the aromatic hydrogen of the emitters and the hydroxyl hydrogen of the PVA matrix, which was verified by ^1^H NMR spectra (Supplementary Fig. 59a–c). As anticipated, all films showed intense afterglow emission after ceasing of the irradiation (Supplementary Movie 1). As shown in Fig. 4a, the emission peaks of FA/PVA, PXZA/PVA, and NTIA/PVA in steady-state PL spectra are at 305, 395, and 384 nm, of which the corresponding emission lifetimes are 4.9, 6.2, and 2.4 ns, respectively (Supplementary Fig. 29c–e). Specifically, FA/PVA and NTIA/PVA displayed structured phosphorescence spectra with maximum emission peaks at 458 and 544 nm, respectively, whereas PXZA/PVA showed broad phosphorescence emission with a main and at around 477 nm. It is worth noting that the profiles of the phosphorescence spectra in film are consistent with those in dilute solution at 77 K, indicating the emitters of FA, PXZA and NTIA are isolated with PVA matrix in single-molecule state (Supplementary Fig. 30c–e). Impressively, with conjugation variation of the molecules from FA, PXZA to NTIA, the afterglow emission of the films changed from sky blue, green to yellow, spanning a large emission color gamut (Fig. 4b). After optimization, FA/PVA, PXZA/PVA and NTIA/PVA showed the longest phosphorescence lifetimes of 3.21 s (0.1 wt%), 876.89 ms (1.0 wt%), and 362.61 ms (0.5 wt%) and the highest quantum efficiency of 50.0% (0.3 wt%), 2.8% (1.0 wt%), and 8.0% (0.7 wt%), respectively (Fig. 4c and Supplementary Figs. 67–72 and Supplementary Tables 7–9). To the best of our knowledge, it is the best phosphorescence material (FA/PVA at 0.3 wt%) considering both ultralong lifetimes and high quantum efficiency of phosphorescence (Supplementary Fig. 73). With the variation of the excitation wavelengths, there is no change for the profiles of the phosphorescence spectra (Fig. 4d).

### Applications of flexible UOP films

**Fig. 5:**
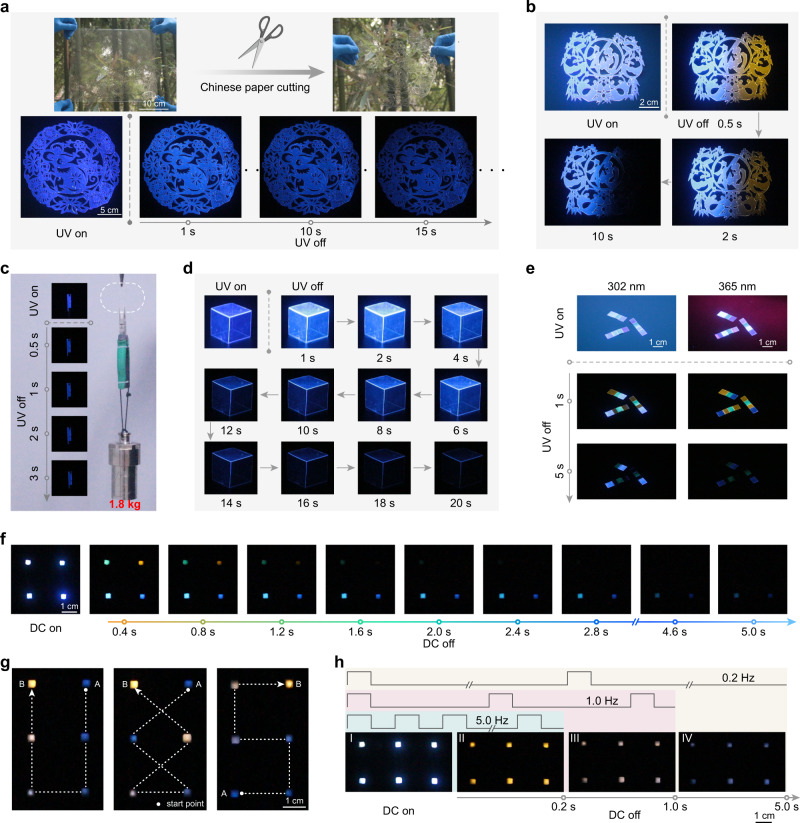
Description of UOP films for afterglow display, anti-counterfeiting and visual frequency detection under ambient conditions. **a**, **b** Transparent art crafts with UOP feature by Chinese paper-cutting technology based on the flexible films containing one or two emitters. Inset: photographs of the art crafts taken under sunlight and a 302 nm UV lamp as well as removal of the excitation, respectively. **c** Demonstration of self-healing property of the CzA/PVA film. Inset: photographs of the film after self-healing taken under a 302 nm UV lamp on/off. **d** Photographs of an emissive 3D cube (2 × 2 × 2 cm^3^) under a 302 nm lamp on/off. **e** Ribbons fabricated by self-heal under 302 nm (left) and 365 nm (right) lamps on and off. **f** A demo of afterglow display made of different UOP emitters under DC on/off. **g** Path tracing from A to B with colorful afterglow by controlling DC on and off. The tracks from blue to white then to yellow can be captured. **h** Demonstration of afterglow indicators by reversible cycle of afterglow colors. With frequency of electrical power change from 0.2 to 5.0 Hz, the afterglow cycles are I–II–III–IV, I–II–III and I–II, respectively.

Considering the feature of the long-lived afterglow of the emitters and the processability, flexibility and self-healing ability of PVA film, a set of potential applications were demonstrated. We firstly prepared pieces of transparent films based on different emitters in single or multiple components. With Chinese paper-cutting technology, a transparent pattern of art craft with the mouse was tailored, which showed intense blue afterglow (Fig. 5a and Supplementary Movie 2). Similarly, another Chinese paper-cut pattern based on two-component (CzA and NTIA) afterglow emitters showed a half of blue and a half of orange UOP after switching off the lamp (Fig. 5b). Regarding the standout self-healability of the PVA composite materials, an emissive 3D cube with blue afterglow was fabricated with CzA/PVA film (Fig. 5c, d and Supplementary Movie 3). Moreover, several ribbons with permutation and combination of PVA films doped with different afterglow molecules were prepared by utilization of self-healing ability. With variation of excitation (302 or 365 nm), the afterglow of ribbons varies with time (Fig. 5e), which is promising in potential applications of anti-counterfeiting and encryption.

Remarkably, the molecule doping PVA films were used as lighting panels for afterglow displays. As shown in Fig. 5f and Supplementary Movie 4, a demo of four electroluminescent devices was illustrated. After switching off the power source, the integrated devices displayed colorful afterglow with time. From Fig. 5g and Supplementary Movie 5, it is found that different tracks displayed with colorful afterglow from blue to white and then to yellow were clearly captured by the naked eye when the PVA film with mixed afterglow molecules of CzA and NTIA. Impressively, with frequency of electrical power change from 5.0, 1.0 to 0.2 Hz, the device showed reversible cycle of colorful afterglow (Fig. 5h, Supplementary Fig. 90 and Supplementary Movie 6), demonstrating the potential application for visual frequency indicators. For instance, the afterglow cycles between yellow and white (I–II–III) when the current frequency is 1.0 Hz.

## Discussion

In conclusion, we have reported a molecular design strategy to improve phosphorescence performance of the isolated molecules in film under ambient conditions. The introduction of the carboxylic group enables the formation of the intense repulsive interactions between the molecules and the PVA matrix, constructing a rigid molecular environment. Remarkably, the films display a phosphorescence lifetime of 3.16 s and a highly phosphorescence quantum efficiency up to 50% simultaneously. With tailoring the molecular structures of the emitters, the UOP colors of the films were regulated from deep blue to yellow. In view of self-healing capability, flexibility and tailorability, the films with UOP feature were applied into anti-counterfeiting, afterglow display and visual frequency indicators. These results not only provide a view to gain deeper insight into the phosphorescence of molecules in film, but also expand the scope of the potential applications of the UOP materials.

## Supplementary information

Supplementary Information

Description of Additional Supplementary Files

Supplementary Movie 1

Supplementary Movie 2

Supplementary Movie 3

Supplementary Movie 4

Supplementary Movie 5

Supplementary Movie 6

## Data Availability

All relevant data are included in this article and its Supplementary Information files. Source data are provided with this paper.

## Source data

Source Data
