# Supplementary material for: Ultralong organic phosphorescence from isolated molecules with repulsive interactions for multifunctional applications
Source: Nat Commun. 2022 Aug 19;13:4890. doi: 10.1038/s41467-022-32029-1 (PMC9391375; doi:10.1038/s41467-022-32029-1)
Supplement: Supplementary file 1 — Supplementary Information [file 41467_2022_32029_MOESM1_ESM.pdf]

Supplementary Information

Ultralong organic phosphorescence from isolated molecules with repulsive interactions for multifunctional applications

Xiaokang Yao[1], Huili Ma[1], Xiao Wang[1,2], He Wang[1], Qian Wang[1], Xin Zou[2], Zhicheng Song[1], Wenyong Jia[1], Yuxin Li[1], Yufeng Mao[1], Manjeet Singh[1], Wenpeng Ye[1], Jian Liang[1], Yanyun Zhang[1], Zhuang Liu[1], Yixiao He[1], Jingjie Li[1], Zixing Zhou[1], Zhu Zhao[1], Yuan Zhang[1], Guowei Niu[1], Chengzhu Yin[1], Shasha Zhang[1], Huifang Shi[1,3]*, Wei Huang[1,2,3]* & Zhongfu An[1]*

[1] Key Laboratory of Flexible Electronics & Institute of Advanced Materials, Nanjing Tech University, 30 South Puzhu Road, Nanjing 211816, P.R. China.
[2] Frontiers Science Center for Flexible Electronics, Xi'an Institute of Flexible Electronics and Xi'an Institute of Biomedical Materials & Engineering, Northwestern Polytechnical University, Xi'an, China.
[3] State Key Laboratory of Organic Electronics and Information Displays & Institute of Advanced Materials (IAM), Nanjing University of Posts & Telecommunications, 9 Wenyuan Road, Nanjing 210023, China.
** E-mail: iamhfshi@njtech.edu.cn; vc@nwpu.edu.cn; iamzfan@njtech.edu.cn*

Contents

I. Additional experimental details 2
II. Photophysical properties of organic emitters in film and solution 21
III. Understanding the relationship between ^{1}H NMR chemical shift and intermolecular interactions 33
IV. FT-IR, NMR, PXRD and TGA data for mechanism investigation 34
V. Expand experimental photophysical properties of emitters in film 48
VI. Applications 62
VII. Supplementary Movies 72
VIII. References 73

I. Additional experimental details

Reagents and materials

Unless other noted, all reagents used in the experiments were purchased from commercial sources without further purification.

Measurements

^{1}H and ^{13}C nuclear magnetic resonance (NMR) spectra were measured with a JOEL NMR spectrometer (JNM-ECZ400S, 400 MHz Japan). Chemical shift was relative to tetramethylsilane (TMS) as the internal standard. Elemental analysis was tested by a Vario EL Cube. High-performance liquid chromatography (HPLC) was performed using a SunFireTM C18 column conjugated to an ACQUITY UPLCH-class water HPLC system. High-resolution mass spectrometry (HRMS) was tested by an Agilent 6230 Series Accurate Mass TOF using ESI. Steady-state and delayed photoluminescence spectra were measured using Hitachi F-7000. The photoluminescence lifetimes and time-resolved emission spectra were collected on an Edinburgh FLSP 980 fluorescence spectrophotometer equipped with a xenon arc lamp (Xe900), a nanosecond hydrogen flash-lamp (nF920), and a microsecond flash-lamp (μF900), respectively. Photoluminescence efficiency was obtained on a Hamamatsu Absolute PL Quantum Yield Spectrometer C11347. FT-IR spectra were collected by using a Thermo Scientific Nicolet iS50 with a reflection accessory. Thermogravimetric analysis (TGA) was performed using Mettler Toledo TGA 2. Powder X-ray diffraction (PXRD) patterns were collected on Smartlab (3 kW) X-ray diffractometer of Japanese brand. Scanning electron microscopy (SEM) images were obtained by a (JSM-7800F) scanning electron microscope. Photographs and videos of steady-state photoluminescence and afterglow were taken by a Cannon EOS 700D camera.

Test environment

Unless other noted, all photophysical properties of the films were collected at 298 K with relative humidity (RH) of ~30% in air.

Film preparation

Firstly, two solutions were prepared. One is ethanol solution of organic phosphors with a concentration of 10 mg/mL. The other one is homogeneous aqueous solution of polymers, which was prepared with PVA-1799 (100 mg) and distilled water (1.0 mL) at 373 K for 6 h. Taking 1.0 wt.% as an example, the phosphor solution (100 μL) was added into the PVA-1799 aqueous solution, and mixed at 373 K for 6 h. Then, the mixed solution (300 μL) was dropped onto a 1.5×1.5 cm^2 quartz wafer, which was dried at 333 K for 12 h. Finally, a transparent film was obtained.

Fabrication of the large-area transparent film for CzA: Firstly, a mixture of PVA-1799 (20.0 g), CzA (0.2 g) and ethanol (20 mL) dissolved in distilled water (200 mL), which was stirred at 373 K for 6h. Then, the mixed solution was dried on a glass plate at 333 K for 12 h. After exfoliation, a lager-area transparent film was obtained.

Preparation of the lager-area transparent films with two UOP colors: Following the film forming process of CzA, the mixed aqueous solution (100 mL) of ethanol (10 mL), NTIA (0.07 g) and PVA-1799 (10 g) was firstly prepared. A clapboard was installed in the middle of glass plate. Then the mixed solution with CzA or NTIA was added in the corresponding region on glass plate, respectively. After that, the clapboard was removed. Two parts stitched together by the liquidity of liquid. After drying at 333 K for 12 h, a transparent film with two UOP colors was obtained.

Preparation for ^1H NMR measurement: The prepared film (20 mg) was firstly dispersed in DMSO-d_6 (0.5 mL). Then the NMR spectra were collected after the mixture was placed in the dark environment for 6 h.

General procedures for synthesis of phosphorescence emitters

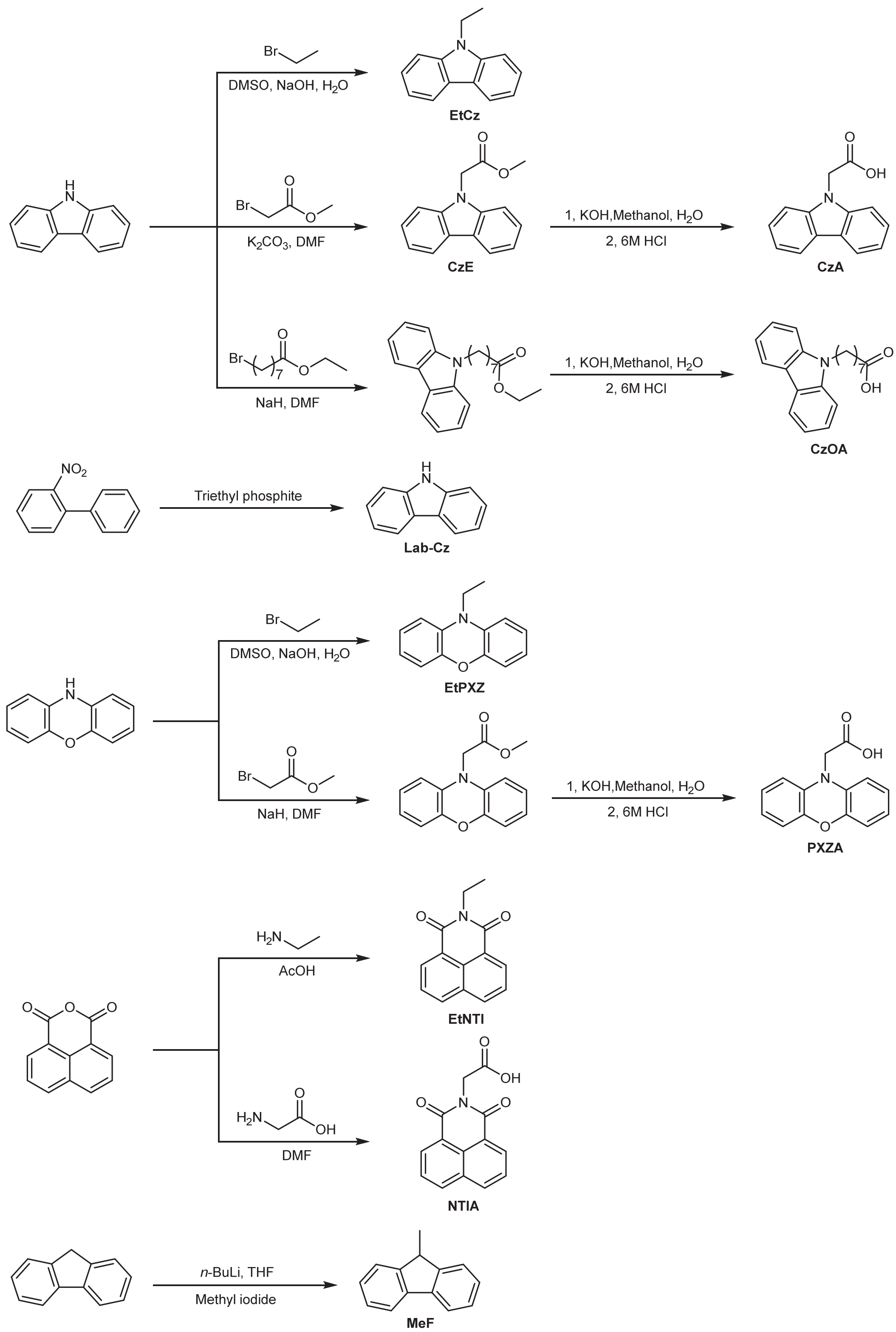

Supplementary Fig. 1 | Synthetic routes of the target molecules. The details for the synthetic routes of phosphors EtCz, CzA, CzOA, Lab-Cz, EtPXZ, PXZA, EtNTI, NTIA and MeF were listed.

9-Ethyl-9*H*-carbazole (EtCz). To a mixed solution of 50% aqueous NaOH (4 mL), DMSO (12 mL) and carbazole (1.0 g, 6 mmol) was slowly added 1-bromoethane (0.72 g, 6.6 mmol) at room temperature. The mixture solution

was stirred 12 h at 363 K and then cooled to room temperature. After that, the brine was added to the mixture and the organic phase extracted with dichloromethane was dried over $MgSO_4$. The solvent was removed and the residue was purified by a column chromatography (Ethyl acetate/petroleum ether, *v*/*v*, 1/2) to afford white solid (1.16 g, 5.95 mmol, yield: 90%). ^{1}H NMR (400 MHz, DMSO-*d*$_6$): δ 1.30 (t, *J* = 6.9 Hz, 3H), 4.44 (q, *J* = 7.0 Hz, 2H), 7.19 (t, *J* = 7.4 Hz, 2H,), 7.45 (t, *J* = 7.6 Hz, 2H), 7.60 (d, *J* = 8.1 Hz, 2H), 8.15 (d, *J* = 7.7 Hz, 2H). ^{13}C NMR (101 MHz, DMSO-*d*$_6$): δ 140.01, 126.20, 122.65, 120.85, 119.17, 109.56, 37.41, 14.20. Analysis (calcd., found for $C_{14}H_{13}N$): C (86.12, 86.14), H(6.71, 6.82), N(7.17, 7.04).

9-Ethyl-9*H*-carbazole (CzA). To a 25 mL flask containing 9*H*-carbazol (1.0 g, 6 mmol), potassium carbonate (1.24 g, 9.0 mmol) and dry DMF (7 mL) were added. The reaction mixture was stirred at 328 K for 2 h. Then methyl bromoacetate (1.7 mL, 18 mmol) was added to the flask and the reaction mixture was stirred at 328 K for 6 h. Then, the mixture was poured into water and a white solid precipitate was obtained. The compound was filtered and used for the next reaction without further purification. A mixture of methyl 2-(9*H*-carbazol-9-yl)acetate (**CzE**) (1.40 g, 5.8 mmol) and KOH (0.70 g, 12.6 mmol) was reacted for 7 h in the solvent of MeOH/H_2O (20 mL/20 mL) at 373 K. After that, the pH of the solution after filtering was adjusted to 6-7 with 6 M HCl aqueous solution. A white solid (1.30 g, 5.77 mmol) was obtained with a yield of 96%. Finally, the crude product was recrystallized with ethyl acetate to yield colorless transparent crystal (1.0 g). ^{1}H NMR (400 MHz, DMSO-*d*$_6$): δ 5.19 (s, 2H), 7.18 (m, *J* = 7.5 Hz, 2H), 7.40 (m, *J* = 7.3 Hz, 2H), 7.51 (d, *J* = 8.0 Hz, 2H), 8.12 (d, *J* = 7.8 Hz, 2H), 12.98 (s, 1H). ^{13}C NMR (101 MHz, DMSO-*d*$_6$): δ 170.82, 140.98, 126.25, 122.76, 120.71, 119.63, 109.80, 44.47. Analysis (calcd., found for $C_{14}H_{11}NO_2$): C (74.65, 74.65), H (4.92, 4.87), N (6.22, 6.14). HRMS: $(CzA+Na)^+$ m/z: 248.0683 (theoretical value: 248.0687).

8-(9H-carbazol-9-yl)octanoic acid (CzOA). To a 25 mL flask containing carbazole (1.0 g, 6.0 mmol), NaH (0.48 g, 12 mmol, 60% wt./wt. dispersion in mineral oil) and dry DMF (7 mL) were added. The reaction mixture was stirred at 323 K for 2 h. Subsequently, ethyl 8-bromooctanoate (3.0 g, 12 mmol) was added to the flask and the reaction mixture was stirred at 323 K for another 10 h. After that, the mixture was poured into water and extracted with ethyl acetate. The organic phase was dried over anhydrous Na_2SO_4, filtered and concentrated *in vacuo*. The oily compound (1.8 g, 5.34 mmol) and KOH (0.70 g, 12.6 mmol) were reacted for 7 h in MeOH/H_2O (20 mL/20 mL) at 373 K. After the reaction completed, the solution after filtration was treated with 6 M HCl aqueous solution to adjust its pH to 6-7. The resultant solution was extracted with ethyl acetate and purified by the column chromatography to afford oily compound (1.4 g, 4.53 mmol). The yield is 77.8%. ^{1}H NMR (400 MHz, DMSO-*d*$_6$): δ 1.23 (m, 8H), 1.43 (m, 2H), 1.75 (m, 2H), 2.15 (t, *J* = 7.5 Hz, 2H), 4.38 (t, *J* = 7.1 Hz, 2H), 7.19 (t, *J* = 7.4 Hz, 2H) 7.44 (t, *J* = 7.7 Hz, 2H), 7.58 (d, *J* = 8.2 Hz, 2H), 8.14 (d, *J* = 7.7 Hz, 2H) 11.96 (s, 1H). ^{13}C NMR (101 MHz, DMSO-*D*$_6$): δ 24.45, 26.42, 28.50, 28.53, 33.63, 42.18, 109.25, 118.64, 120.29, 122.04, 125.69, 140.00, 174.56.

Carbazole (lab-Cz). A mixture of triethyl phosphite (3.32g, 20 mmol) and 2-nitrodiphenyl (1.99g ,10 mmol) was

refluxed under N_2 atmosphere for 24 h. The triethyl phosphite and triethyl phosphate were moved by reduced pressure distillation, and the residue was purified by the column chromatography to afford white flake crystal (1.34 g, 8.02 mmol, 80%). The product was further purified by recrystallization in acetone. ^{1}H NMR (400 MHz, DMSO-d_6): δ 7.15 (t, *J* = 7.5 Hz, 2H), 7.38 (t, *J* = 8.1 Hz 2H), 7.49 (d, *J* = 8.1 Hz, 2H), 8.10 (d, *J* = 8.3 Hz, 2H), 11.25 (s, 1H). ^{13}C NMR (101 MHz, DMSO-d_6): δ 140.23, 126.03, 122.90, 120.68, 119.00, 111.45.

Lab-CzA and **Lab-EtCz** were synthesized from lab-Cz with the same methods of **CzA** and **EtCz**.

2-(10*H*-Phenoxazin-10-yl)acetic acid (PXZA). To a 25 mL flask containing phenoxazine (1.0 g, 5.5 mmol), NaH (0.48 g, 12 mmol, 60% wt./wt. dispersion in mineral oil) and dry DMF (7 mL) were added. The reaction mixture was stirred at 298 and 323 K for 2 h each. Then methyl bromoacetate (1.2 mL, 13 mmol) was added to the flask and the reaction mixture was stirred at 323 K for 10 h. After that, the precipitate was obtained after the mixture reaction solution was poured into water. The compound was filtered and used for the next reaction without further purification. A mixture of methyl 2-(10*H*-phenoxazin-10-yl)acetate (1.33 g, 5.2 mmol) and KOH (0.70 g, 12.6 mmol) was reacted for 7 h in MeOH/H_2O (20 mL/20 mL) at 373 K. Then the pH of the solution after filtering was adjusted to 6-7 with 6 M HCl aqueous solution. A white solid (1.0 g, 4.15 mmol) was obtained with a yield of 75%. Finally, the crude product was recrystallized with ethyl acetate to yield colorless transparent crystal. ^{1}H NMR (400 MHz, DMSO-d_6): δ 4.38 (s, 2H), 6.56 (d, *J* = 7.7 Hz, 2H), 6.71 (m, 4H), 6.83 (m, 2H), 13.01 (s, 1H). ^{13}C NMR (101 MHz, DMSO-d_6): δ 170.64, 144.33, 133.50, 124.17, 121.53, 115.28, 112.41, 45.26. Analysis (calcd., found for $C_{14}H_{13}NO$): C (75.59, 75.62), H (6.20, 6.30), N (6.63, 6.65). HRMS: $(PXZA+Na)^+$ m/z: 264.0631 (theoretical value: 264.0636).

10-ethyl-10*H*-phenoxazine (EtPXZ). To a mixed solution of 50% aqueous NaOH (4 mL), DMSO (12 mL) and phenoxazine (1.0 g, 5.5 mmol) was slowly added 1-bromoethane (0.66 g, 5.5 mmol) at room temperature. The mixture was stirred at 363 K for 12 h and then cooled to room temperature. After that, the brine was added into the mixture. The organic phase was extracted with dichloromethane, which was further dried with $MgSO_4$. After the solvent was removed, the residue was purified by a column chromatography (ethyl acetate/petroleum ether, *v*/*v*, 1/2) to afford white solid (1.0 g, 4.73 mmol) with a yield of 86%. ^{1}H NMR (400 MHz, DMSO-d_6): δ 1.11 (t, *J* = 7.0 Hz, 3H), δ 3.63 (q, *J* = 7.0 Hz, 2H), δ 6.64 (d, *J* = 4.0 Hz, 4H), δ 6.68 (m, *J* = 7.8 Hz, 2H), δ 6.82 (m, 2H). ^{13}C NMR (101 MHz, DMSO-d_6): δ 163.62, 134.74, 131.72, 131.11, 127.73, 127.65, 122.49, 35.26, 13.64. Analysis (calcd., found for $C_{14}H_{11}NO_3$): C (69.70, 69.70), H (4.60, 4.28), N (5.81, 5.48). HRMS: $(EtPXZ+H)^+$ m/z: 211.0993 (theoretical value: 211.1075).

2-(1,3-Dioxo-1*H*-benzo[*de*]isoquinolin-2(3H)-yl)acetic acid (NTIA). Benzo[*de*]isochromene-1,3-dione (0.99 g, 5 mmol) and glycine (0.38g, 5 mmol) were added into a three-neck flask with 10 mL DMF. The mixture reacted for 8 h at 363 K. After that, the mixture was filtrated to get the pale yellow powder. After washed with ethanol till the filter

liquor turned colorless, the powder was purified by a column chromatography (dichloromethane/methyl alcohol, *v*/*v*, 10/1) to obtain white power (0.90 g, 3.52 mmol) with a yield of 70%. ^{1}H NMR (400 MHz, DMSO-d_6): δ 4.74 (s, 2H), 7.89 (m, 2H), 8.50 (m, 4H), 13.11 (s, 1H). ^{13}C NMR (101 MHz, DMSO-d_6): δ 169.89, 163.55, 135.31, 131.80, 131.57, 127.81, 127.78, 121.89, 41.66. Analysis (calcd., found for $C_{14}H_9NO_4$): C (65.88, 65.85), H (3.55, 3.50), N (5.49, 5.35). HRMS: $(NTIA+Na)^+$ m/z: 278.0423 (theoretical value: 278.0429).

2-ethyl-1*H*-benzo[*de*]isoquinoline-1,3(2*H*)-dione (EtNTI). 1,8-Naphthalene anhydride (1.00 g, 5.05 mmol) and ethylamine (2.0 mol/L in THF, 3.00 mL, 6 mmol) were added to a round-bottomed flask containing 20 mL acetic acid. The solution was stirred at 120°C overnight. Then the mixture was poured into ice water. The resulting precipitate was collected by centrifugation, which was washed with ether and purified on a silica gel column (petroleum ether/EtOAc, v/v, 1/1) to give the final product (0.98 g, 4.34 mmol, 87% yield) with a yield of 87%. ^{1}H NMR (400 MHz, DMSO-d_6): δ 1.19 (t, *J* = 6.8 Hz, 3H), δ 4.03 (q, *J* = 7.1 Hz, 2H), δ 7.79 (t, *J* = 7.8 Hz, 2H), δ 8.38 (q, 4H). ^{13}C NMR (101 MHz, DMSO-d_6): δ 144.67, 133.11, 124.65, 121.23, 115.54, 112.31, 37.91, 10.24. Analysis (calcd., found for $C_{14}H_{11}NO_2$): C (74.65, 74.69) H (4.92, 4.88) N (6.22, 6.18).

9*H*-fluorene-9-carboxylic acid (FA). 9*H*-fluorene-9-carboxylic acid (5 g, 23.8 mmol) was added to a mixed solution with 50 mL methanol and 50 mL water, in which KOH (2 g, 35.71 mmol) was dissolved, and hydrolyzed at 373 K for 6 h. Subsequently, activated carbon (~ 2 g) was added to the mixed solution, which was further stirred at 373 K for 30 minutes. After the filtrate was cooled to room temperature, 6 M HCl aqueous solution was added drop by drop to form white solid precipitation. Then, the white precipitate was filtrated and vacuum dried. Colorless transparent crystals were obtained by recrystallization with dichloromethane three times. ^{1}H NMR (400 MHz, DMSO-d_6): δ 4.97 (s, 1H), 7.35 (m, 2H), 7.43 (m, 2H), 7.65 (m, 2H), 7.89 (d, *J* = 7.5 Hz, 2H), 12.84 (s, 1H). ^{13}C NMR (101 MHz, DMSO-d_6): δ 172.35, 141.83, 141.37, 128.38, 127.81, 126.18, 120.62, 53.81. Analysis (calcd., found for $C_{14}H_{10}O_2$): C (79.98, 79.71) H (4.79, 4.51).

9-methyl-9*H*-fluorene (MeF). *n*-BuLi (1.6 mol/L in hexane, 5.28 mL, 8.44 mmol) was firstly added to a solution of fluorene (1.08 g, 6.49 mmol) in THF (25 mL) under a N_2 atmosphere at -78°C. Then methyl iodide (0.61 mL, 9.74 mmol) in THF (5 mL) was added dropwise within 10 min. After that, the mixture was allowed to come to room temperature for 3 h, which was further quenched with saturated aqueous NH_4Cl (10 mL). The aqueous phase was extracted with dichloromethane (15 mL) for three times. The combined organic layers were dried (Na_2SO_4), concentrated, and purified by column chromatography (petroleum ether/EtOAc, v/v, 20/1) to afford target white solid (1.11 g, 6.16 mmol) with a yield of 95%. It was further purified by vacuum distillation. ^{1}H NMR (400 MHz, DMSO-d_6): δ 1.45 (d, *J* = 6.3 Hz, 3H) δ 3.93 (t, *J* = 6.8 Hz, 1H), 7.34 (m, 4H), 7.57 (d, *J* = 7.5 Hz), 7.85 (d, *J* = 6.3 Hz, 2H). ^{13}C NMR (101 MHz, DMSO-d_6) δ 149.15, 140.42, 127.57, 127.50, 124.69, 120.48, 42.37, 18.64. Analysis (calcd., found for $C_{14}H_{12}$): C (93.29, 93.30) H (6.71; 6.70).

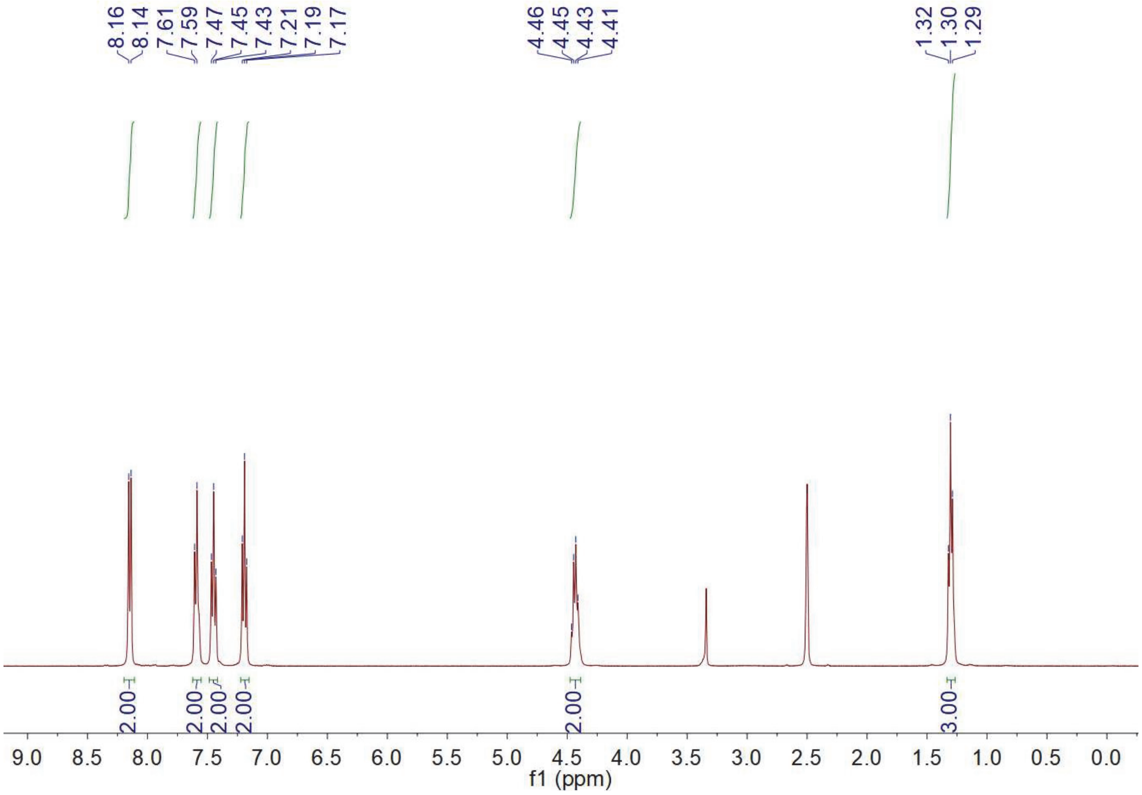

Supplementary Fig. 2 | ^{1}H NMR spectrum. The spectrum of EtCz molecule was collected in DMSO-d_6 under ambient conditions.

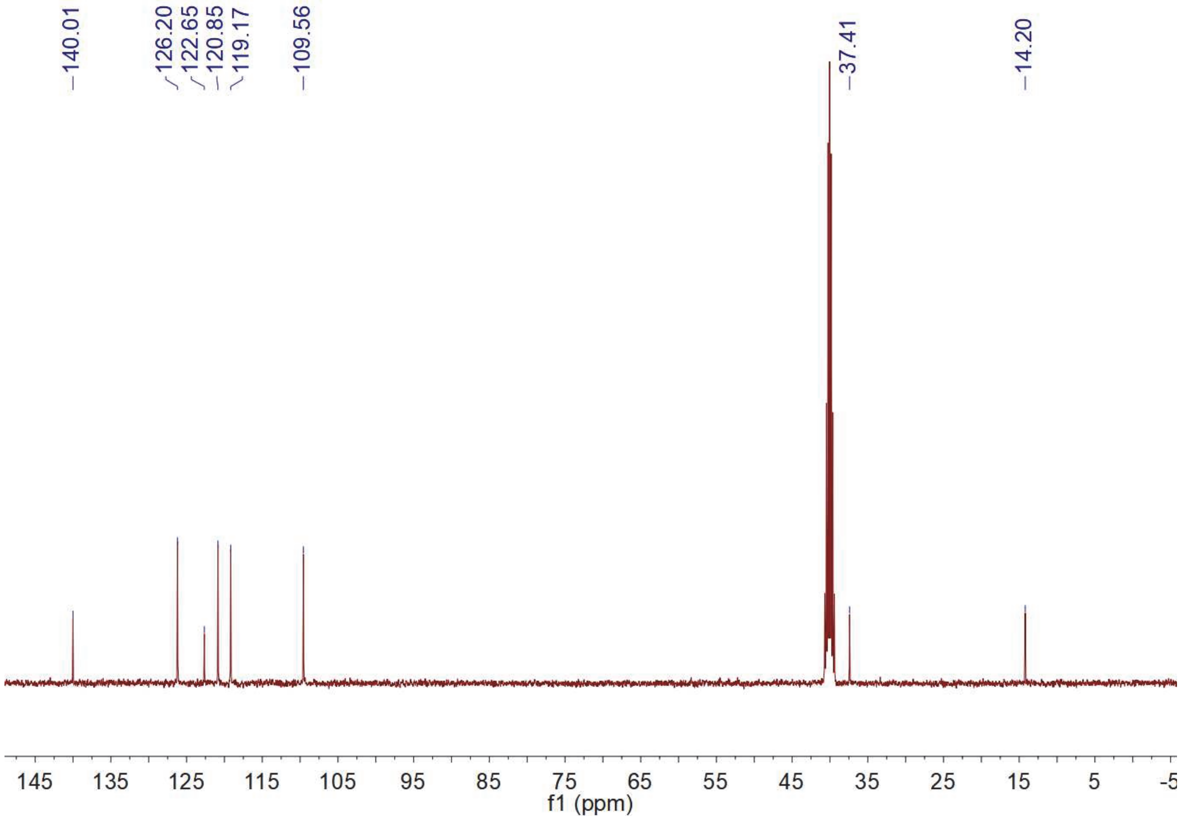

Supplementary Fig. 3 | ^{13}C NMR spectrum. The spectrum of EtCz molecule was collected in DMSO-d_6 under ambient conditions.

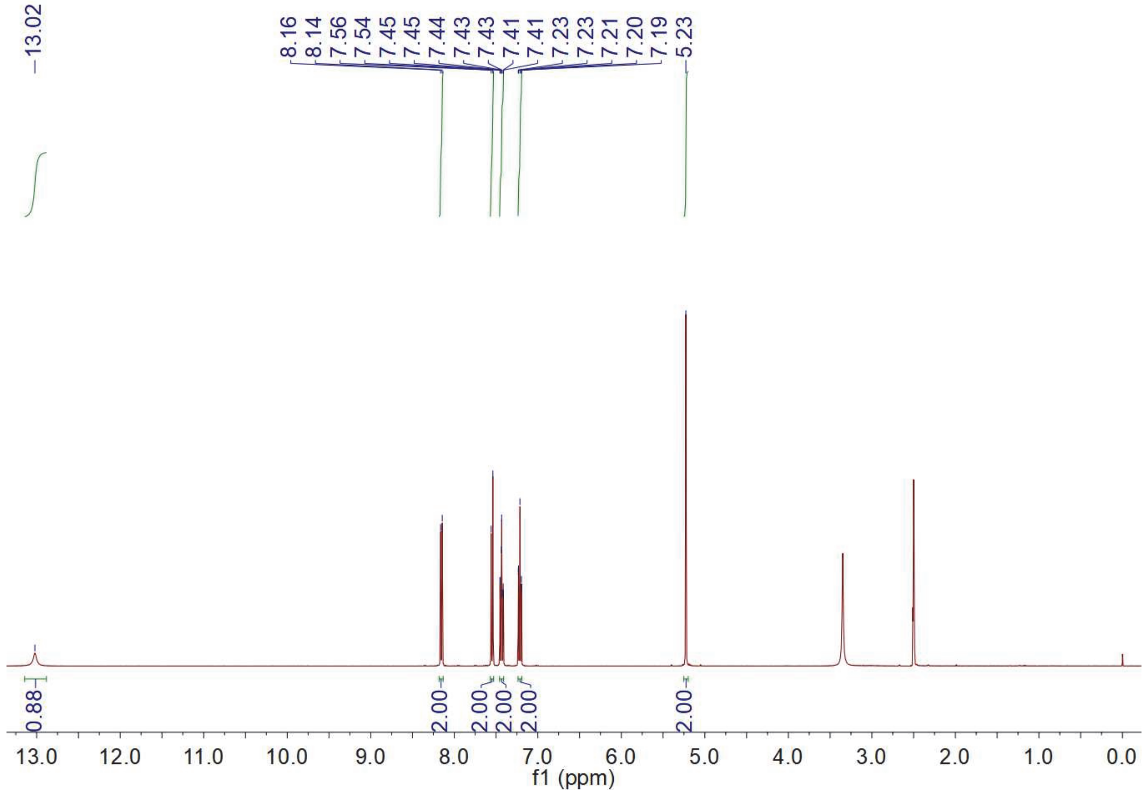

Supplementary Fig. 4 | ^{1}H NMR spectrum. The spectrum of CzA molecule was collected in DMSO-d_6 under ambient conditions.

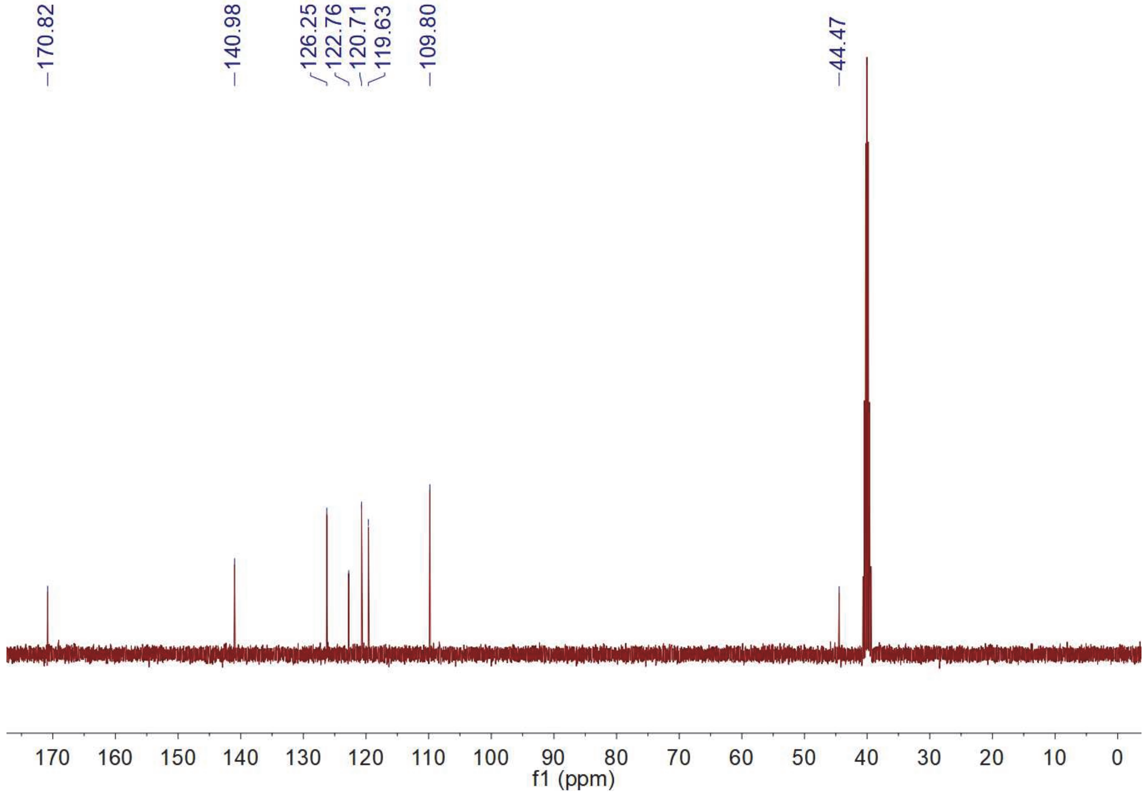

Supplementary Fig. 5 | ^{13}C NMR spectrum. The spectrum of CzA molecule was collected in DMSO-d_6 under ambient conditions.

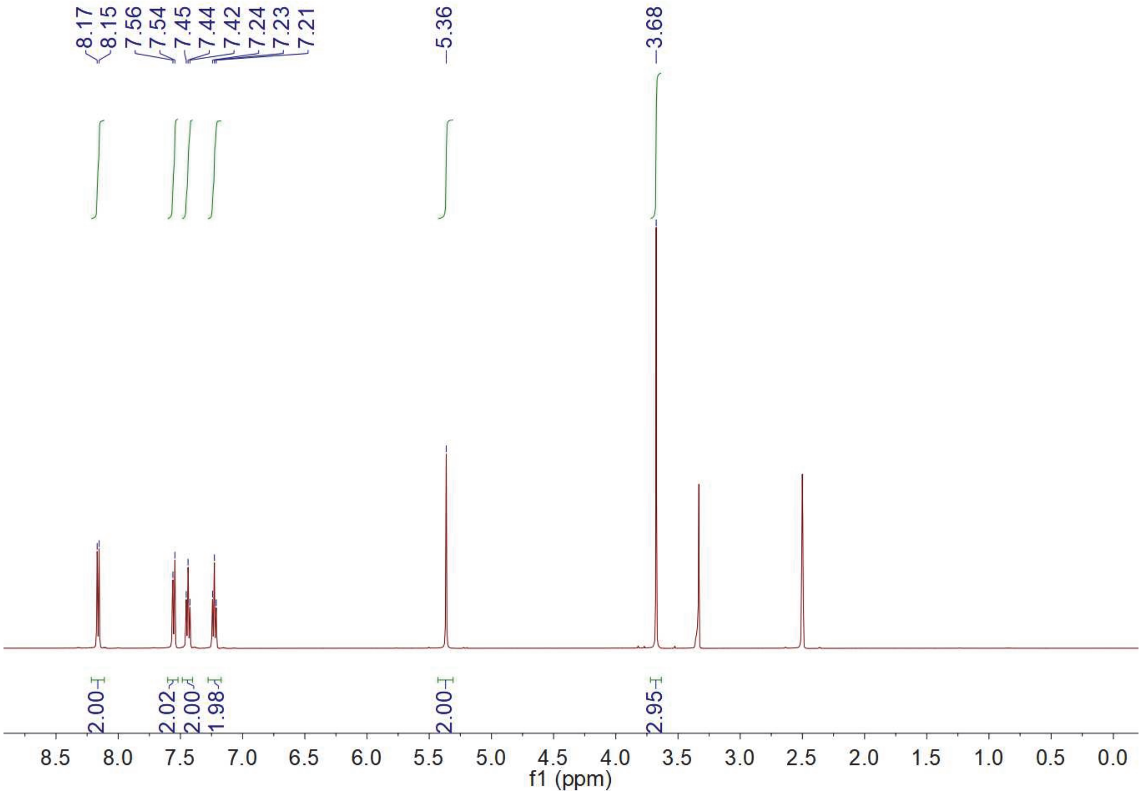

Supplementary Fig. 6 | ^{1}H NMR spectrum. The spectrum of CzE molecule was collected in DMSO-d_6 under ambient conditions.

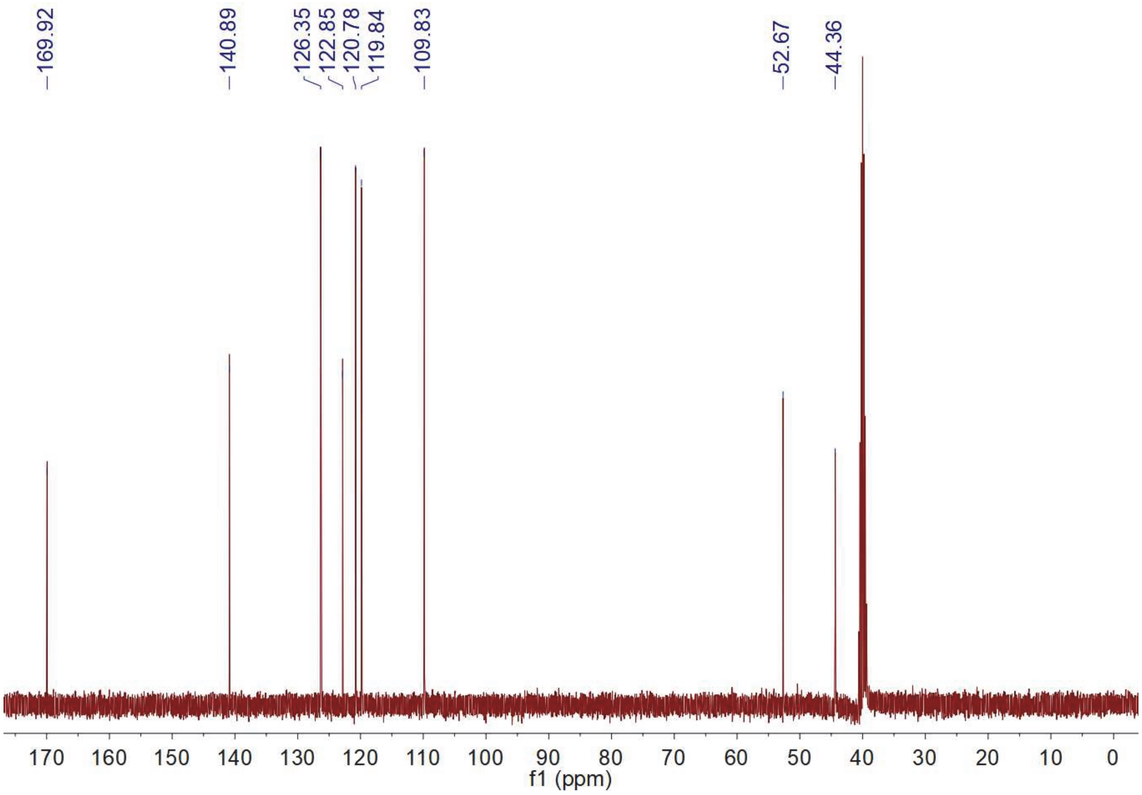

Supplementary Fig. 7 | ^{13}C NMR spectrum. The spectrum of CzE molecule was collected in DMSO-d_6 under ambient conditions.

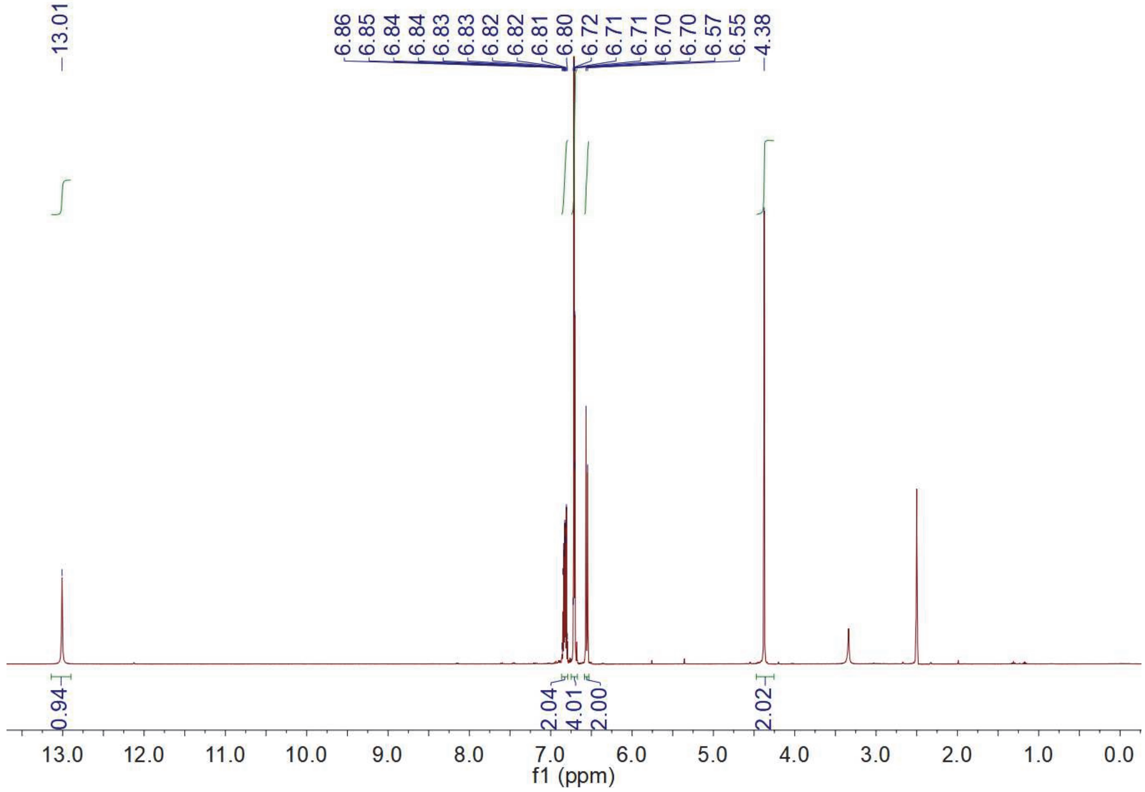

Supplementary Fig. 8 | ^{1}H NMR spectrum. The spectrum of PXZA molecule was collected in DMSO-d_6 under ambient conditions.

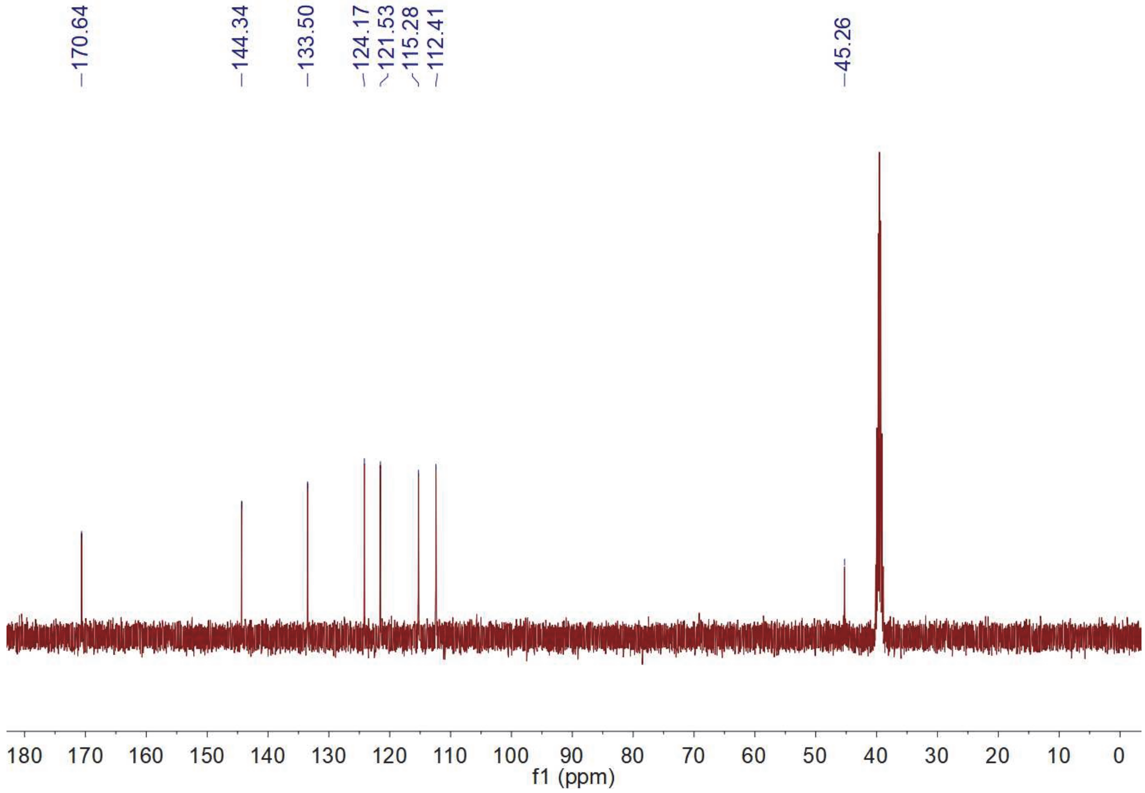

Supplementary Fig. 9 | ^{13}C NMR spectrum. The spectrum of PXZA molecule was collected in DMSO-d_6 under ambient conditions.

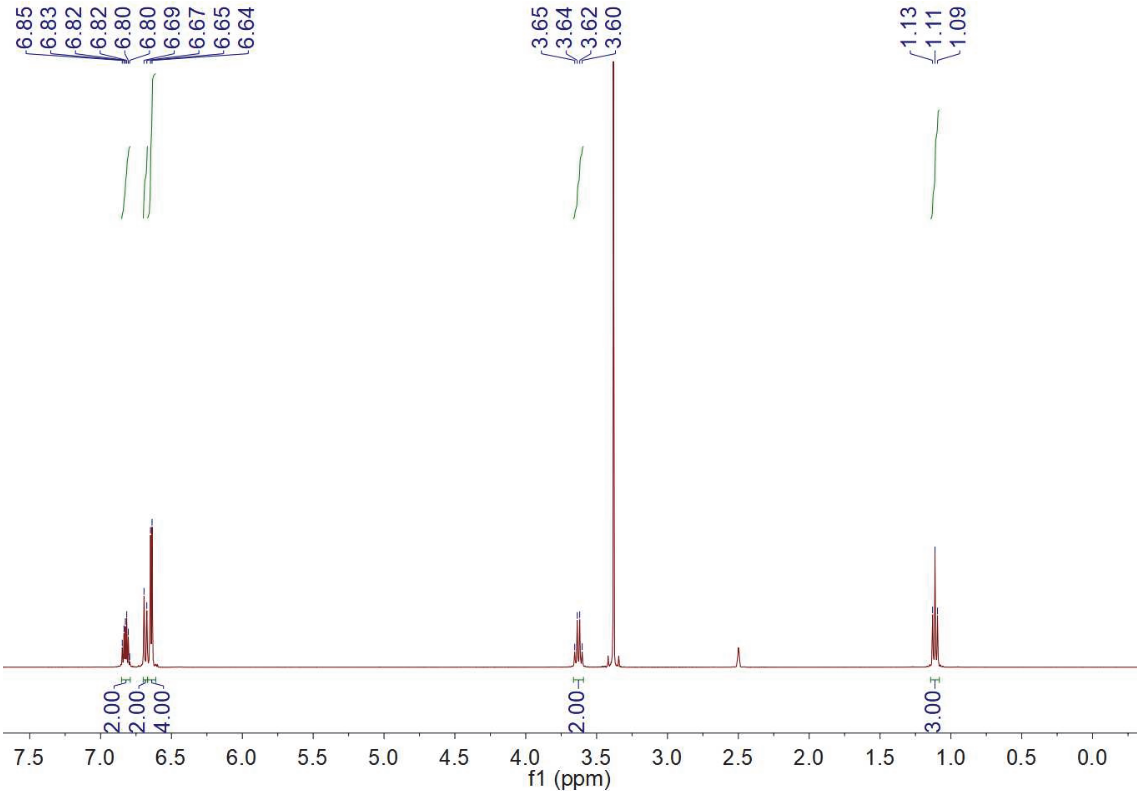

Supplementary Fig. 10 | ^{1}H NMR spectrum. The spectrum of EtPXZ molecule was collected in DMSO-d_6 under ambient conditions.

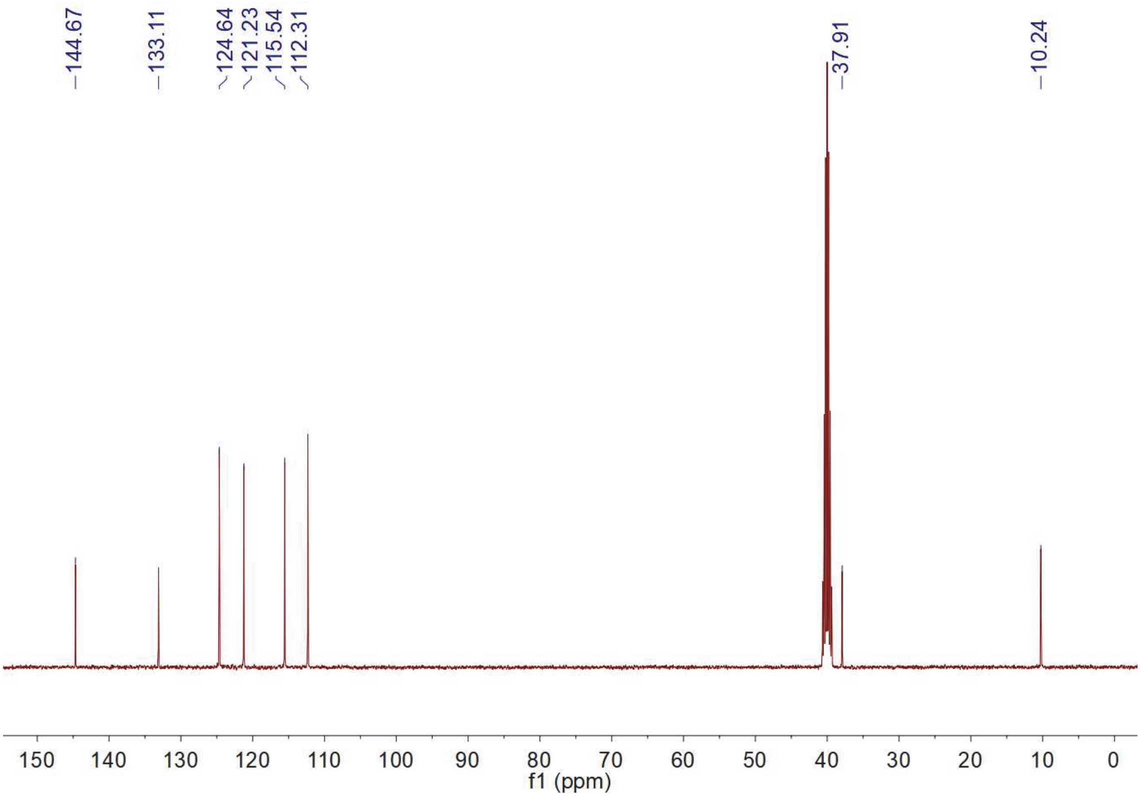

Supplementary Fig. 11 | ^{13}C NMR spectrum. The spectrum of EtPXZ molecule was collected in DMSO-d_6 under ambient conditions.

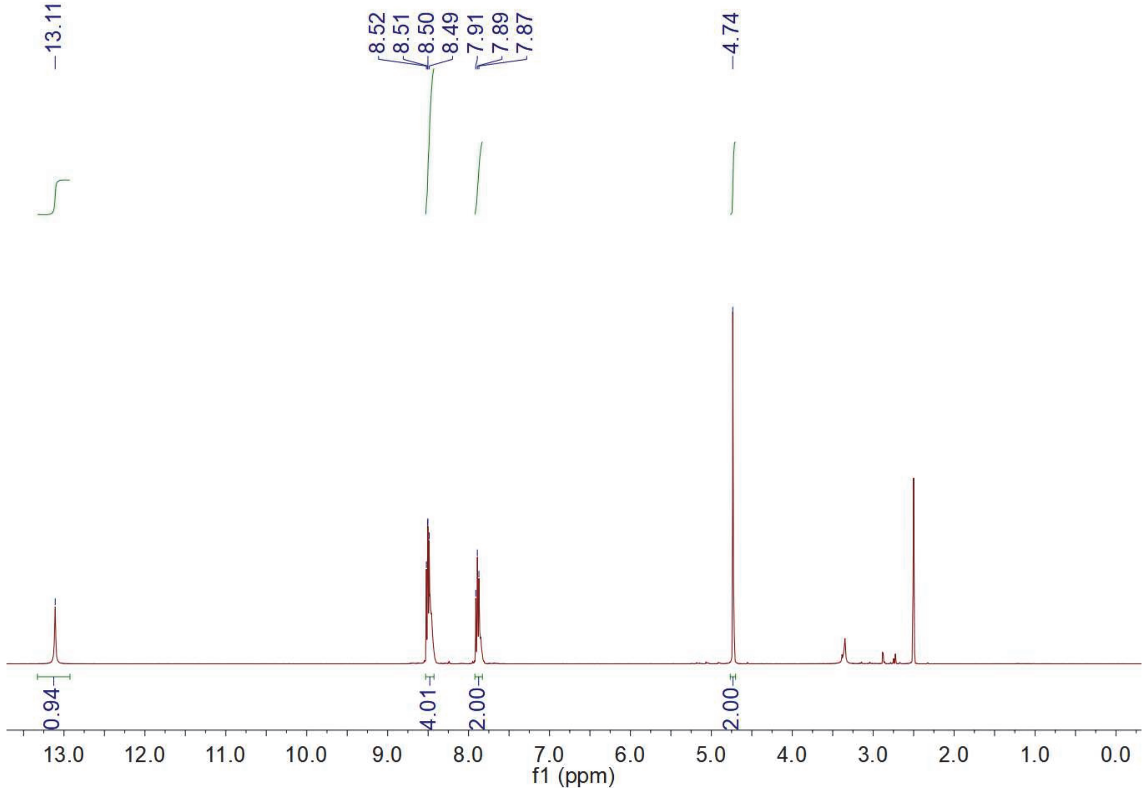

Supplementary Fig. 12 | ^{1}H NMR spectrum. The spectrum of NTIA molecule was collected in DMSO-d_6 under ambient conditions.

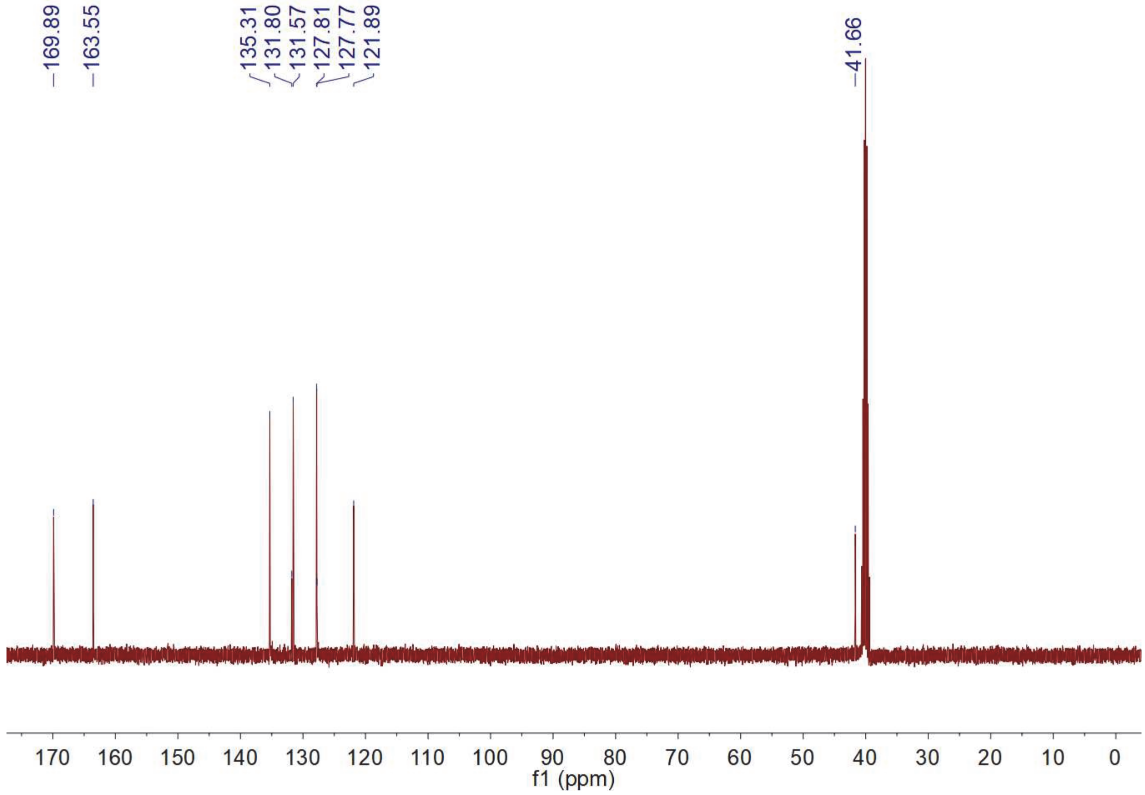

Supplementary Fig. 13 | ^{13}C NMR spectrum. The spectrum of NTIA molecule was collected in DMSO-d_6 under ambient conditions.

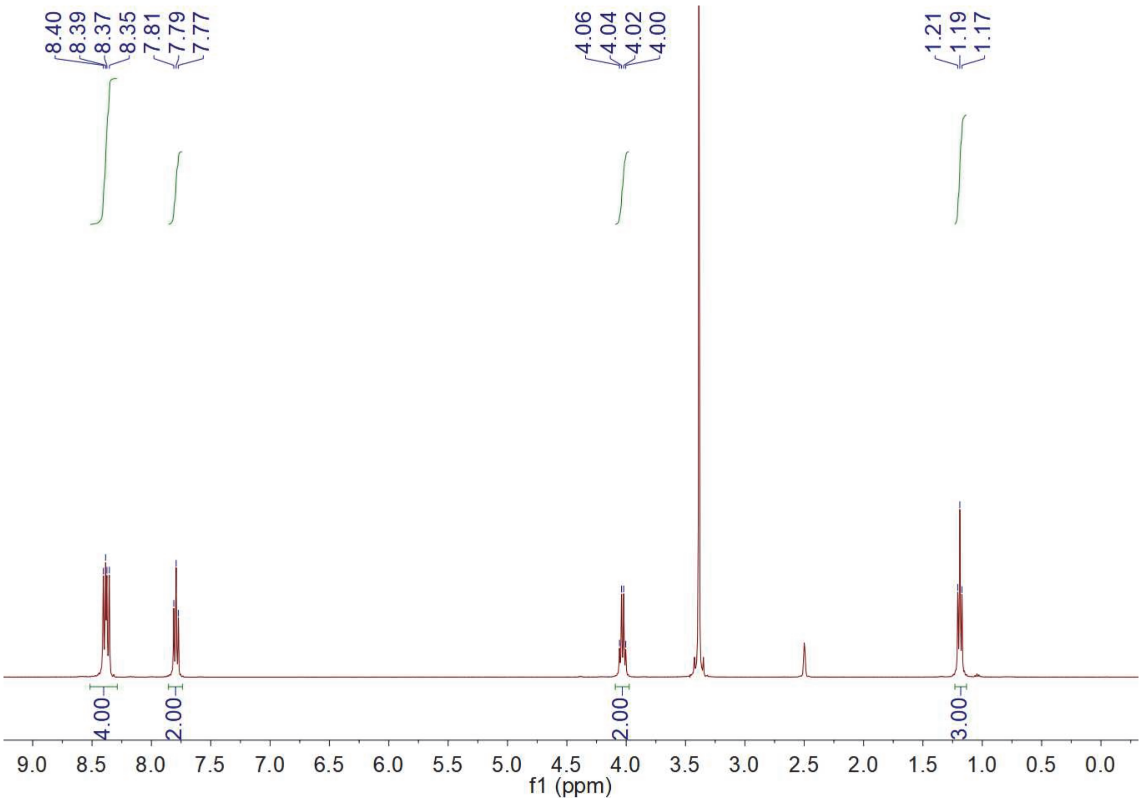

Supplementary Fig. 14 | ^{1}H NMR spectrum. The spectrum of EtNTI molecule was collected in DMSO-d_6 under ambient conditions.

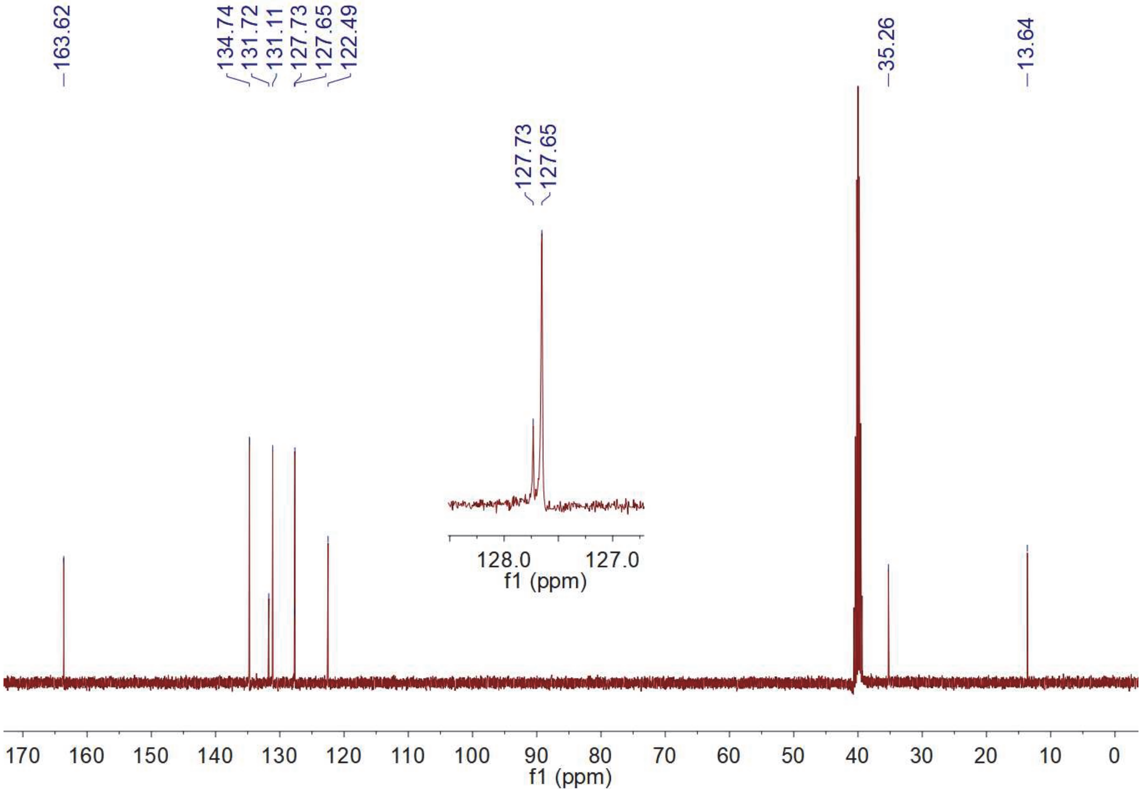

Supplementary Fig. 15 | ^{13}C NMR spectrum. The spectrum of EtNTI molecule was collected in DMSO-d_6 under ambient conditions.

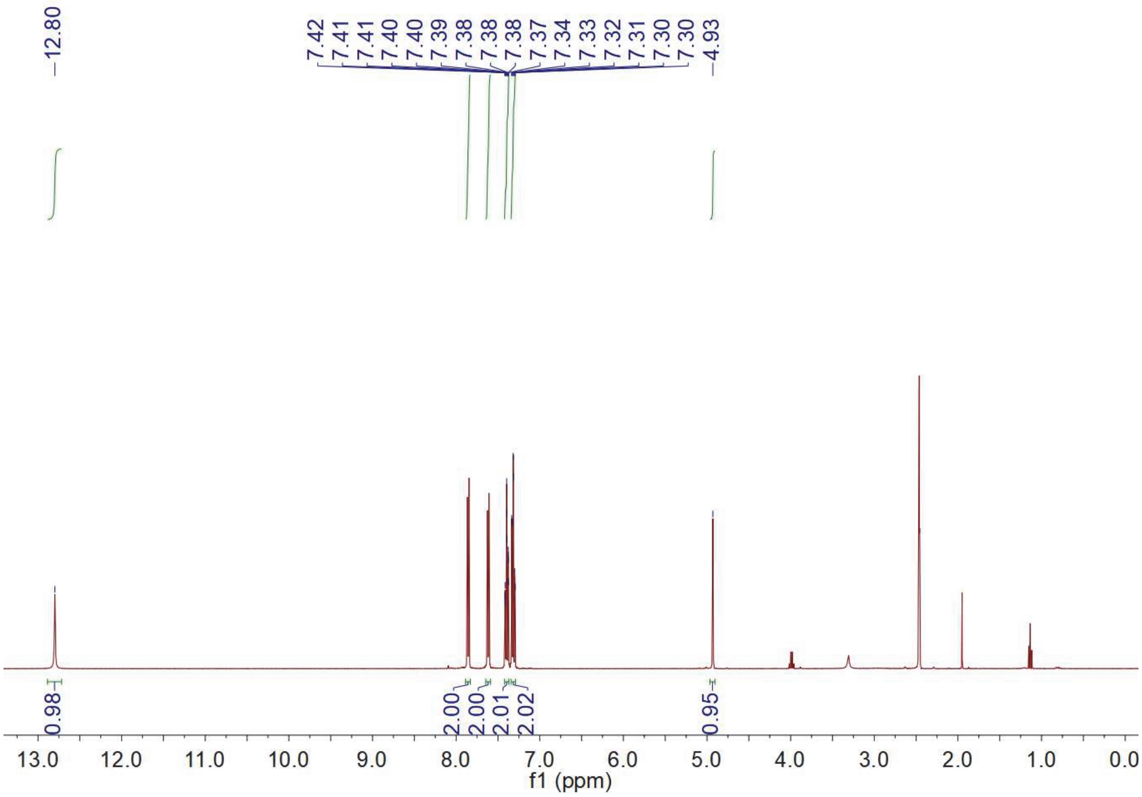

Supplementary Fig. 16 | ^{1}H NMR spectrum. The spectrum of FA molecule was collected in DMSO-d_6 under ambient conditions.

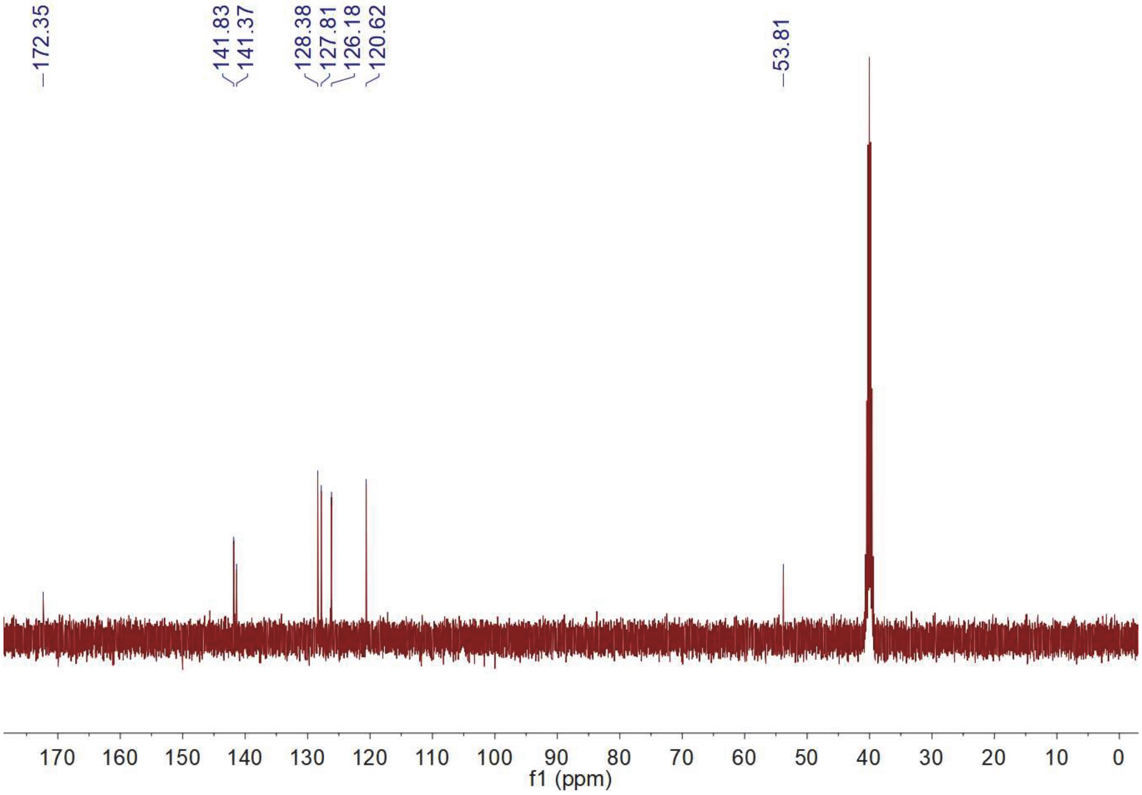

Supplementary Fig. 17 | ^{13}C NMR spectrum. The spectrum of FA molecule was collected in DMSO-d_6 under ambient conditions.

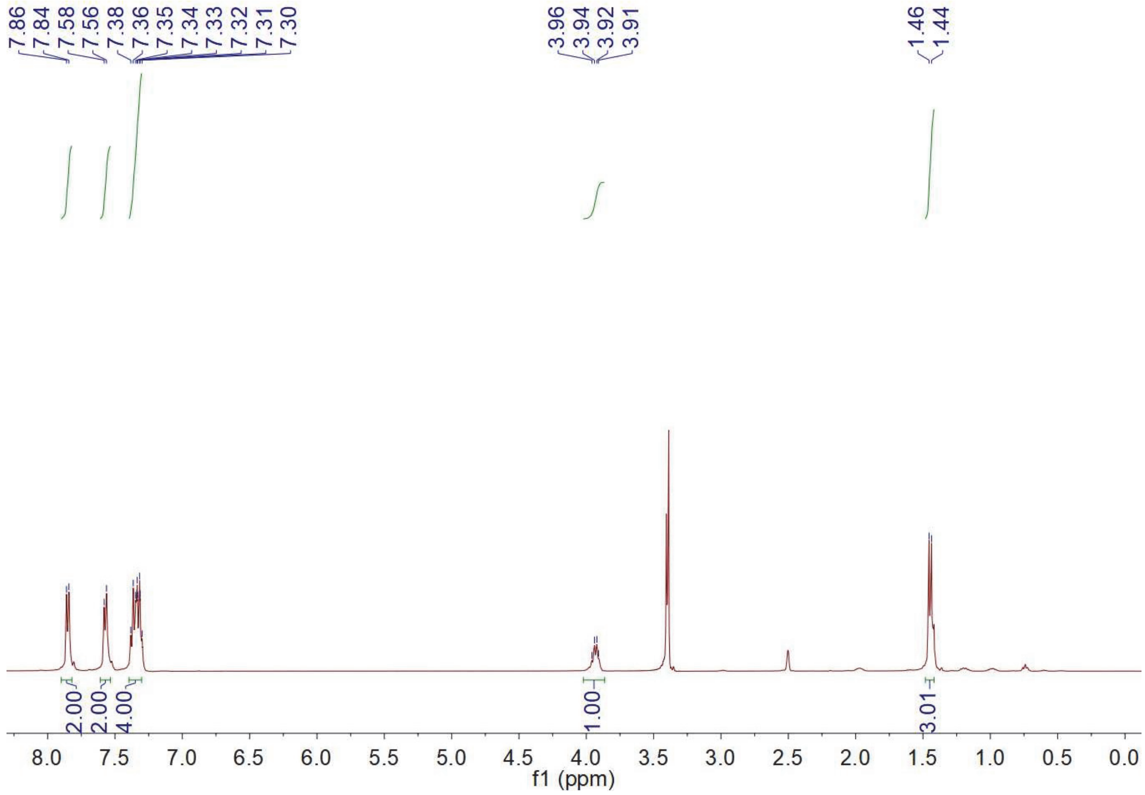

Supplementary Fig. 18 | ^{1}H NMR spectrum. The spectrum of MeF molecule was collected in DMSO-d_6 under ambient conditions.

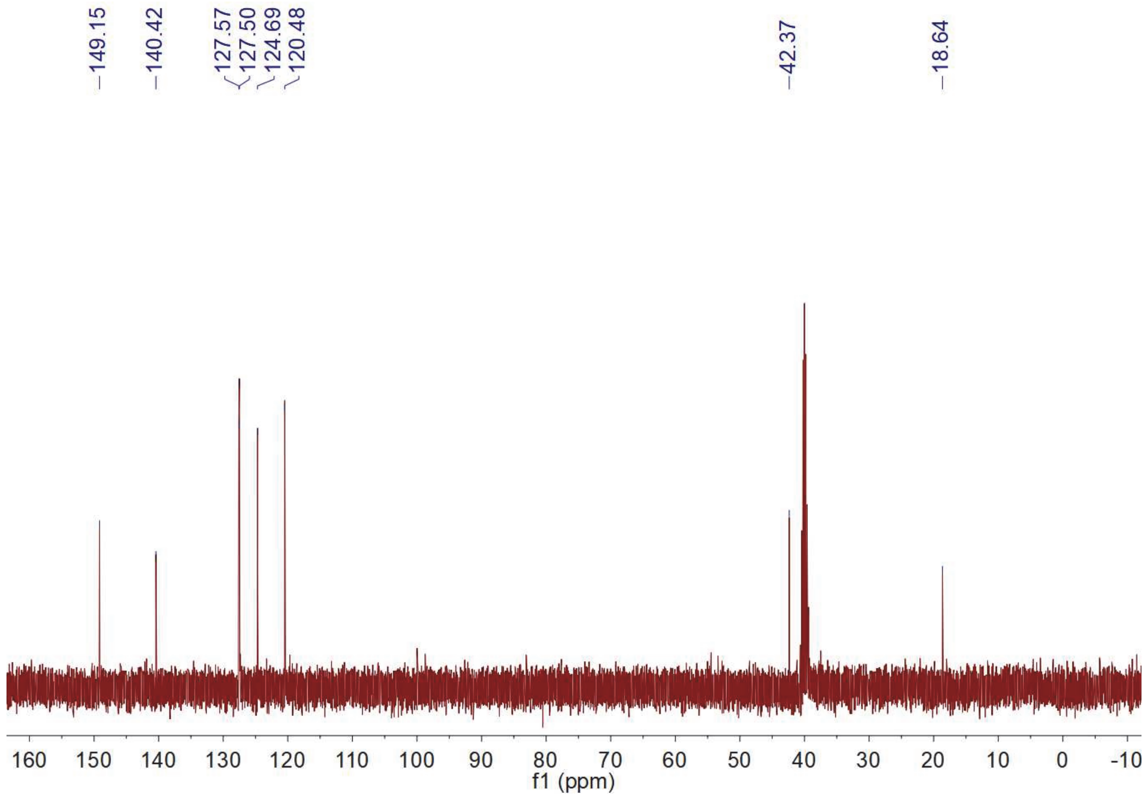

Supplementary Fig. 19 | ^{13}C NMR spectrum. The spectrum of MeF molecule was collected in DMSO-d_6 under ambient conditions.

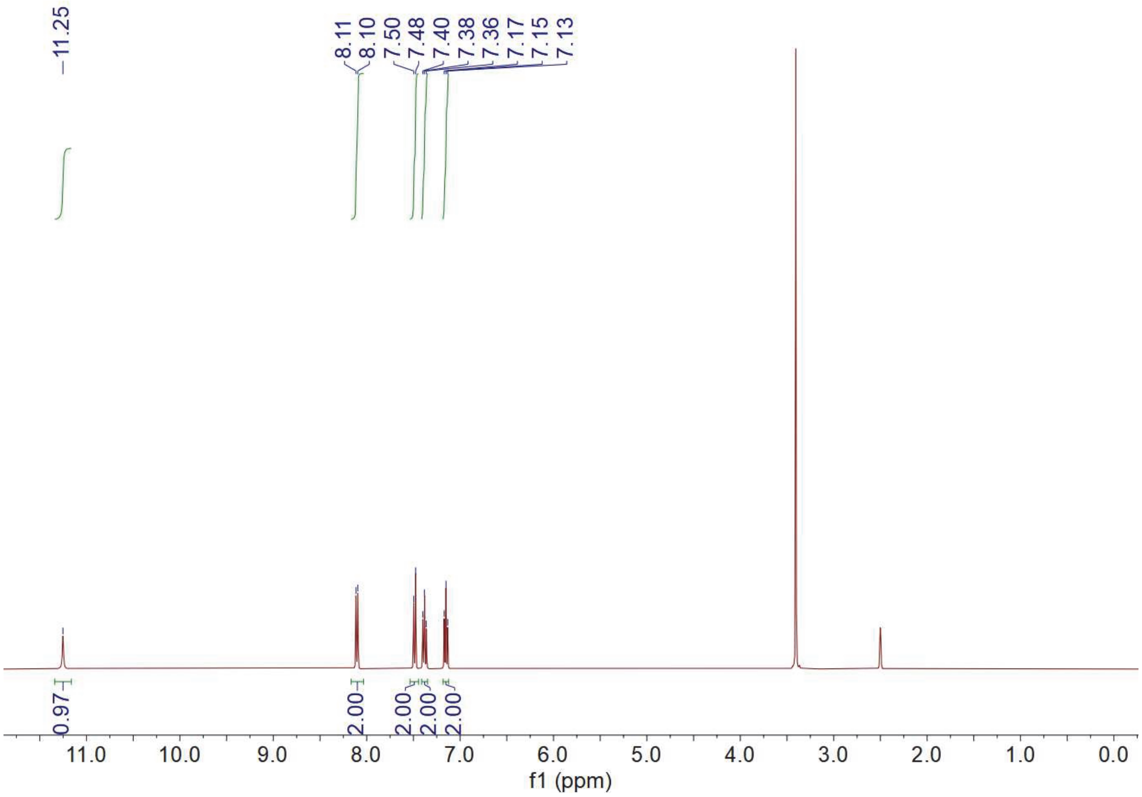

Supplementary Fig. 20 | ^{1}H NMR spectrum. The spectrum of lab-Cz molecule was collected in DMSO-d_6 under ambient conditions.

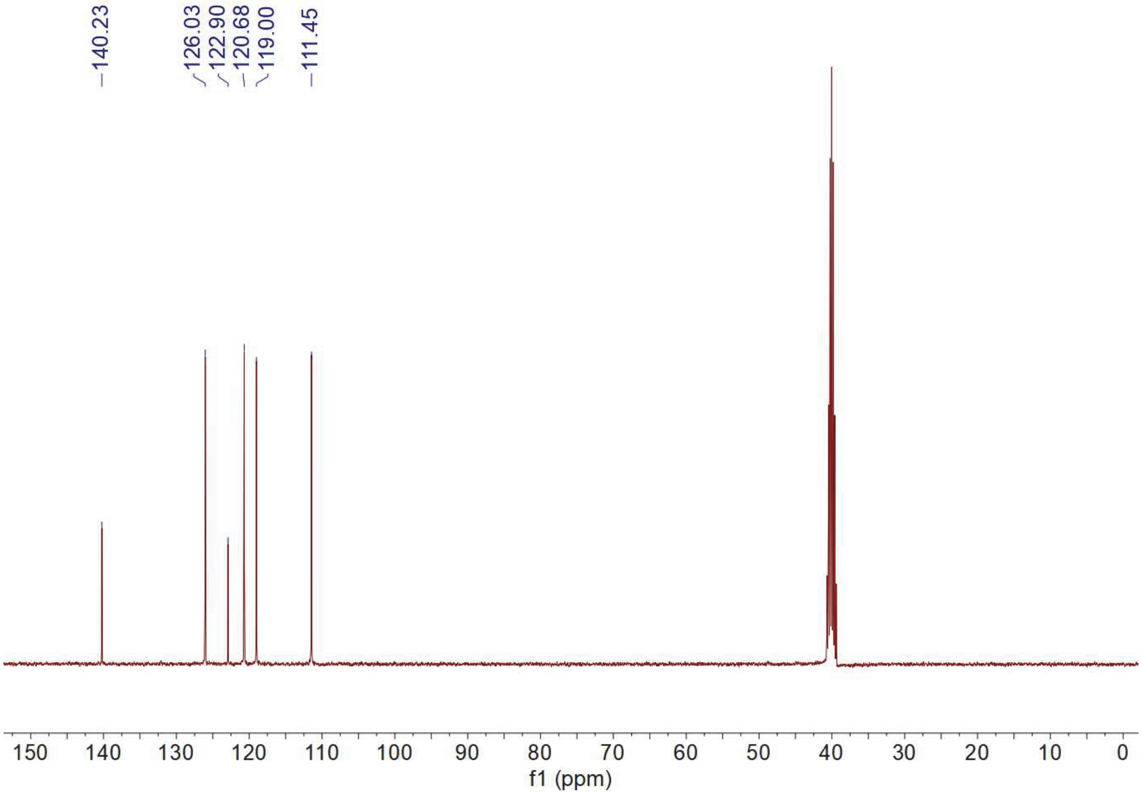

Supplementary Fig. 21 | ^{13}C NMR spectrum. The spectrum of lab-Cz molecule was collected in DMSO-d_6 under ambient conditions.

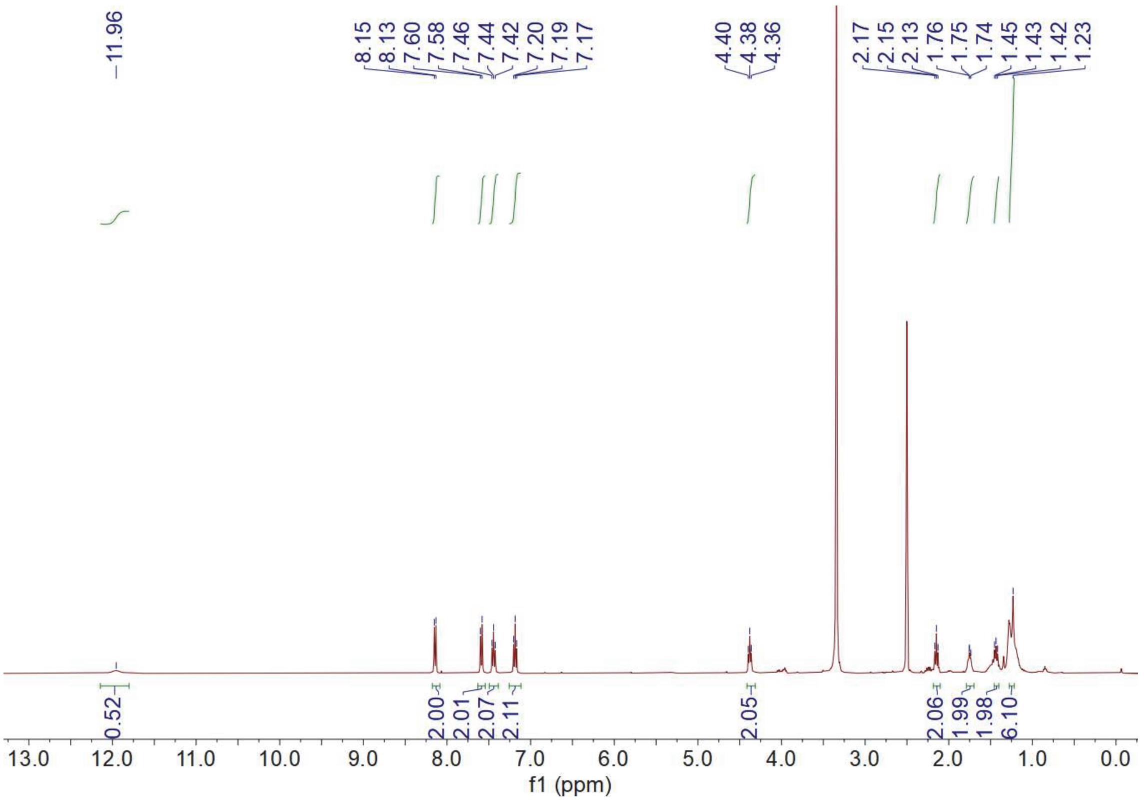

Supplementary Fig. 22 | ^{1}H NMR spectrum. The spectrum of CzOA molecule was collected in DMSO-d_6 under ambient conditions.

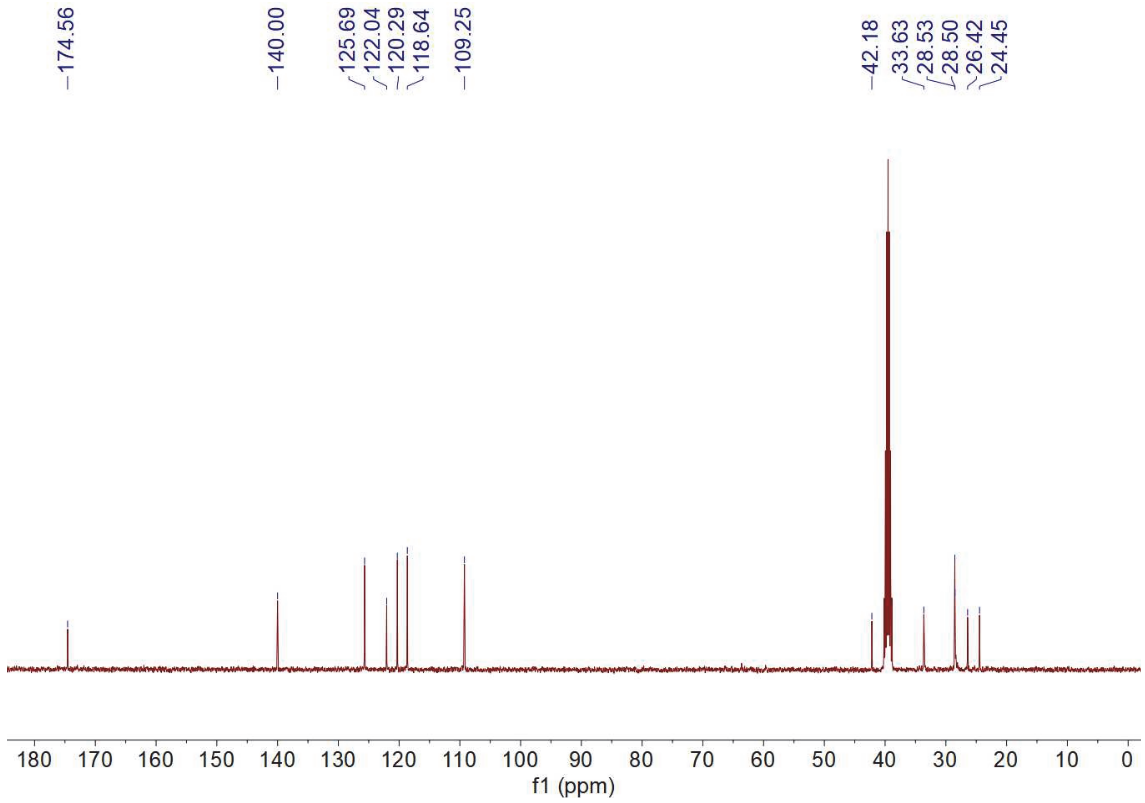

Supplementary Fig. 23 | ^{13}C NMR spectrum. The spectrum of CzOA molecule was collected in DMSO-d_6 under ambient conditions.

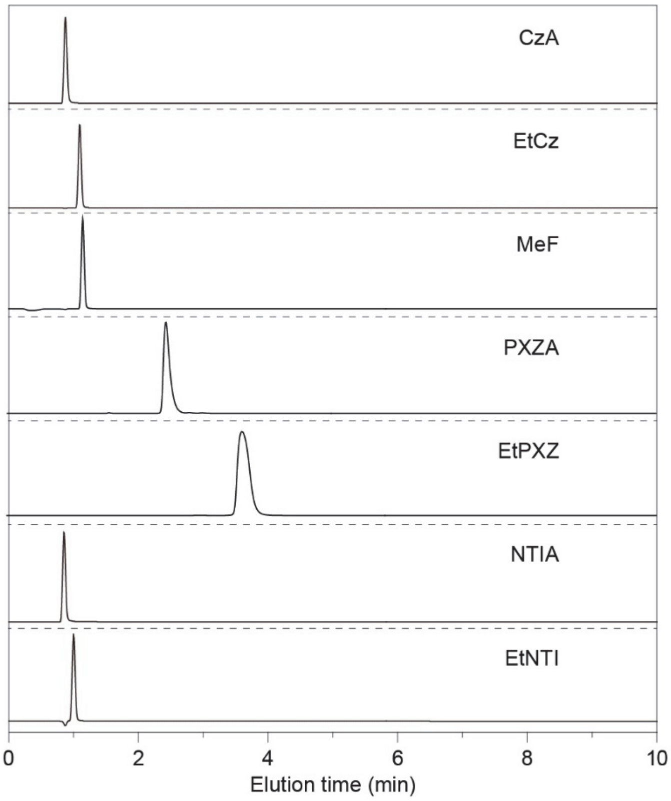

Supplementary Fig. 24 | High performance liquid chromatography (HPLC). From top to bottom, the HPLC spectra of CzA, EtCz, MeF, PXZA, EtPXZ, NTIA and EtNTI were collected in mixed solvent of methanol and water (v/v, 9/1).

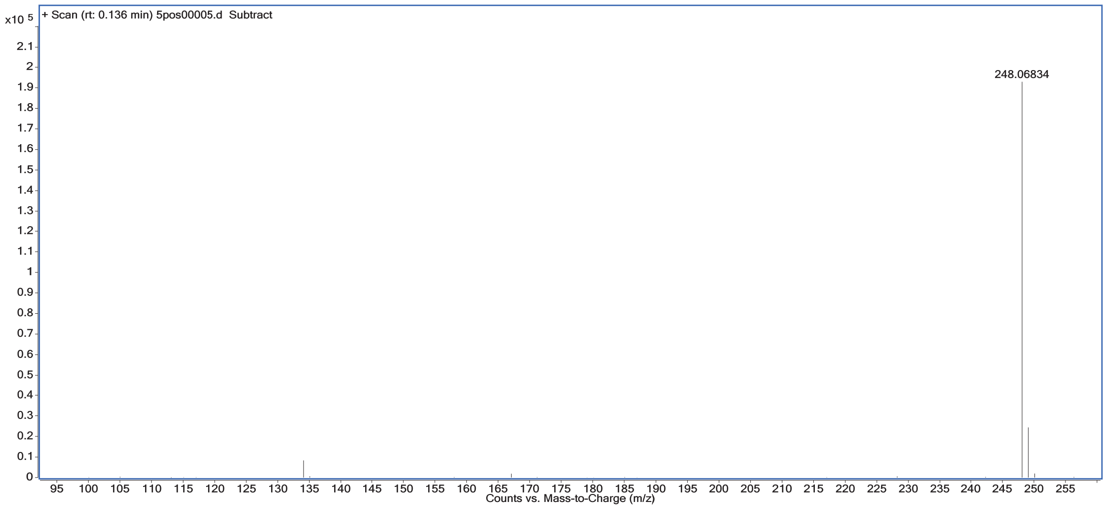

Supplementary Fig. 25 | HRMS of CzA. $(CzA+Na)^+$ m/z: 248.0683 (theoretical value: 248.0687).

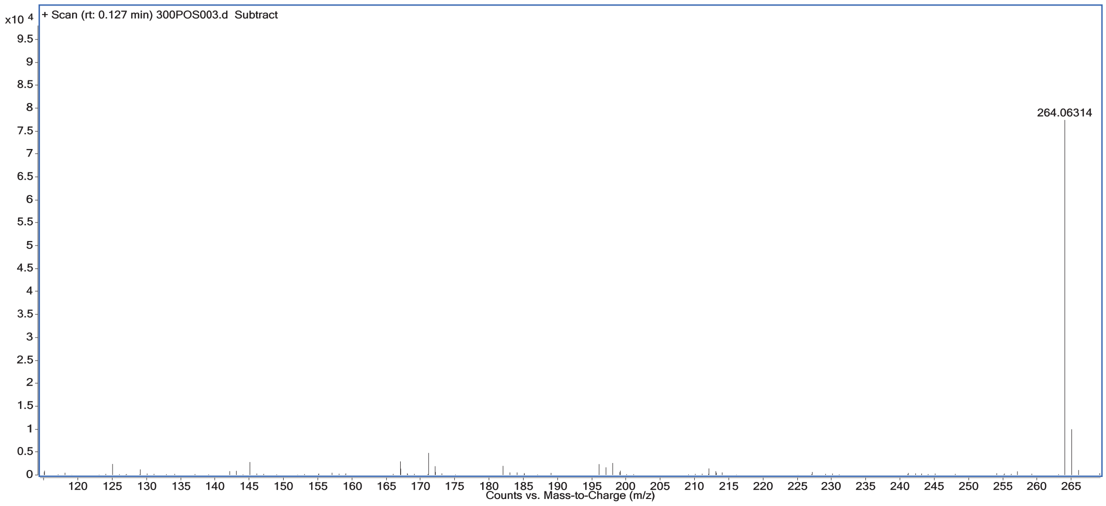

Supplementary Fig. 26 | HRMS of PXZA. $(PXZA+Na)^+$ m/z: 264.0631 (theoretical value: 264.0636).

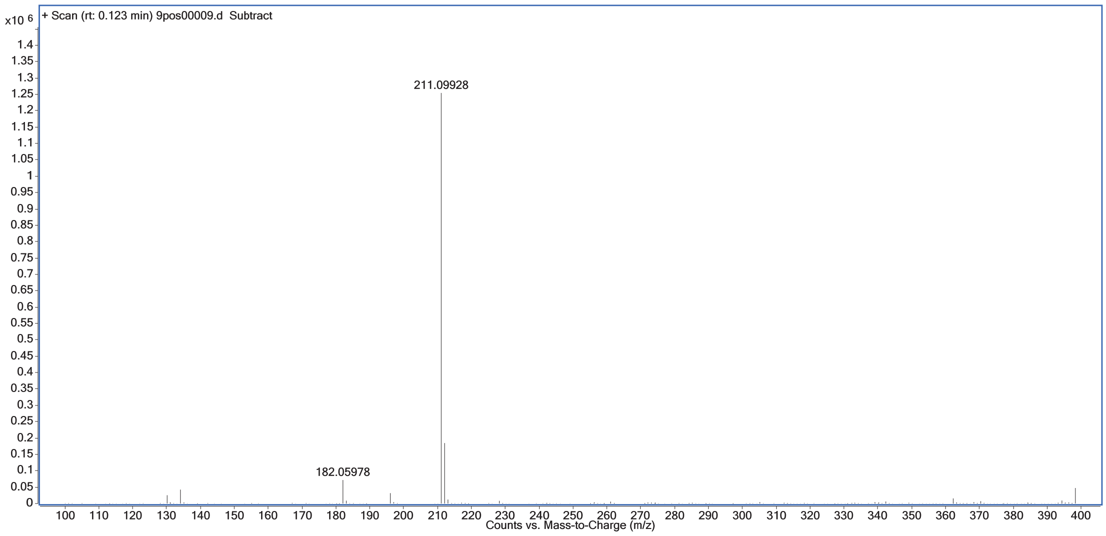

Supplementary Fig. 27 | HRMS of EtPXZ. $(EtPXZ+H)^+$ m/z: 211.0993 (theoretical value: 211.1075).

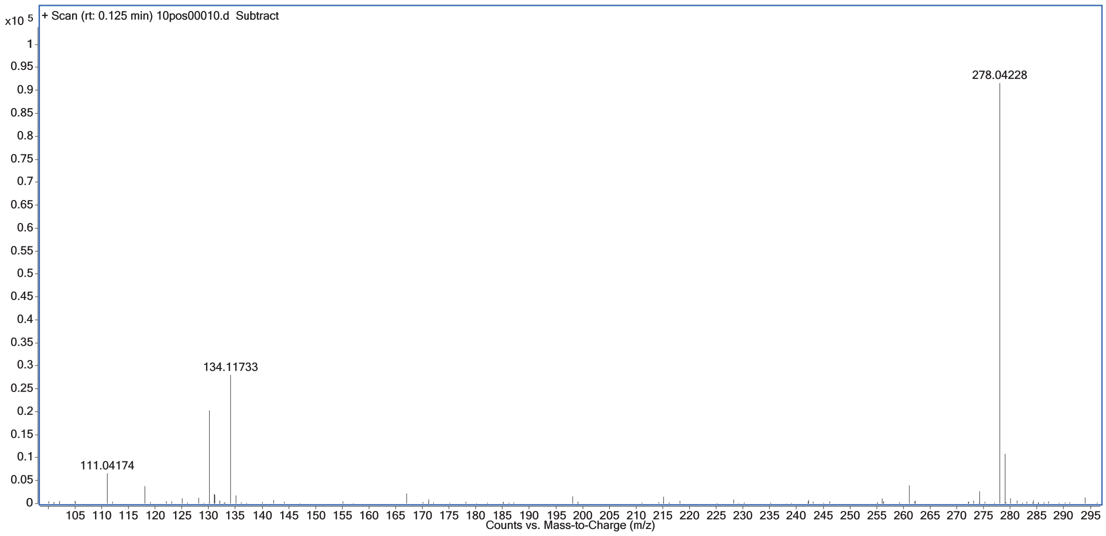

Supplementary Fig. 28 | HRMS of NTIA. $(NTIA+Na)^+$ m/z: 278.0423 (theoretical value: 278.0429).

II. Photophysical properties of organic emitters in film and solution

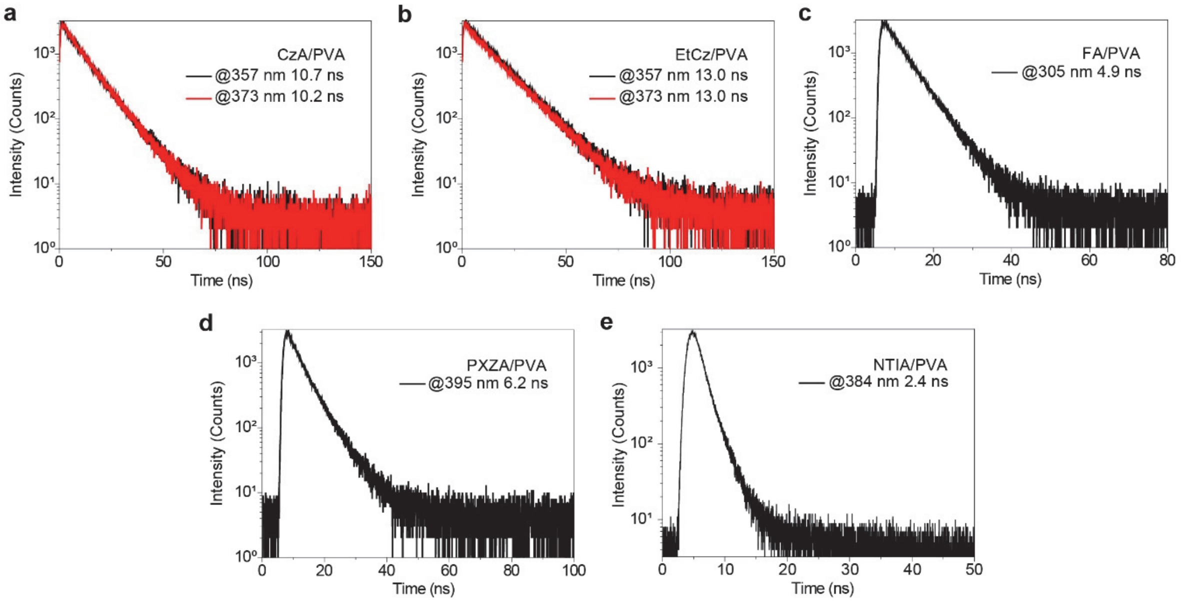

Supplementary Fig. 29 | Lifetime decay profiles of short-lived luminescence of the emitters in film under ambient conditions. a, CzA. **b**, EtCz. **c**, FA. **d**, PXZA. **e,** NTIA.

Supplementary Table 1. Fluorescence lifetimes of the emitters in film under ambient conditions.*

Films	Wavelength (nm)	τ_1 (ns)	A_1 (%)	τ_2 (ns)	A_2 (%)
CzA/PVA	357	3.67	7.34	10.72	96.90
	373	10.21	100.00	–	–
EtCz/PVA	357	13.00	100.00	–	–
	373	13.03	100.00	–	–
FA/PVA	305	4.90	100.00	–	–
PXZA/PVA	395	3.36	50.29	6.18	49.71
NTIA/PVA	384	1.36	88.66	2.98	11.34

*Determined from the fitting function of $I_t = \sum A_i e^{-t/\tau_i}$ according to the fluorescence decay curves, where A_i is the pre-exponential factor for lifetime τ_i.

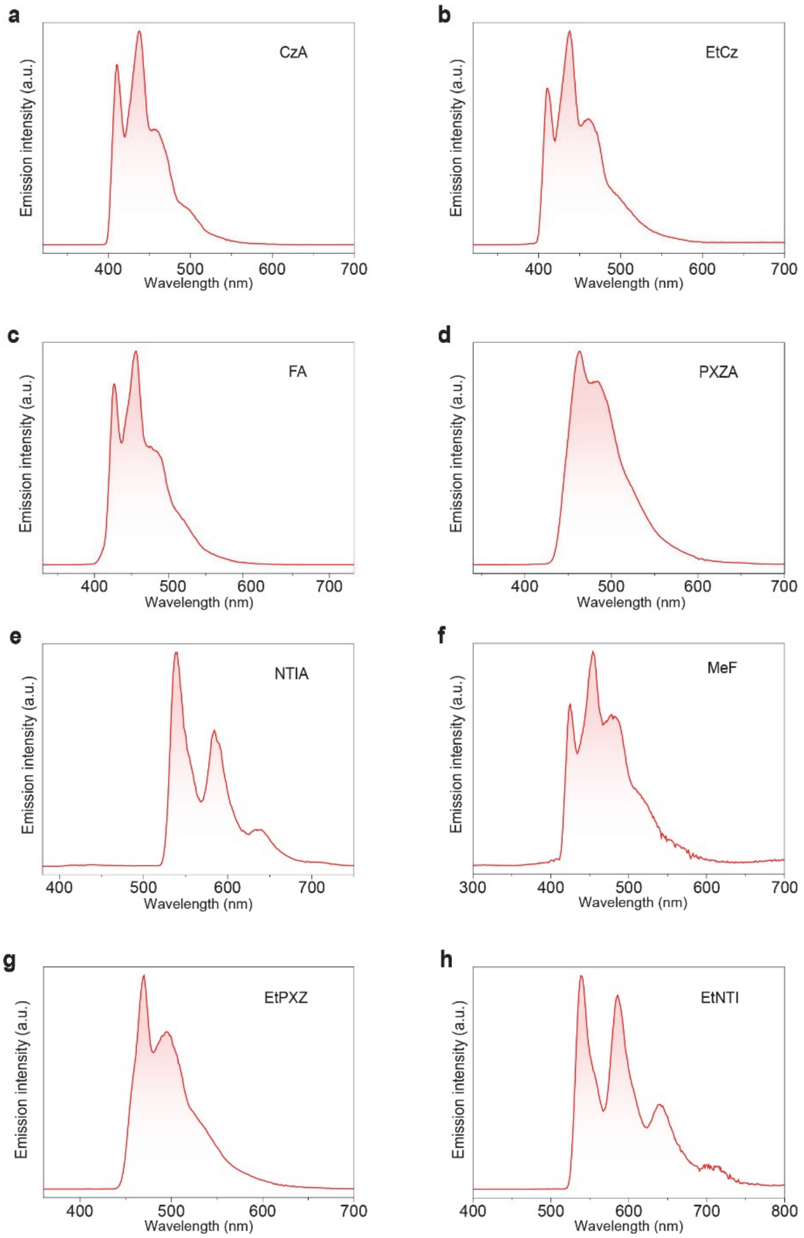

Supplementary Fig. 30 | Normalized phosphorescence spectra of emitters in dilute solution (1 × 10^{-5} M) at 77 K. a, CzA. **b**, EtCz. **c**, FA. **d**, PXZA. **e**, NITA. **f**, MeF. **g**, EtPXZ. **h**, EtNTI. From (**a**) to (**h**), samples were excited by UV light of 300, 300, 290, 320, 360, 280, 340 and 360 nm, respectively.

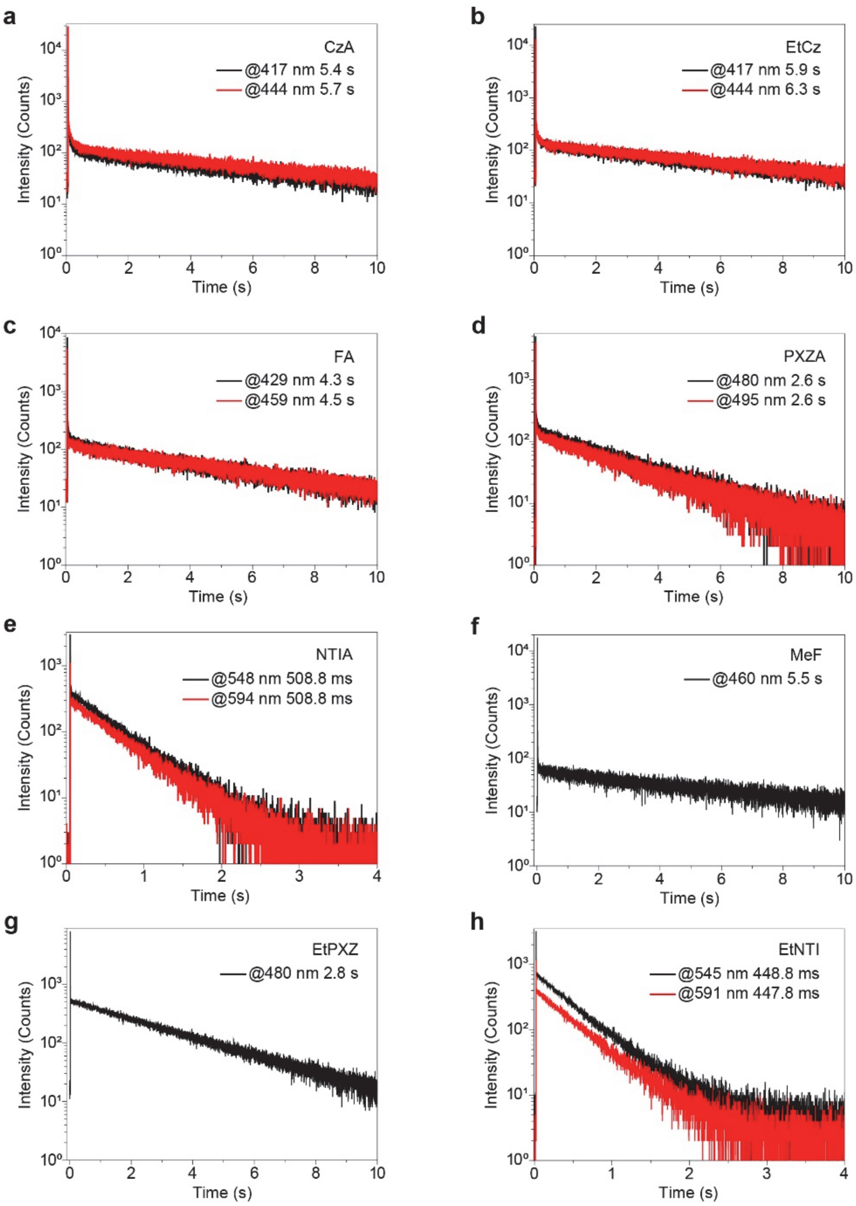

Supplementary Fig. 31 | Decay profiles of long-lived emission for emitters in dilute solution (1 × 10^{-5} M) at 77 K. **a**, CzA. **b**, EtCz. **c**, FA. **d**, PXZA. **e**, NITA. **f**, MeF. **g**, EtPXZ. **h**, EtNTI.

Supplementary Table 2. Phosphorescence lifetimes of the emitters in *m*-THF (1×10^{-5} M) at 77 K.[*]

Compound	Wavelength (nm)	τ_1 (ms)	A_1 (%)
CzA	417	5624.51	100
	444	5418.99	100
EtCz	417	5933.96	100
	444	6255.32	100
FA	429	3916.91	100
	459	4563.80	100
PXZA	480	2567.75	100
	495	2584.14	100
NTIA	546	508.82	100
	592	507.94	100
MeF	460	2584.14	100
EtPXZ	480	2763.28	100
EtNTI	545	448.79	100
	591	447.50	100

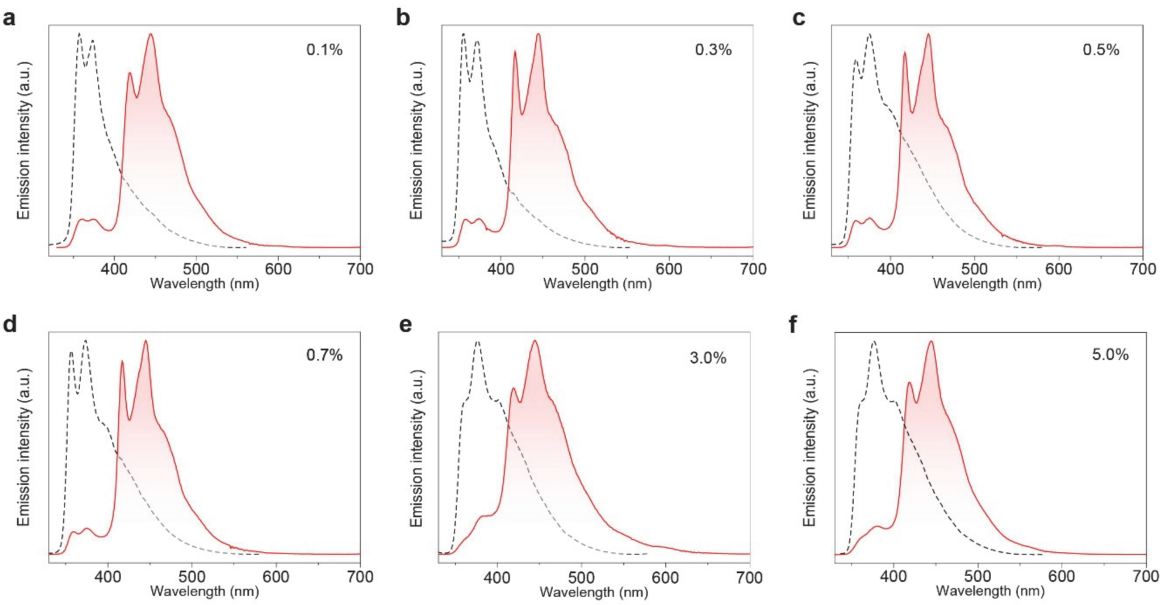

Supplementary Fig. 32 | Normalized steady-state photoluminescence (black dash lines) and phosphorescence (red lines) spectra of CzA in PVA films. **a**, 0.1 wt.%. **b**, 0.3 wt.%. **c**, 0.5 wt.%. **d**, 0.7 wt.%. **e**, 3.0 wt.%. **f**, 5.0 wt.%. Note that the excitation wavelength is 300 nm.

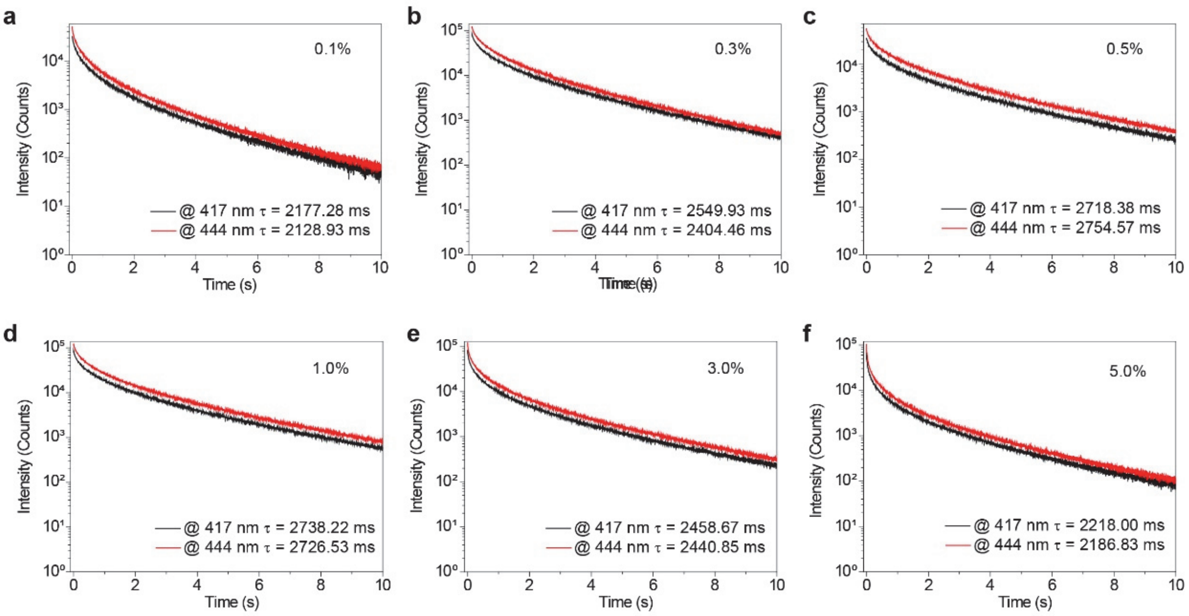

Supplementary Fig. 33 | Lifetime decay profiles of CzA/PVA films monitoring emission bands at 417 and 444 nm under ambient conditions. **a**, 0.1 wt.%. **b**, 0.3 wt.%. **c**, 0.5 wt.%. **d**, 0.7 wt.%. **e**, 3.0 wt.%. **f**, 5.0 wt.%. Note that the excitation wavelength is 300 nm.

Supplementary Table 3. Phosphorescence lifetimes of CzA/PVA films at various ratios under ambient conditions.*

Films	Ratio (%)	Wavelength (nm)	τ_1 (ms)	A_1 (%)	τ_2 (ms)	A_2 (%)	τ_3 (ms)	A_3 (%)	τ_4 (ms)	A_4 (%)
CzA/PVA	0.1	419	44.25	2.57	200.59	14.68	708.59	38.46	2177.28	44.29
		444	44.72	2.79	207.42	15.87	706.53	37.68	2128.93	43.66
	0.3	417	44.43	1.11	235.74	9.07	845.11	31.33	2549.93	58.49
		444	39.43	1.01	215.51	8.56	786.79	31.75	2404.46	58.68
	0.5	417	40.42	0.90	226.30	7.18	832.43	27.74	2718.38	64.19
		444	44.69	0.88	239.15	7.71	874.56	28.81	2754.57	62.60
	0.7	417	35.39	0.80	211.63	7.20	813.61	28.03	2720.57	63.98
		444	36.85	0.86	224.87	7.81	845.58	28.76	2755.06	62.57
	1.0	417	38.11	1.02	219.88	8.46	827.25	29.02	2738.22	61.50
		444	39.17	1.04	221.36	8.49	826.18	29.09	2726.53	61.39
	3.0	417	24.70	1.74	165.06	11.39	683.44	32.76	2458.67	54.11
		444	26.95	1.83	167.62	11.50	686.01	32.54	2440.85	54.13
	5.0	418	23.13	2.80	141.32	14.45	597.45	34.23	2218.00	48.52
		444	22.26	2.98	138.86	14.52	590.53	34.36	2186.83	48.14

*Determined from the fitting function of $I_t = \sum A_i e^{-t/\tau_i}$ according to the phosphorescence decay curves, where A_i is the pre-exponential factor for lifetime τ_i.

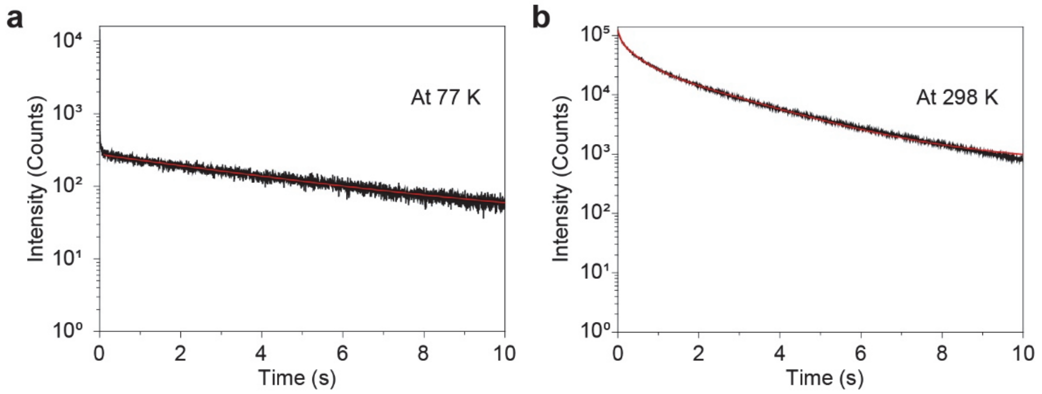

Supplementary Fig. 34 | Lifetime decay curves of CzA in PVA matrix at different temperature. a, At 77 K. **b,** At 298 K.

Supplementary Table 4. Phosphorescence lifetimes of CzA in PVA matrix at 77 K and room temperature. *

Temperature (K)	τ_1 (ms)	A_1 (%)	τ_2 (ms)	A_2 (%)	τ_3 (ms)	A_3 (%)	τ_4 (ms)	A_4 (%)
77	5413.75	100	-	-	-	-	-	-
298	39.17	1.04	221.36	8.49	826.18	29.09	2726.53	61.39

*Determined from the fitting function of $I_t = \sum A_i e^{-t/\tau_i}$ according to the phosphorescence decay curves, where A_i is the pre-exponential factor for lifetime τ_i.

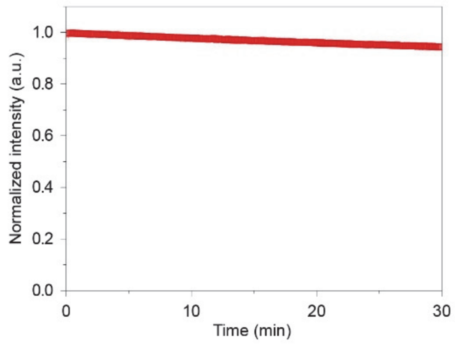

Supplementary Fig. 35 | Photo-stability of the CzA/PVA film under UV light. Phosphorescence intensity of the CzA/PVA film was recorded as a function of time at 298 K with a continuous 300 nm UV lamp excitation.

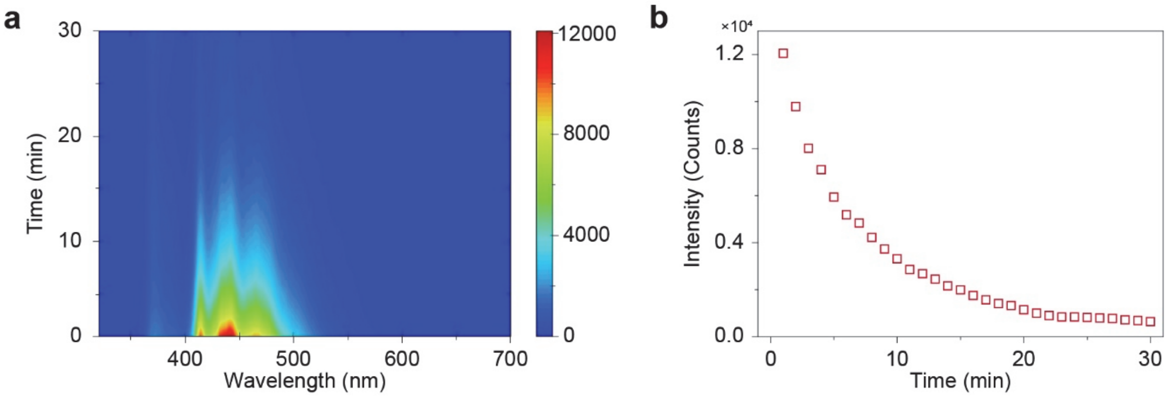

Supplementary Fig. 36 | Phosphorescent stability of the CzA/PVA film under high relative humidity atmosphere. a, Time-phosphorescence emission mapping of the CzA/PVA film exposed to high relative humidity (68%) at room temperature within 30 min. **b**, Phosphorescence intensity as a function of time by monitoring the peak emission.

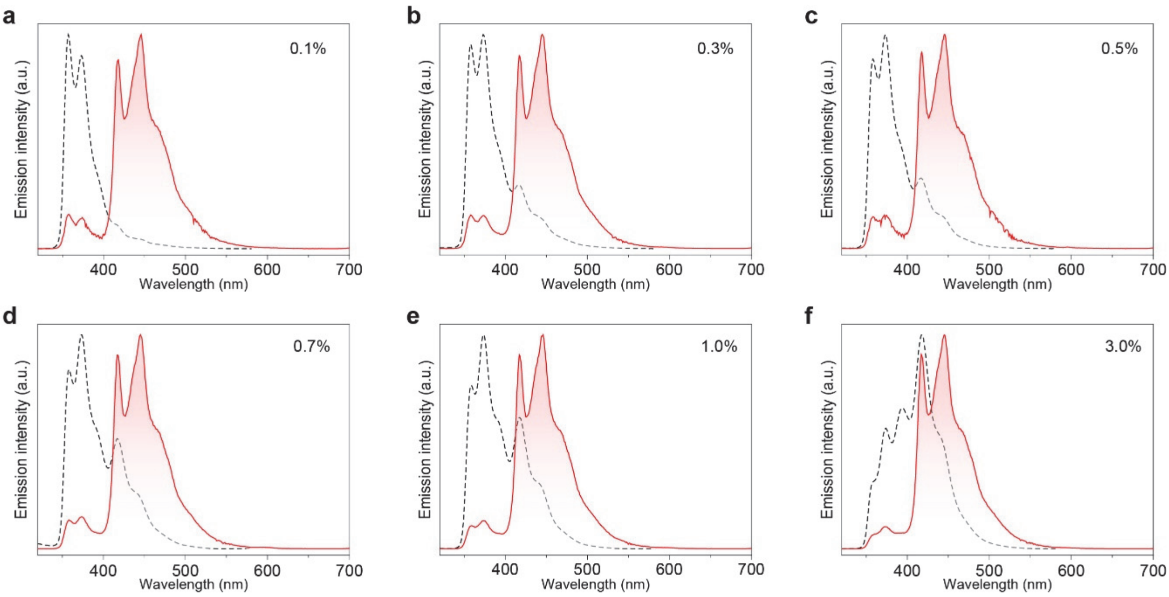

Supplementary Fig. 37 | Normalized steady-state photoluminescence (black dash lines) and phosphorescence (red lines) spectra of EtCz/PVA films at various doped ratios (wt./wt.) upon excitation of 300 nm UV light under ambient conditions. a, 0.1 wt.%. **b**, 0.3 wt.%. **c**, 0.5 wt.%. **d**, 0.7 wt.%. **e**, 1.0 wt.%. **f**, 3.0 wt.%.

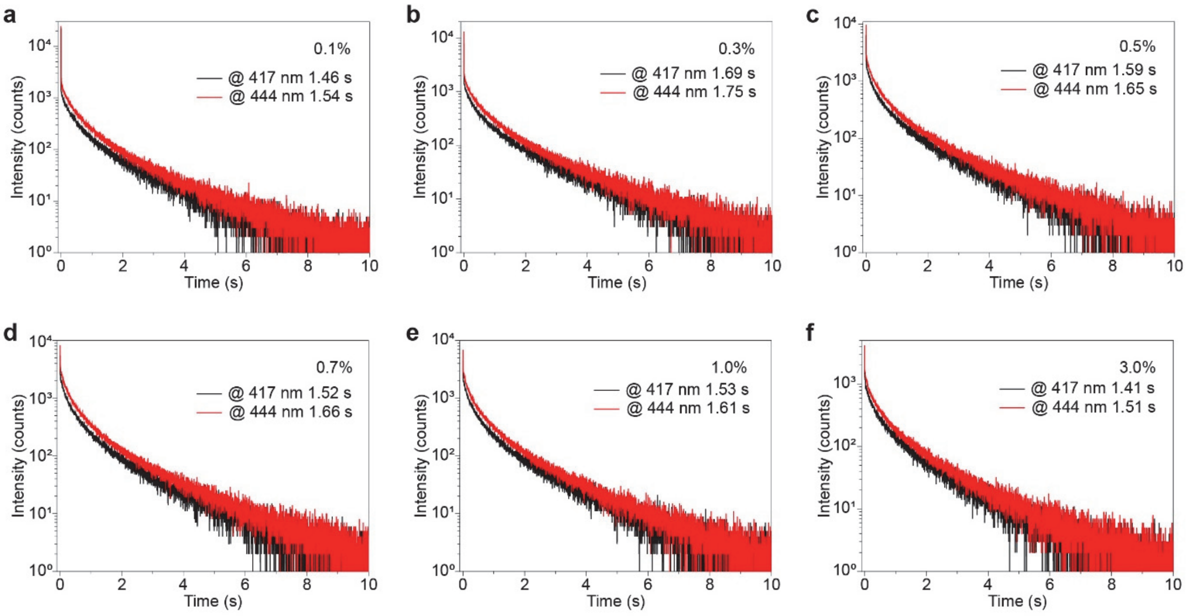

Supplementary Fig. 38 | Lifetime decay profiles of the phosphorescence emission at 417 and 444 nm for EtCz/PVA films at various doped ratios (wt./wt.) under ambient conditions. **a**, 0.1 wt.%. **b**, 0.3 wt.%. **c**, 0.5 wt.%. **d**, 0.7 wt.%. **e**, 1.0 wt.%. **f**, 3.0 wt.%. The excitation wavelength is 300 nm.

Supplementary Table 5. Phosphorescence lifetimes of EtCz/PVA films at various ratios under ambient conditions.*

Films	Ratio (%)	Wavelength (nm)	τ_1 (ms)	A_1 (%)	τ_2 (ms)	A_2 (%)	τ_3 (ms)	A_3 (%)
EtCz/PVA	0.1	417	94.29	7.65	424.14	43.41	1461.56	48.94
		444	105.00	9.73	463.42	44.51	1539.95	45.76
	0.3	417	114.27	10.03	506.98	39.13	1691.13	50.84
		444	107.35	9.01	510.46	42.05	1751.95	48.94
	0.5	417	91.22	10.87	435.04	41.89	1590.79	47.25
		444	99.42	11.84	453.49	43.09	1648.69	45.07
	0.7	417	93.04	12.18	422.81	43.02	1524.14	44.80
		444	100.95	13.28	462.66	45.56	1657.95	41.16
	1.0	417	83.87	10.11	405.70	42.83	1525.30	47.06
		444	103.72	12.98	457.15	43.45	1612.34	43.57
	3.0	418	58.55	5.62	356.65	36.65	1408.46	57.73
		444	84.16	8.07	417.88	38.51	1512.15	53.42

*Determined from the fitting function of $I_t = \sum A_i e^{-t/\tau_i}$ according to the phosphorescence decay curves, where A_i is the pre-exponential factor for lifetime τ_i.

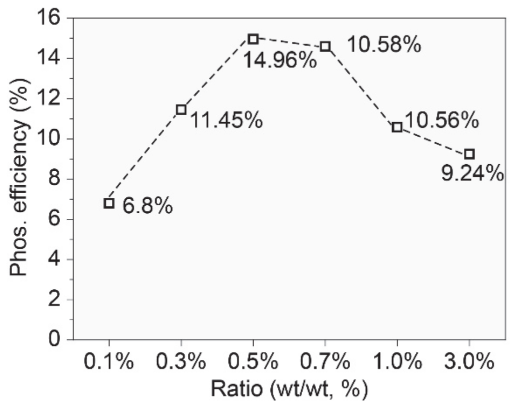

Supplementary Fig. 39 | The relationship between phosphorescence efficiencies and doping ratios. Phosphorescence efficiencies of EtCz/PVA films at various doped ratios (wt./wt.) was collected under ambient conditions.

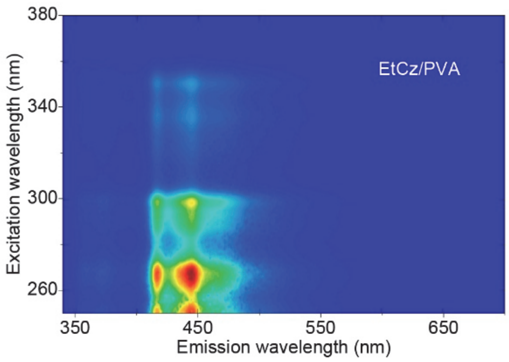

Supplementary Fig. 40 | Excitation-phosphorescence emission mapping under ambient conditions. The concentration of EtCz molecule in PVA film is of 0.7 wt.%.

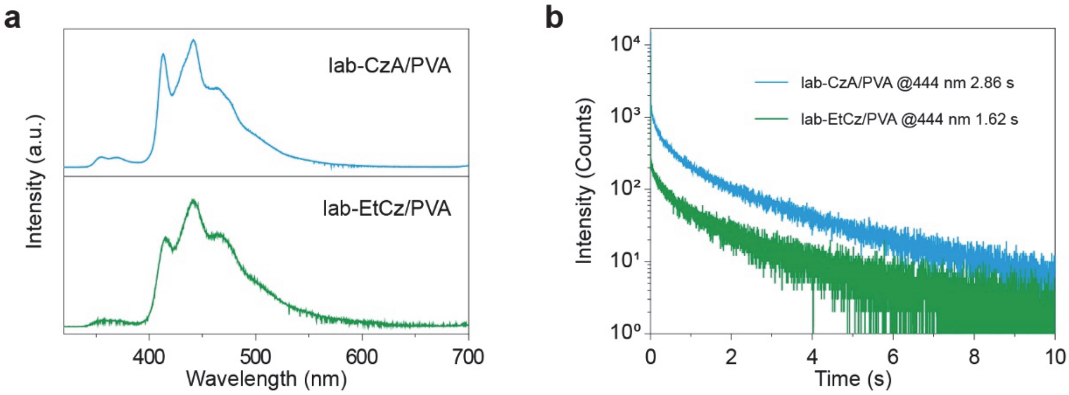

Supplementary Fig. 41 | Phosphorescence properties of CzA and EtCz based on synthesized carbazole in PVA films under ambient conditions. a, Normalized phosphorescence spectra of lab-CzA/PVA (1.0 wt.%) and lab-EtCz/PVA (0.5 wt.%) films. **b,** Lifetime decay curves of lab-CzA/PVA (1.0 wt. %) and lab-EtCz/PVA (0.5 wt.%) films. Note that the excitation wavelength is 300 nm.

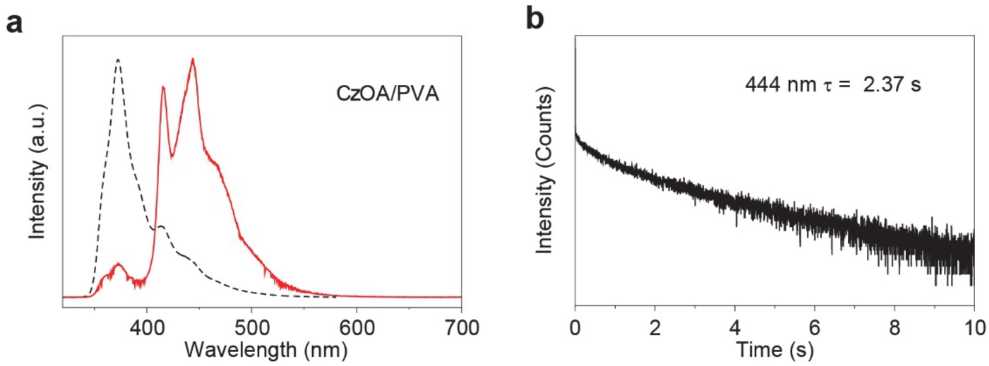

Supplementary Fig. 42 | Photophysical properties of CzOA in PVA film under ambient conditions. a, Steady-state photoluminescence (dash lines) and phosphorescence (solid lines) spectra of CzOA/PVA. **b**, Lifetime decay profile of CzOA in PVA. The concentration of CzOA molecule in PVA matrix is 0.5 wt.%.

III. Understanding the relationship between ^{1}H NMR chemical shift and intermolecular interactions

Chemical shift is defined as[1]:

$$\delta_i = \frac{\upsilon_{sample} - \upsilon_{TMS}}{\upsilon_{spectrometer}} \times 10^6 \quad \text{(Supplementary Equation S1)}$$

$\upsilon_{spectrometer}$ means the spectrometer frequency in MHz.

υ_{sample} is influence on the shielding constant σ, which can be expressed as[2]:

$$\upsilon_{sample} = \frac{\Upsilon}{2\pi} B_0(1-\sigma) \quad \text{(Supplementary Equation S2)}$$

Υ magnetogyric ratio of atom

B_0 magnetogyric ratio

$$\sigma = \sigma_{Pauli} + \sigma_{oi} \quad \text{(Supplementary Equation S3)}$$

^{1}H-shielding values (σ) of H are governed by the Pauli repulsion interaction, which originates from the fact that electrons with the same spin are not allowed to be at the same position in space, and are a manifestation of the Pauli principle[3].

In conclude, the increase of the σ_{Pauli} value will cause the decrease of δ_i value. That is, Pauli repulsive interaction will cause upfield of the ^{1}H NMR chemical shift.

IV. FT-IR, NMR, PXRD and TGA data for mechanism investigation

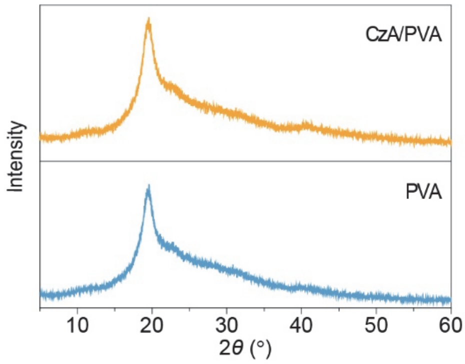

Supplementary Fig. 43 | Experimental PXRD patterns of CzA/PVA and pure PVA films under ambient conditions. The concentration of CzA in PVA matrix is 1.0 wt.%.

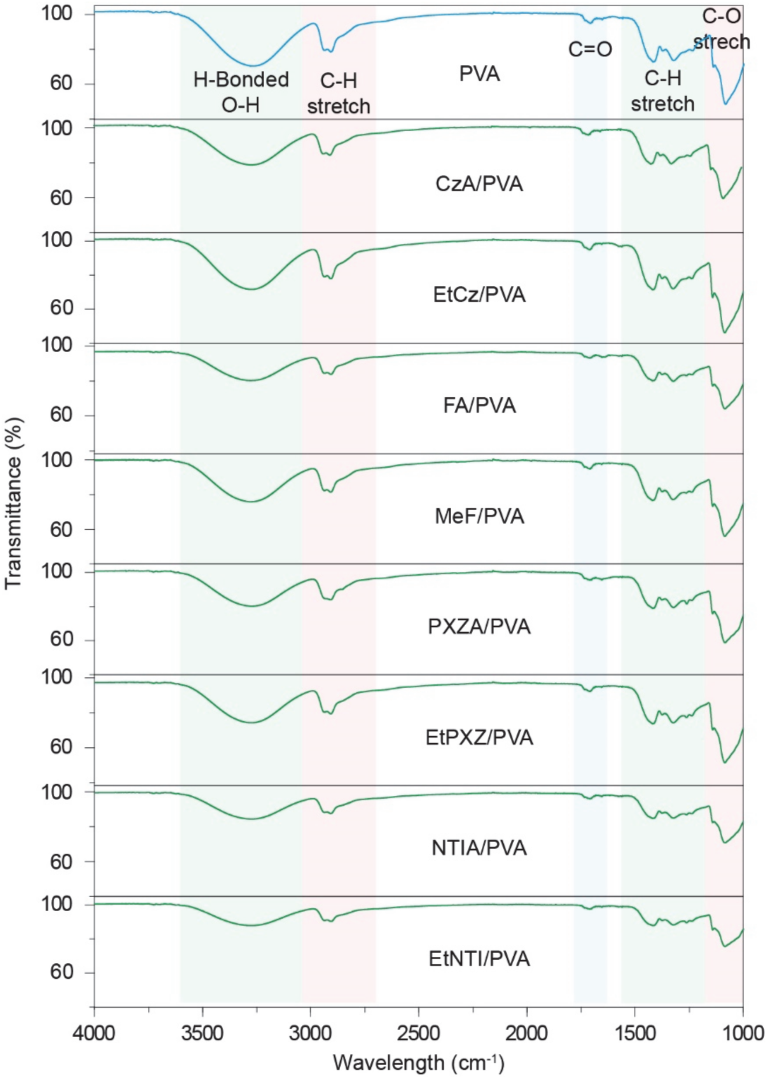

Supplementary Fig. 44 | FT-IR spectra of neat PVA and PVA films with various emitters. Note that the concentrations of emitters in PVA matrix were optimized firstly. The concentrations of CzA, EtCz, FA, MeF, PXZA, EtPXZ, NTIA and EtNTI were 1.0 wt.%, 0.5 wt.%, 0.3 wt.%, 0.3 wt.%, 1.0 wt.%, 1.0 wt.%, 0.5 wt.% and 0.5%, respectively.

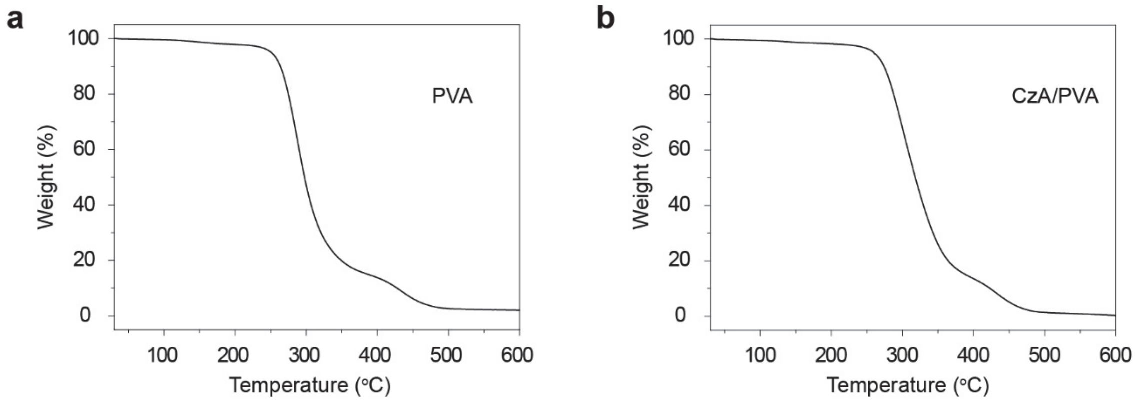

Supplementary Fig. 45 | Thermogravimetric analysis. a, PVA matrix. **b**, CzA/PVA (1.0 wt.%) films.

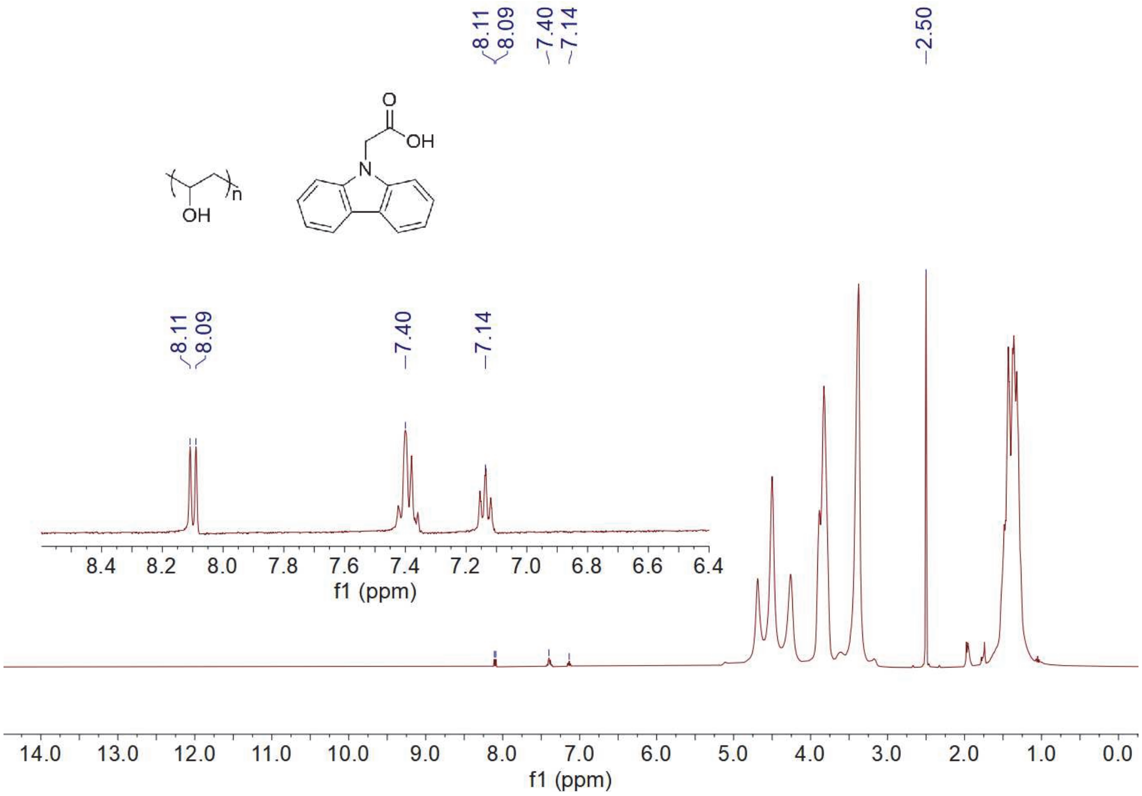

Supplementary Fig. 46 | ¹H NMR spectrum of CzA/PVA films in DMSO-*d*₆. The concentration of CzA is 1.0 wt.%.

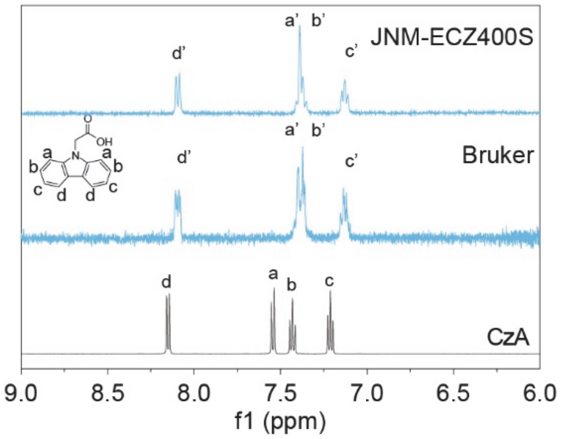

Supplementary Fig. 47 | Chemical shift of aromatic hydrogen for CzA molecule in PVA matrix. The spectra were measured in DMSO-d_6 by JNM-ECZ400S (top) and Bruker (middle), respectively.

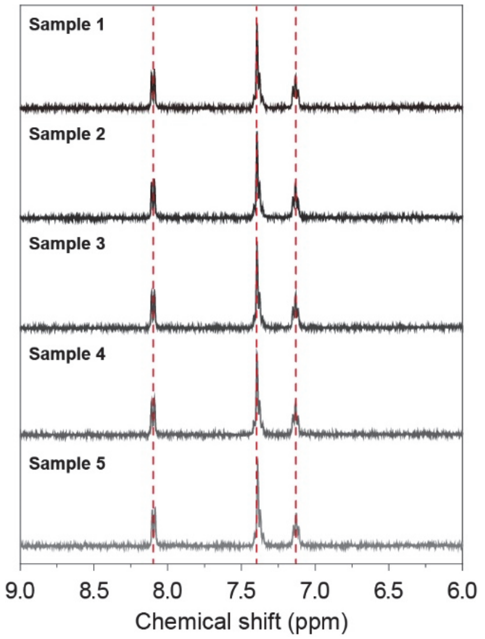

Supplementary Fig. 48 | Repetitive experiments for the chemical shift of aromatic hydrogen of CzA molecule in PVA matrix. The concentration of CzA is 1.0 wt.% in DMSO-d_6. The spectra were measured by JNM-ECZ400S.

To exclude the instrumental error, we repeated the NMR test in different instruments (Bruker Ultra Shield spectrometer 500 MHz and JNM-ECZ400S spectrometer 400 MHz) many times. From Supplementary Fig. 47, it is worth noting that there is no change of chemical shift in the ^{1}H NMR spectra of CzA/PVA from the two instruments. In addition, we also collected the ^{1}H NMR spectra of five different samples of the CzA/PVA films. There is no change in the chemical shift of aromatic hydrogen. The average varied values of chemical shifts for four types of hydrogens are 0.16 (H_a), 0.04 (H_b), 0.09 (H_c), and 0.07 (H_d) ppm, after CzA is doped into PVA (Supplementary Fig. 48). In conclusion, the chemical shift is caused by molecular interactions rather than instrument error.

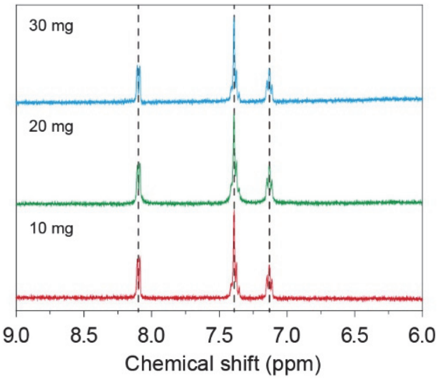

Supplementary Fig. 49 | Partial ^{1}H NMR spectra of CzA/PVA in DMSO-*d*$_6$. The concentration of the CzA/PVA films were 10, 20 and 30 mg, respectively.

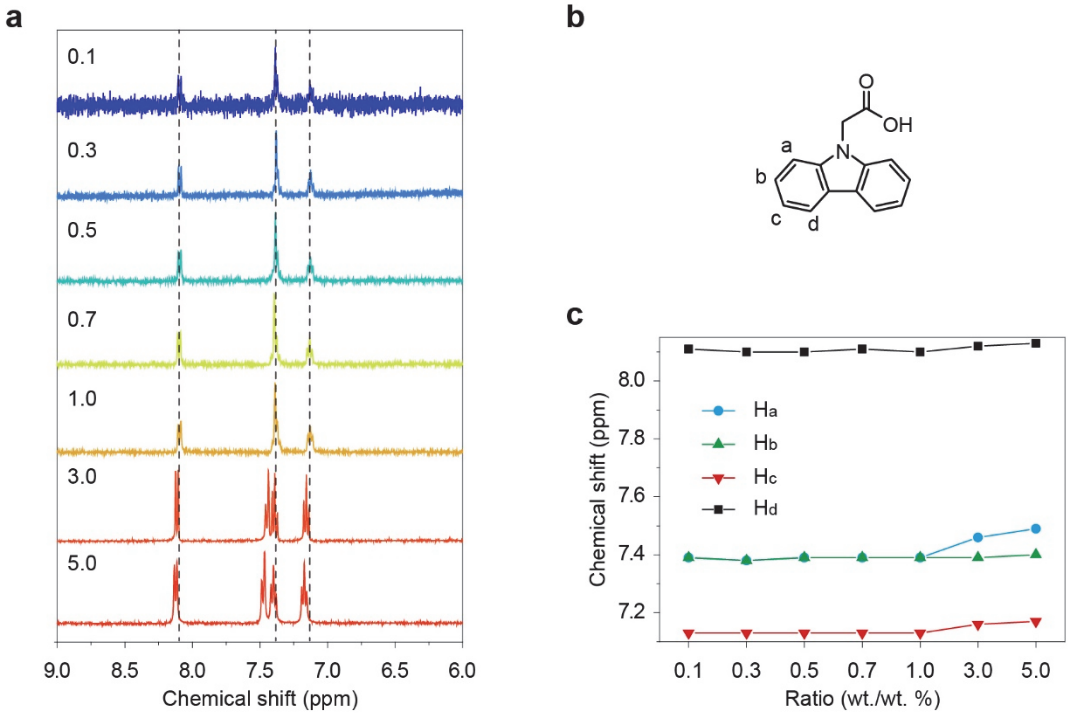

Supplementary Fig. 50 | Chemical shift of aromatic hydrogen of emitters changed with the guest doping ratios. a, Partial ^{1}H NMR spectra of different doped ratios for CzA/PVA films in DMSO-*d*$_6$. **b**, Chemical structure of the CzA molecule. **c**, The chemical shifts of aromatic hydrogens at various doped ratios. Note that the mass concentrations of CzA in PVA matrix are 0.1 wt.%, 0.3 wt.%, 0.5 wt.%, 0.7 wt.%, 1.0 wt.%, 3.0 wt.% and 5.0 wt.%, respectively.

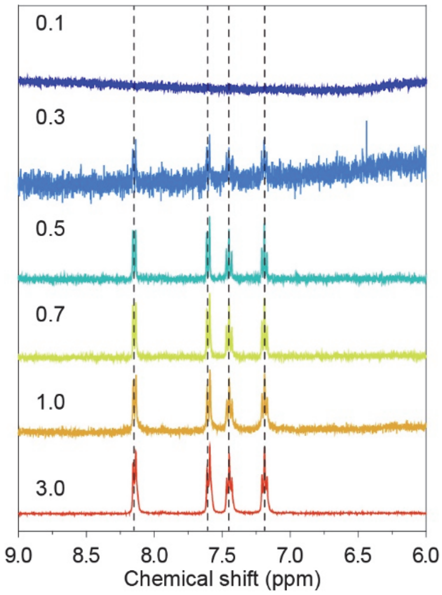

Supplementary Fig. 51 | Partial ^{1}H NMR spectra of EtCz/PVA films with different doped ratios in DMSO-*d*6. Note that the doped ratios of the EtCz molecule are 0.1 wt.%, 0.3 wt.%, 0.5 wt.%, 0.7 wt.%, 1.0 wt.% and 3.0 wt.%, respectively.

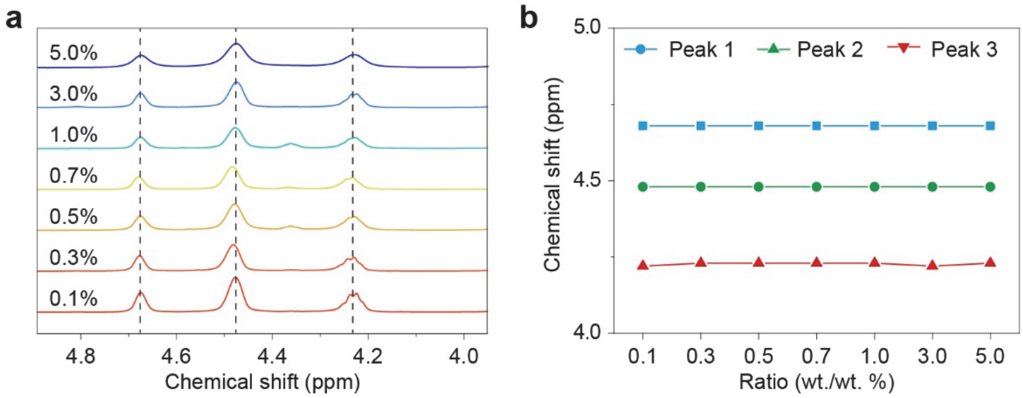

Supplementary Fig. 52 | Chemical shift of hydroxyl hydrogen for the PVA films under different doped ratios. **a**, Partial ^{1}H NMR spectra of CzA/PVA films with different doped ratios in DMSO-*d*6. **b**, The chemical shift value the CzA/PVA films with different doped ratios (appears as a triplet at 4.25 to 4.69 ppm).

The ^{1}H NMR spectra of the CzA/PVA (1.0 wt.%) films with different weight (10, 20, 30 mg) in DMSO-*d*6 (0.5 mL) were collected (Supplementary Fig. 49). It was found that there was no change of the chemical shift for the aromatic chromophores with varying the concentration. Therefore, we concluded that the chemical shift of aromatic hydrogen was independent on the concentration of the sample.

In the further sets of experiments, we collected the ^{1}H NMR spectra of the samples with different doping ratios of PVA and chromophores (CzA and EtCz) in a constant concentration (30 mg film in 0.5 mL DMSO-*d*6). As shown in Supplementary Fig. 50, the chemical shift for aromatic hydrogens of CzA kept identical as the doped ratio varied from 0.1 wt.% to 1.0 wt.%. When the doped ratio of CzA molecule is larger than 1.0 wt.%, we found that the chemical shifts move toward a lower filed. However, compared with the CzA molecule, the chemical shifts of the aromatic

hydrogens of CzA in PVA still move to higher field. For EtCz molecules, the variation of doped ratio do not change the chemical shift of aromatic hydrogens (Supplementary Fig. 51).

The –COOH proton of the chromophores cannot be detected after doping into PVA, which is a common phenomenon during NMR testing. As the doped ratios of CzA variation, there is no change for the chemical shifts of hydroxyl in PVA, which is ascribed to the trace doping of the chromophores (Supplementary Fig. 52).

Taken these results together, we concluded there indeed existed chemical shift variation in the ^{1}H NMR spectra for CzA/PVA.

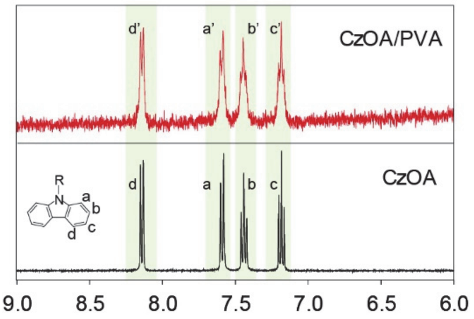

Supplementary Fig. 53 | ^{1}H NMR spectra the CzOA within/without PVA matrix in DMSO-d_6. The concentration of CzOA in PVA film is 0.5 wt.%.

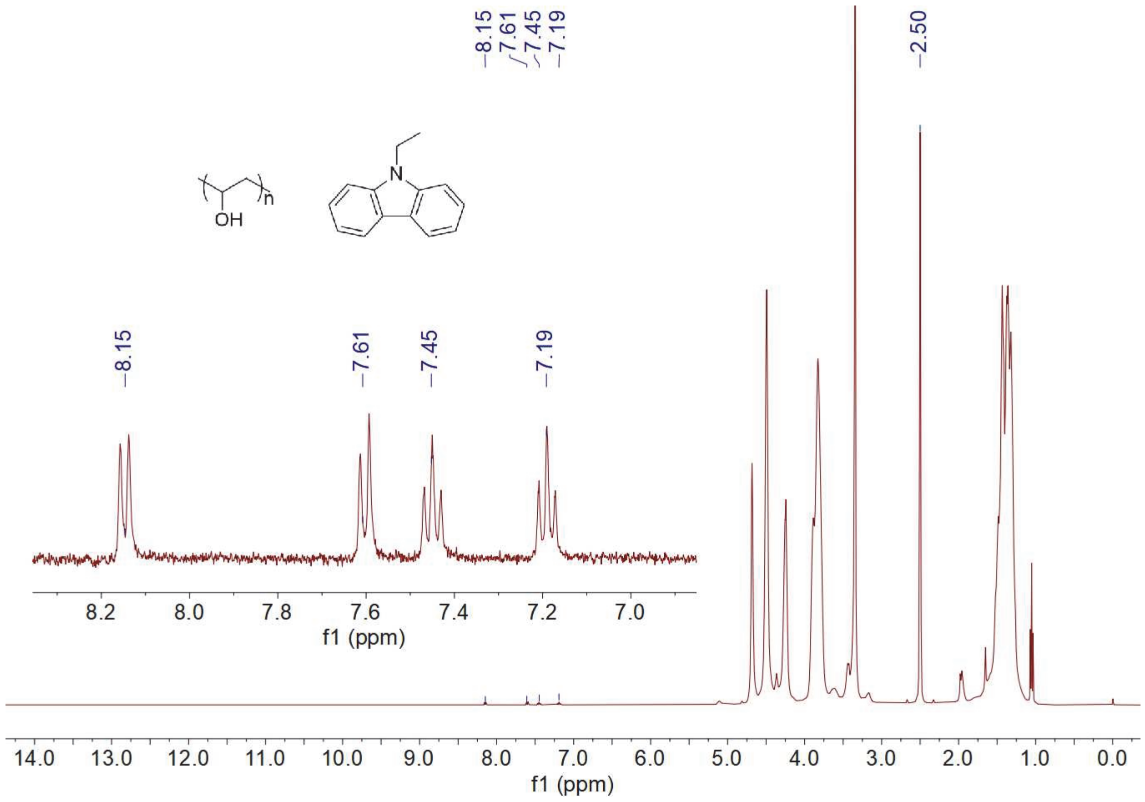

Supplementary Fig. 54 | ^{1}H NMR spectrum of doped film in DMSO-d_6. The concentration of EtCz in PVA matrix is 0.5 wt.%.

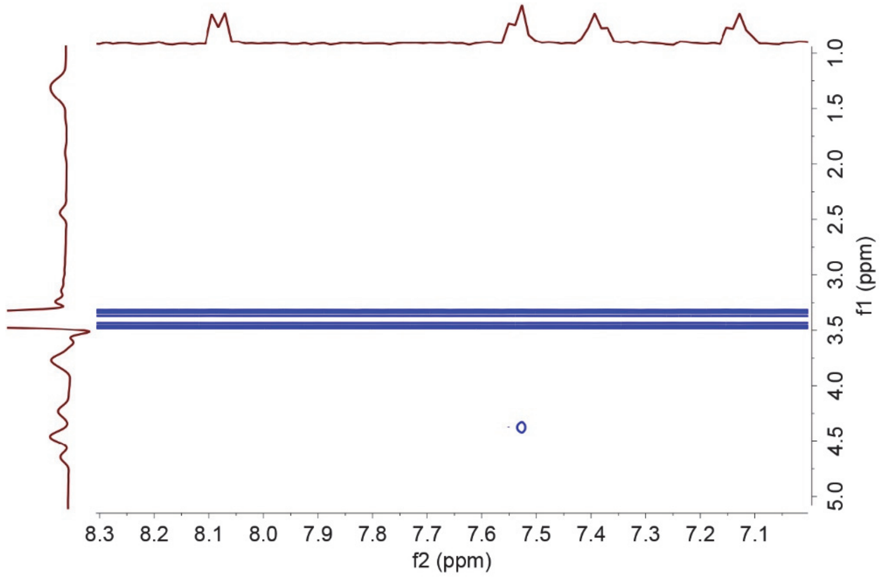

Supplementary Fig. 55 | NOESY NMR spectrum of EtCz in DMSO-*d*$_6$. The doped ratio of EtCz in PVA matrix is 0.5 wt.%.

From the NOESY NMR spectrum shown in Supplementary Fig. 55, it is easily found that these is no relationship of protons between the aromatic region of EtCz and PVA matrix, proving the absence of intermolecular interactions between EtCz and PVA.

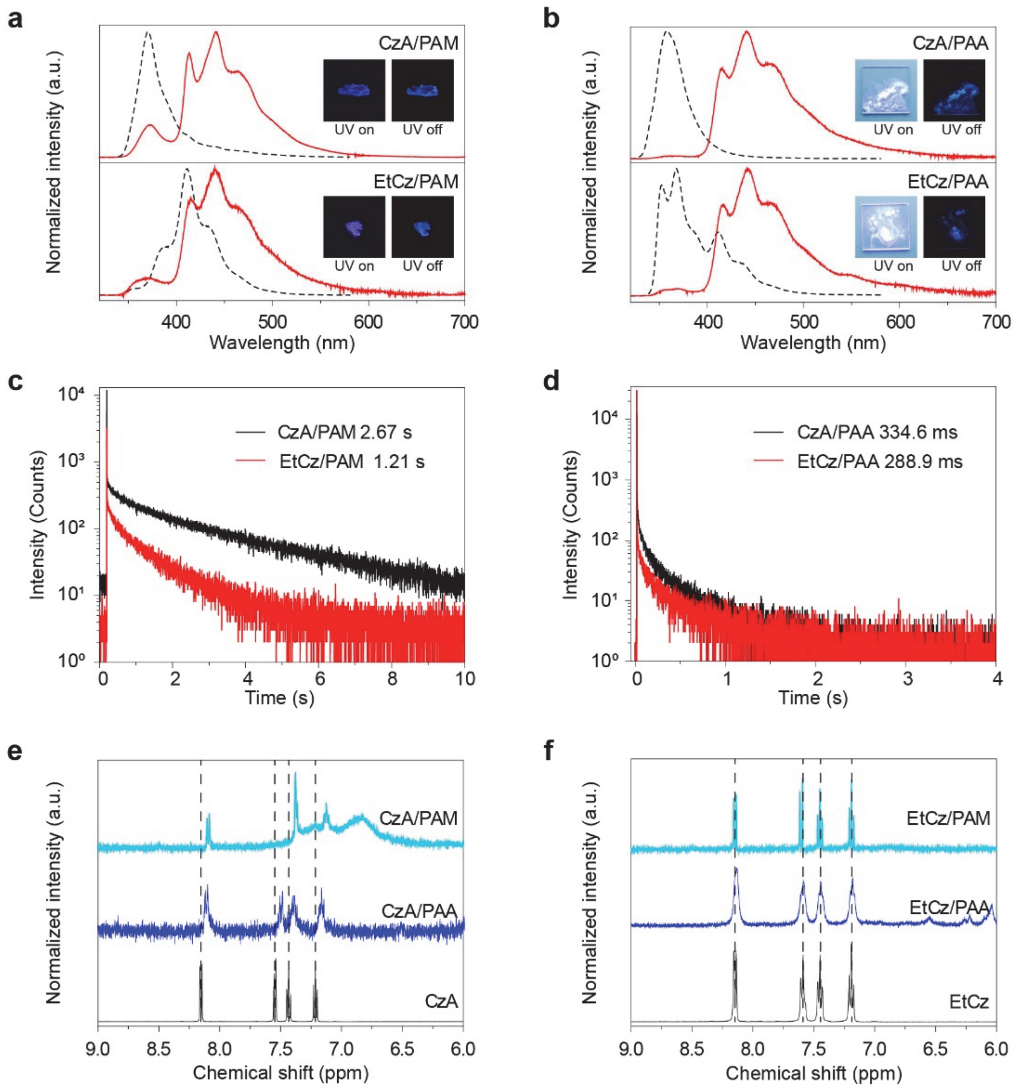

Supplementary Fig. 56 | Photophysical properties and ^{1}H NMR spectra of CzA and EtCz dopant in the PAM and PAA matrices. The normalized steady-state photoluminescence (dash lines) and phosphorescence (solid lines) spectra of CzA (top) and EtCz (bottom) in PAM (**a**) and PAA (**b**) matrix at 1.0 wt.% under ambient conditions. Inset: The left is photographs taken under 302 nm excitation, while the right is photographs of UOP after the removal of ultraviolet source. Lifetime decay profiles of CzA and EtCz in PAM (**c**) and PAA (**d**) matrix monitoring bands at 444 nm at doped ratio 1.0 wt. % under ambient conditions. Note that the excitation wavelength is 300 nm. The ^{1}H NMR spectra of CzA (**e**) and EtCz (**f**) with and without polymer matrix (DMSO-d_6, 293 K).

Supplementary Table 6. Phosphorescence lifetimes of CzA and EtCz doped into PAM and PAA with ratios 1.0 wt.% under ambient conditions.*

Polymer	Emitter	Wavelength (nm)	τ_1 (ms)	A_1 (%)	τ_2 (ms)	A_2 (%)
PAM	CzA	444	290.65	9.02	2663.07	90.98
	EtCz	444	224.13	18.49	1213.26	81.51
PAA	CzA	444	44.65	28.03	334.63	71.97
	EtCz	444	28.34	22.92	288.93	77.08

*Determined from the fitting function of $I_t = \sum A_i e^{-t/\tau_i}$ according to the phosphorescence decay curves, where A_i is the pre-exponential factor for lifetime τ_i.

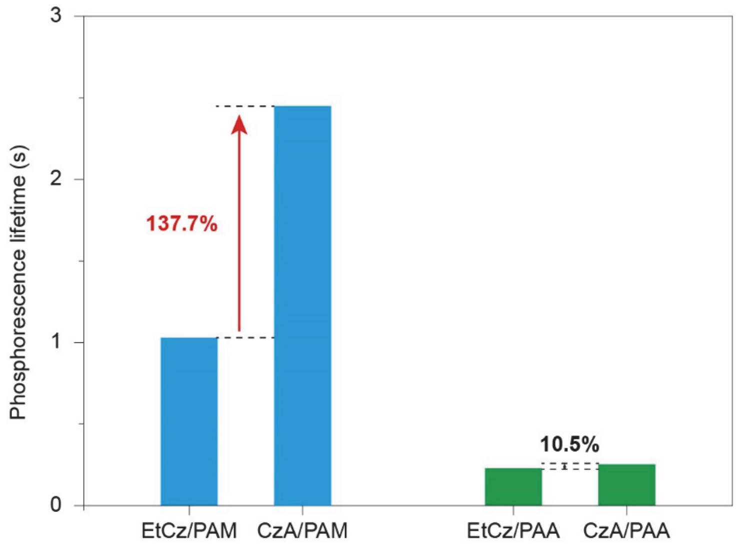

Supplementary Fig. 57 | Comparison of the average phosphorescence lifetime of the films for EtCz and CzA doped in PAM and PAA. The doped ratios of EtCz and CzA doped in PAM and PAA matrix are 1.0 wt.%.

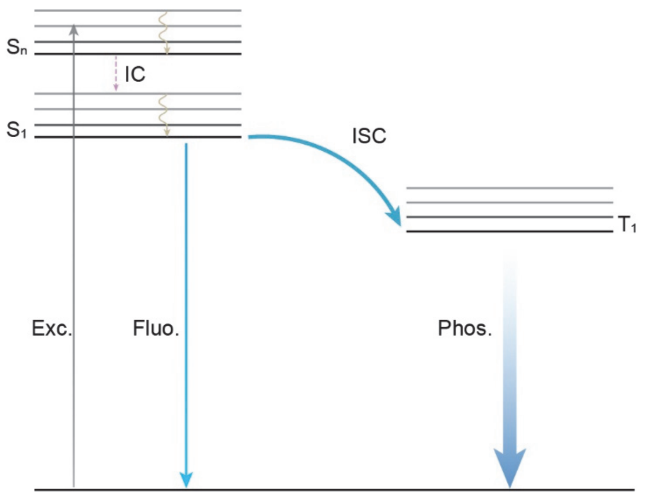

Supplementary Fig. 58 | Proposed energy transfer processes for organic phosphors in PVA film. Note that Exc., IC, ISC, Fluo. and Phos. are the abbreviation of excitation, internal conversion, intersystem crossing, fluorescence and phosphorescence, respectively.

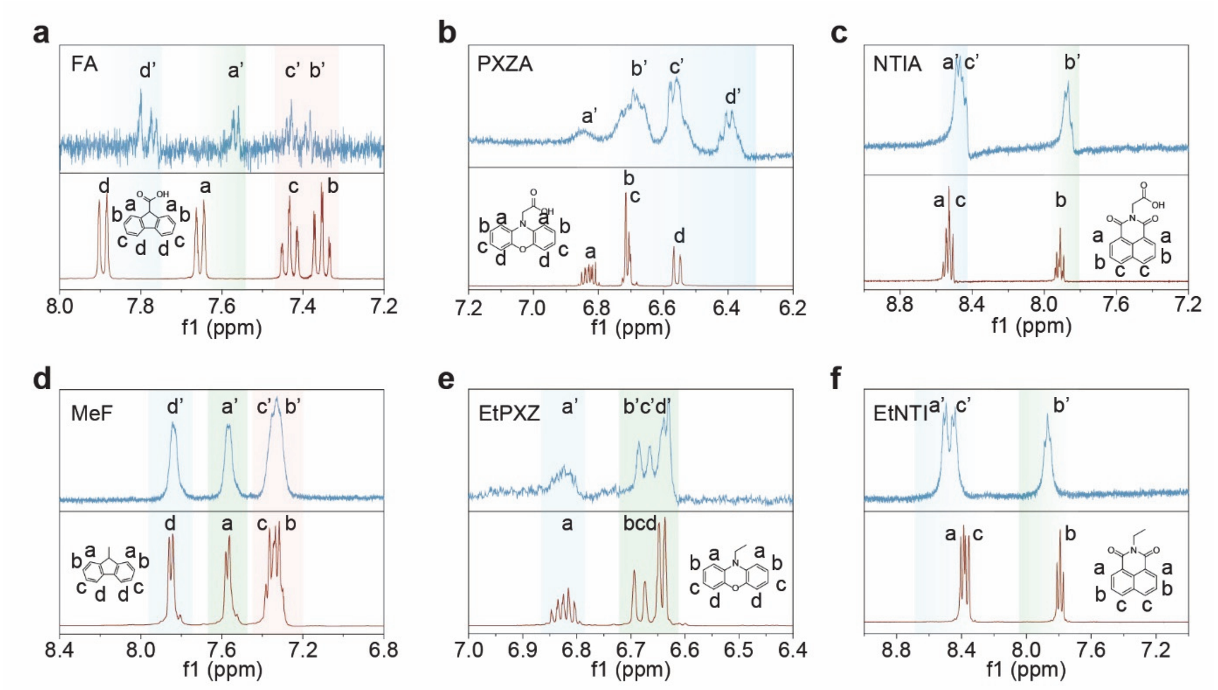

Supplementary Fig. 59 | Comparison of the chemical shifts for emitters with/without PVA matrix in DMSO-*d*$_6$ at 293 K. a, FA. **b**, PXZA. **c**, NTIA. **d**, MeF. **e**, EtPXZ. **f**, EtNTI.

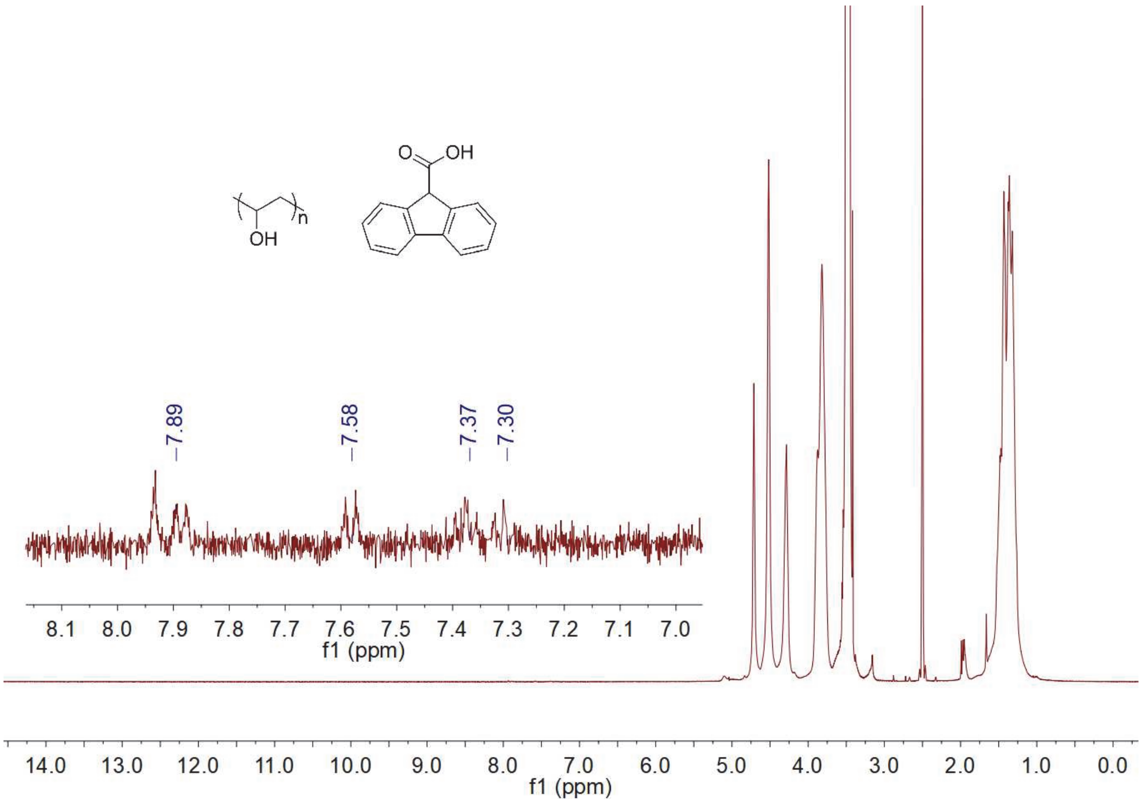

Supplementary Fig. 60 | ^{1}H NMR spectrum of the FA doped film in DMSO-*d*$_6$. The concentration of FA molecule in PVA film is 0.3 wt.%.

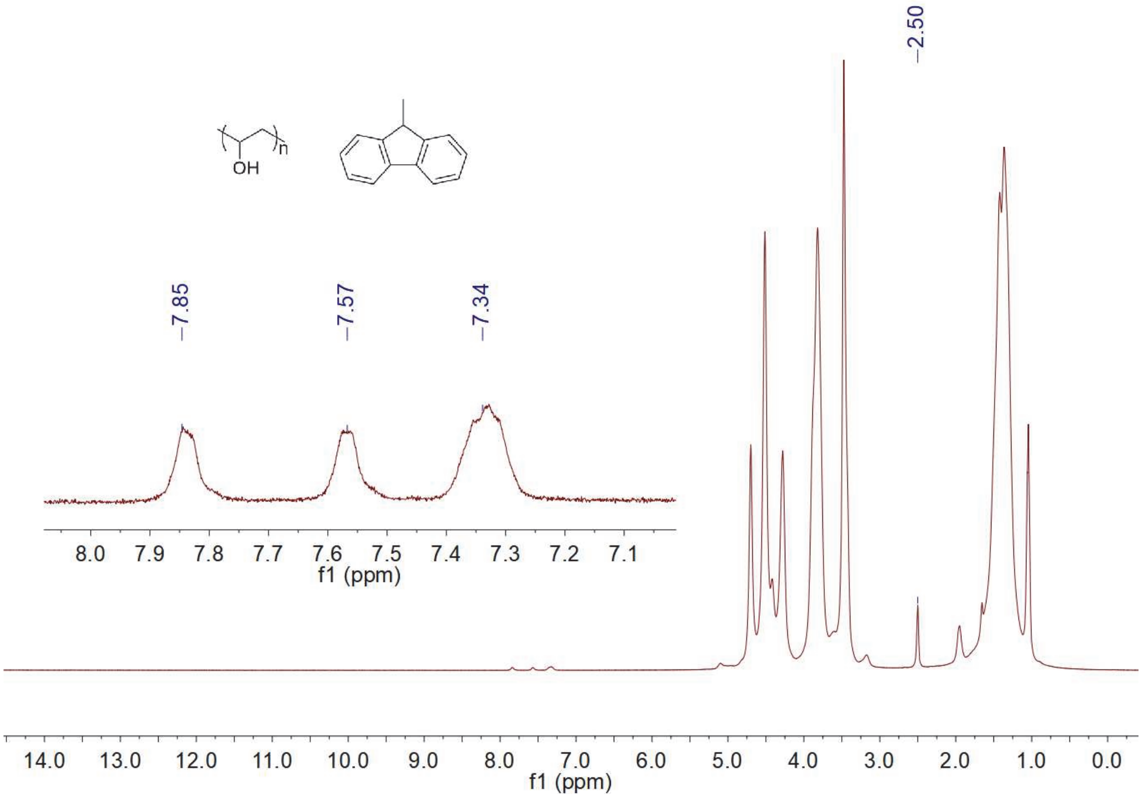

Supplementary Fig. 61 | ^{1}H NMR spectrum of the MeF doped film in DMSO-*d*$_6$. The concentration of MeF molecule in PVA film is 0.3 wt.%.

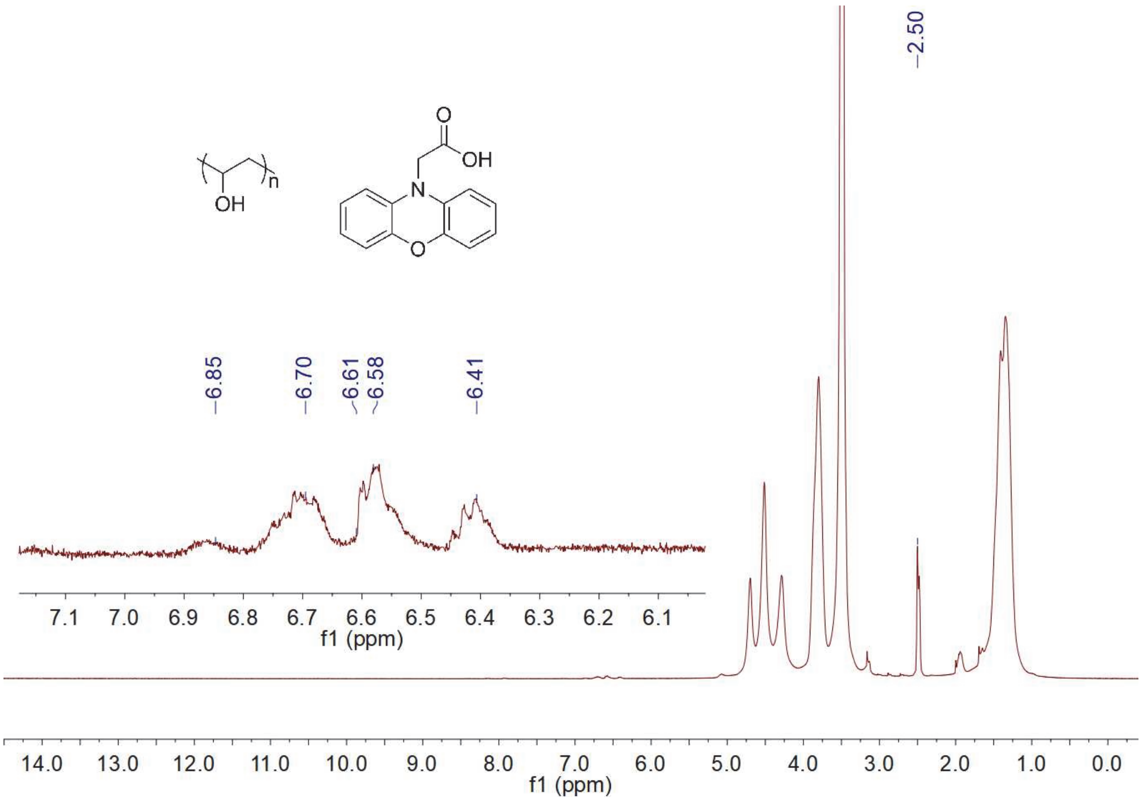

Supplementary Fig. 62 | ^{1}H NMR spectrum of the PXZA doped film in DMSO-*d*$_6$. The concentration of PXZA molecule in PVA film is 1.0 wt.%.

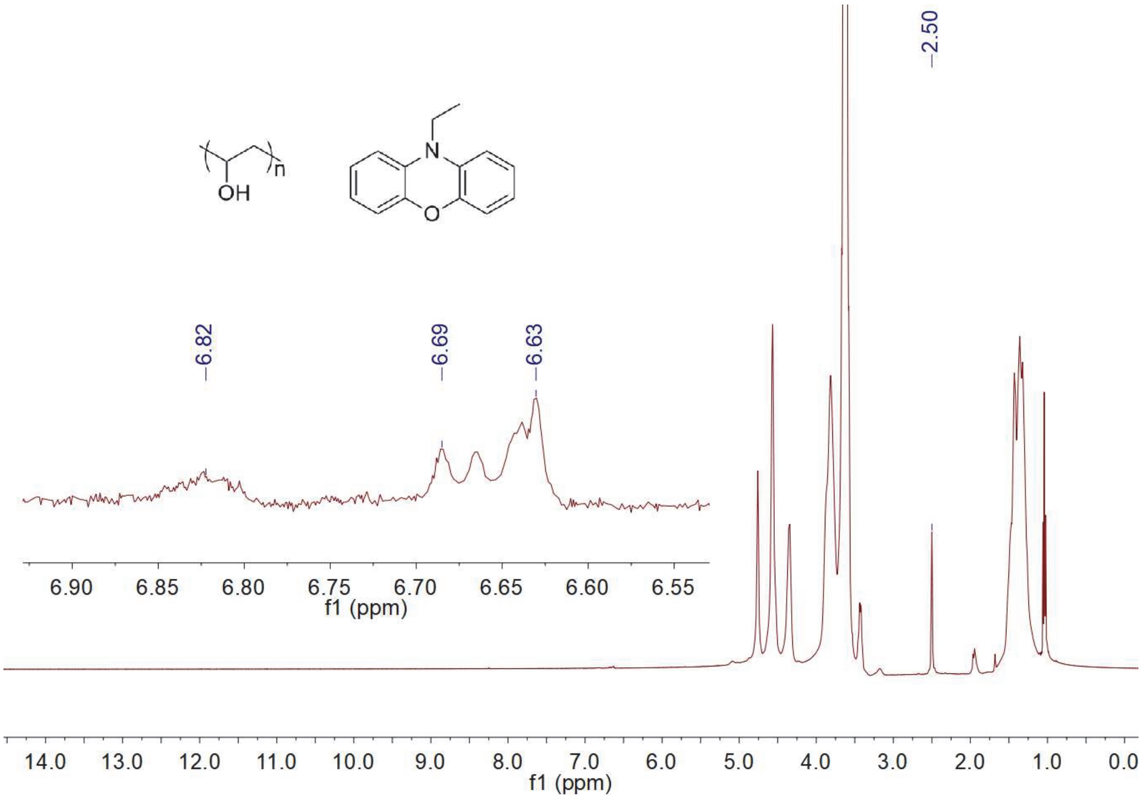

Supplementary Fig. 63 | ^{1}H NMR spectrum of the EtPXZ doped film in DMSO-*d*$_6$. The concentration of EtPXZ molecule in PVA film is 1.0 wt.%.

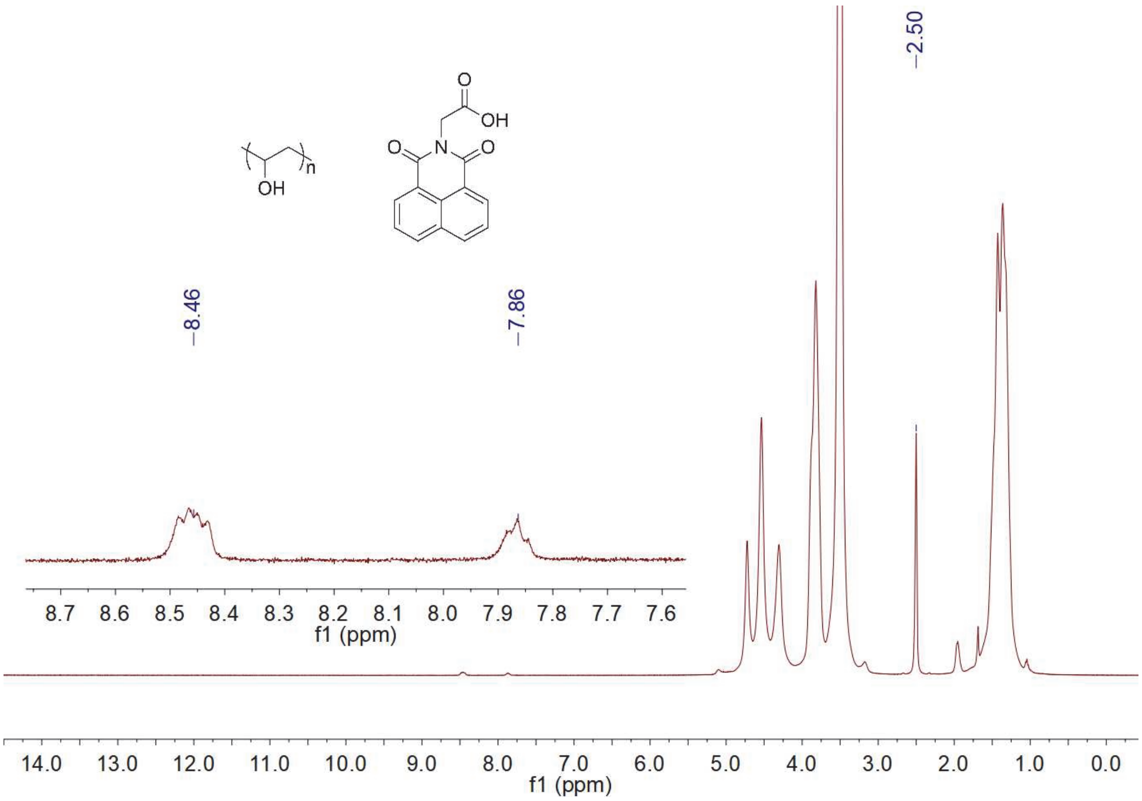

Supplementary Fig. 64 | ^{1}H NMR spectrum of the NTIA doped film in DMSO-*d*$_6$. The concentration of NTIA molecule in PVA film is 0.5 wt.%.

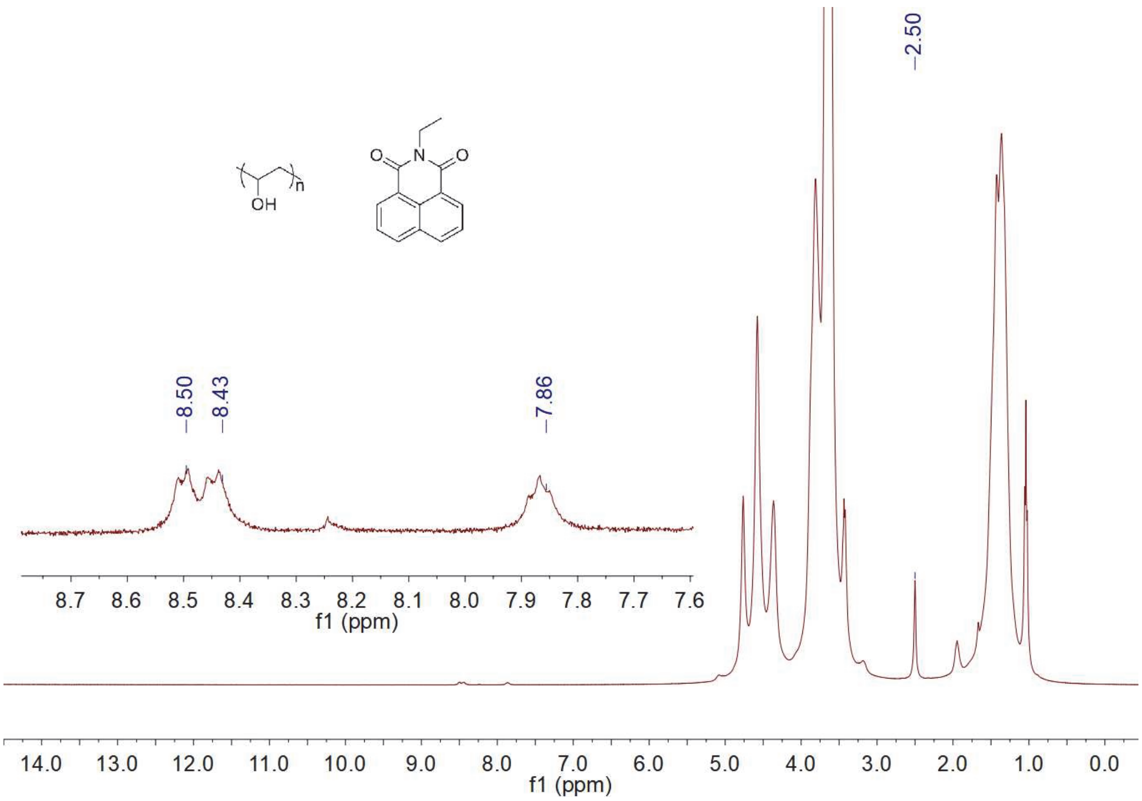

Supplementary Fig. 65 | ^{1}H NMR spectrum of the EtNTI doped film in DMSO-*d*$_6$. The concentration of EtNTI molecule in PVA film is 0.5 wt.%.

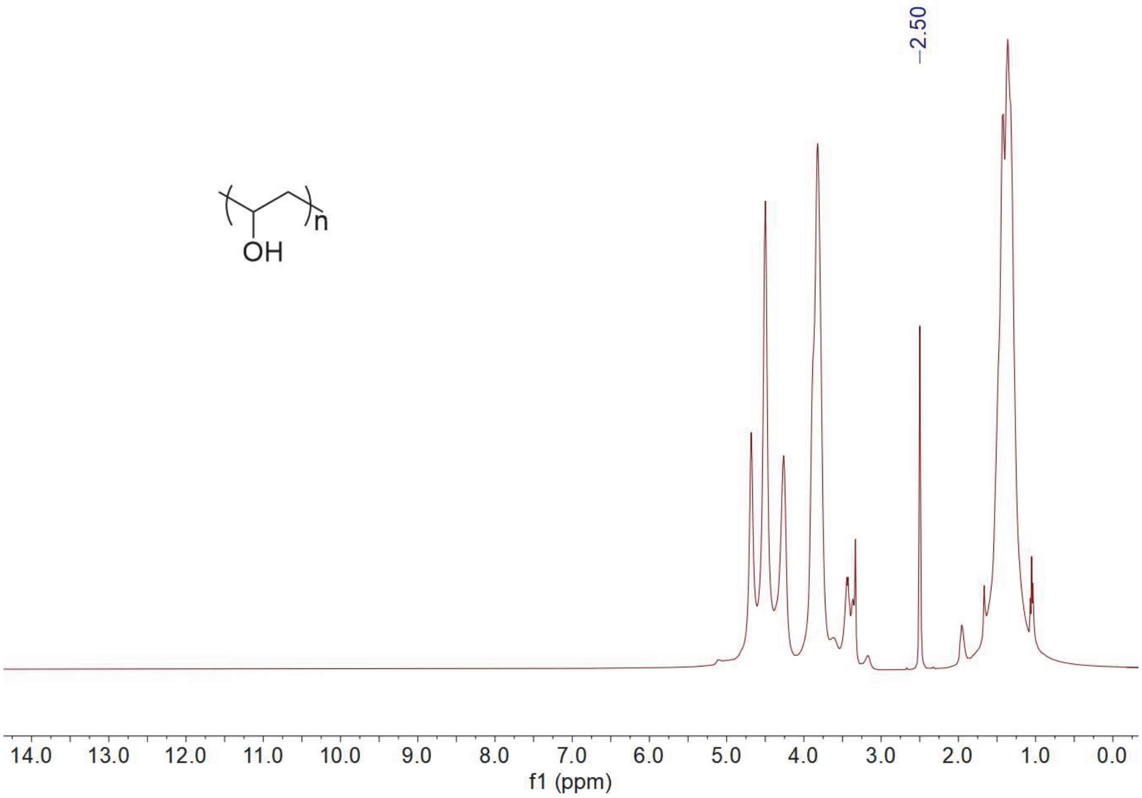

Supplementary Fig. 66 | ^{1}H NMR spectrum of PVA film in DMSO-*d*$_6$. Note that the neat PVA matrix (30 mg) was dissolved in DMSO-*d*$_6$ (0.5 mL).

V. Expand experimental photophysical properties of emitters in film

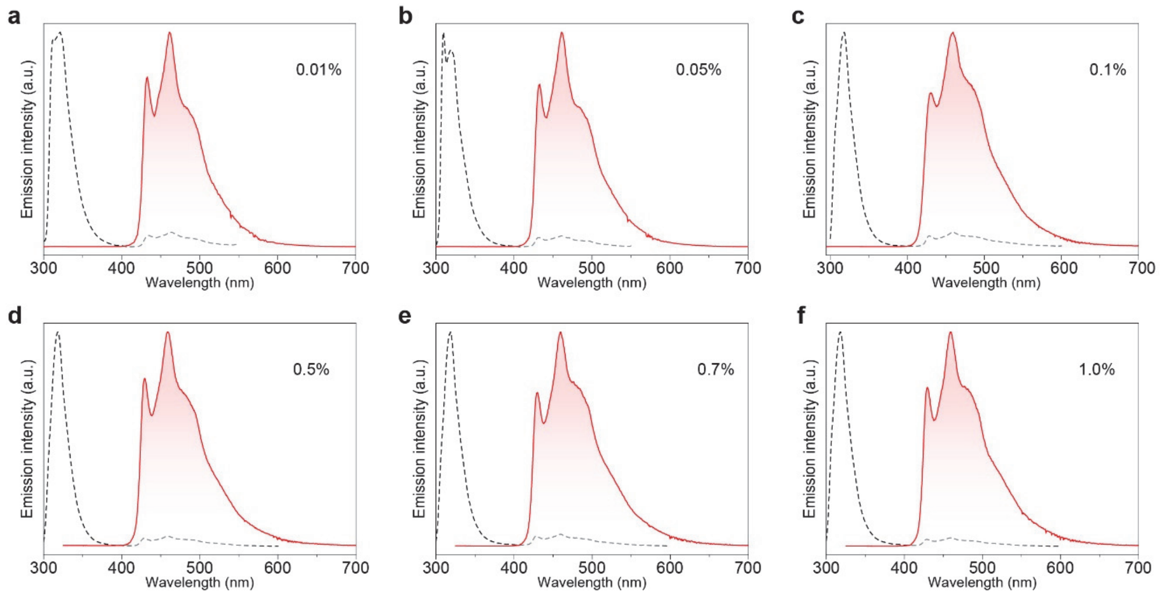

Supplementary Fig. 67 | Normalized steady-state photoluminescence (black dash lines) and phosphorescence (red lines) spectra of FA in films at various doped ratios (wt./wt.) under ambient conditions. **a**, 0.01 wt.%. **b**, 0.05 wt.%. **c**, 0.1 wt.%. **d**, 0.5 wt.%. **e**, 0.7 wt.%. **f**, 1.0 wt.%.

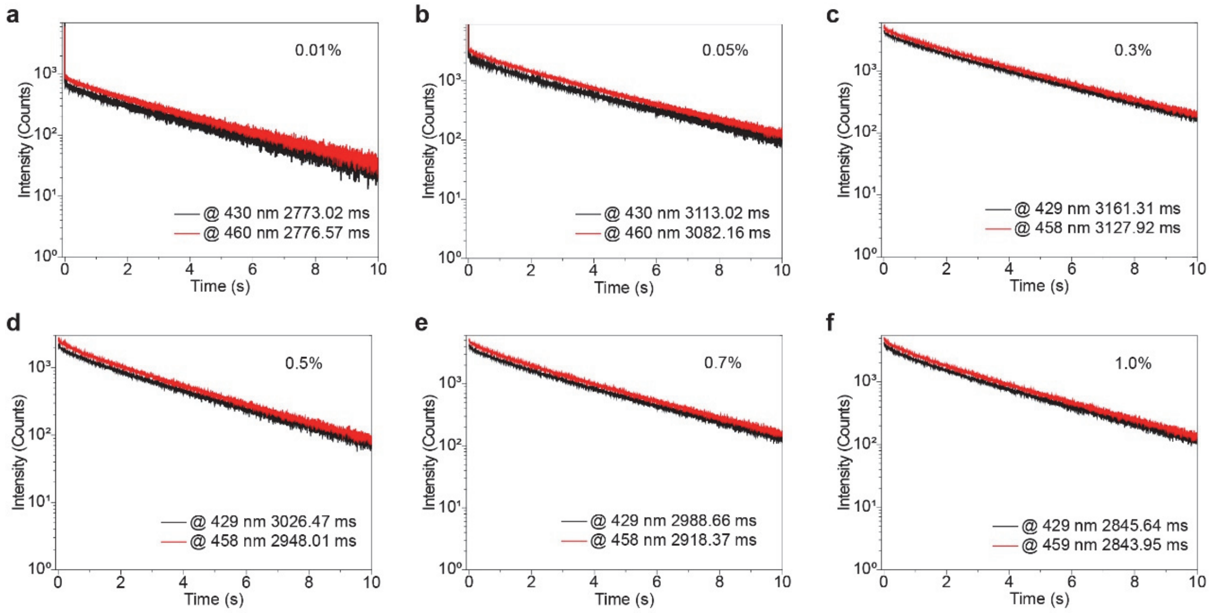

Supplementary Fig. 68 | Lifetime decay profiles of long-lived emission for FA doping PVA films at various doped ratios (wt./wt.) upon excitation at 300 nm UV light under ambient conditions. **a**, 0.01 wt.%. **b**, 0.05 wt.%. **c**, 0.1 wt.%. **d**, 0.5 wt.%. **e**, 0.7 wt.%. **f**, 1.0 wt.%.

Supplementary Table 7. Phosphorescence lifetimes of FA doping PVA films at various ratios under ambient conditions.*

Films	Ratio (%)	Wavelength (nm)	τ_1 (ms)	A_1 (%)	τ_2 (ms)	A_2 (%)
FA/PVA	0.01	430	2773.02	100.00	–	–
		459	2776.57	100.00	–	–
	0.05	430	611.32	4.30	3113.02	95.70
		459	618.77	4.53	3082.16	95.47
	0.1	430	661.48	3.74	3208.30	96.26
		458	597.24	3.44	3162.14	96.56
	0.3	429	622.87	3.60	3161.31	96.40
		458	580.56	3.69	3127.92	96.31
	0.5	429	586.44	4.45	3026.47	95.55
		459	513.29	3.99	2948.01	96.01
	0.7	430	617.53	5.02	2988.66	94.98
		459	555.32	4.82	2918.37	95.18
	1.0	429	521.51	5.02	2845.64	94.98
		459	522.02	5.49	2843.95	94.51

*Determined from the fitting function of $I_t = \sum A_i e^{-t/\tau_i}$ according to the phosphorescence decay curves, where A_i is the pre-exponential factor for lifetime τ_i.

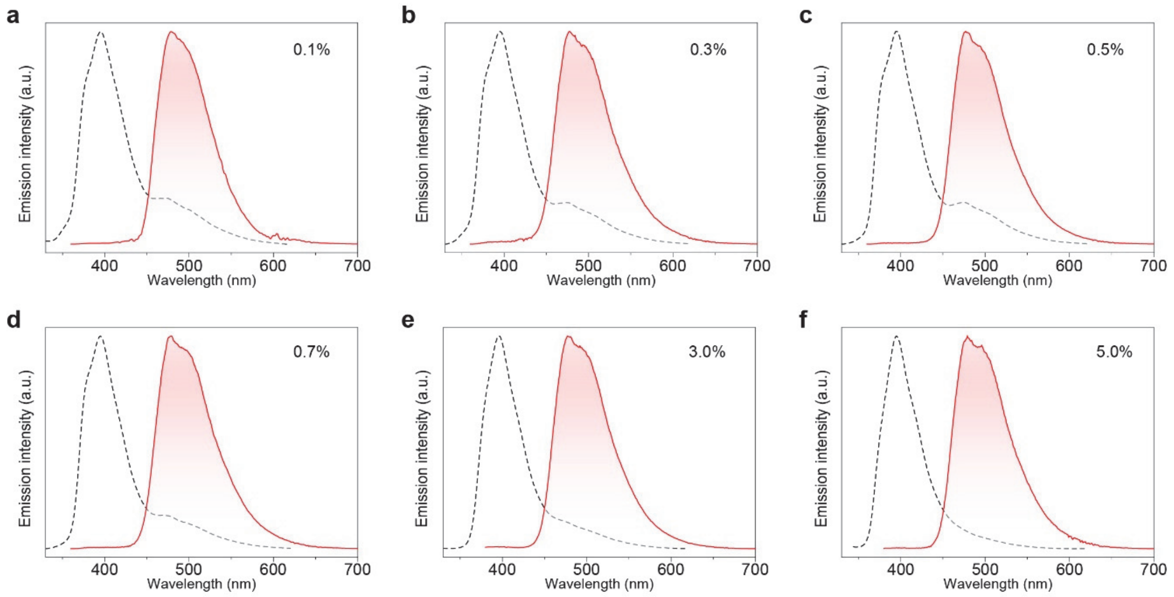

Supplementary Fig. 69 | Normalized steady-state photoluminescence (black dash lines) and phosphorescence (red lines) spectra of PXZA in PVA films at various doped ratios (wt./wt.) upon excitation wavelength at 320 nm under ambient conditions. **a**, 0.1 wt.%. **b**, 0.3 wt.%. **c**, 0.5 wt.%. **d**, 0.7 wt.%. **e**, 3.0 wt.%. **f**, 5.0 wt.%.

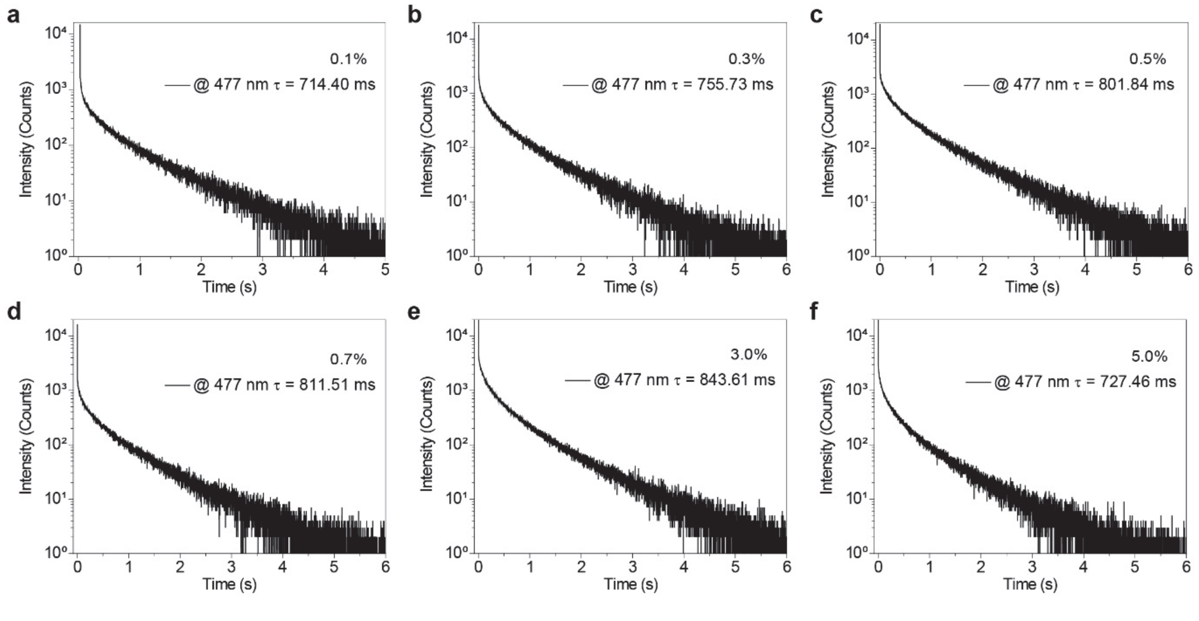

Supplementary Fig. 70 | Lifetime decay profiles of long-lived emission at 477 nm for PXZA in PVA films at various doped ratios (wt./wt.) under ambient conditions. The excitation wavelength is 320 nm. **a**, 0.1 wt.%. **b**, 0.3 wt.%. **c**, 0.5 wt.%. **d**, 0.7 wt.%. **e**, 3.0 wt.%. **f**, 5.0 wt.%.

Supplementary Table 8. Phosphorescence lifetimes of PXZA in PVA films at various ratios under ambient conditions.*

Films	Ratio (%)	Wavelength (nm)	τ_1 (ms)	A_1 (%)	τ_2 (ms)	A_2 (%)	τ_3 (ms)	A_3 (%)
PXZA/PVA	0.1	477	113.70	24.54	714.40	75.46	–	–
	0.3	477	124.66	25.83	755.73	74.17	–	–
	0.5	477	156.55	27.25	801.84	72.75	–	–
	0.7	477	29.81	5.76	212.72	29.51	811.51	64.73
	1.0	477	33.63	6.26	246.34	33.13	876.89	60.61
	3.0	477	39.80	8.46	243.78	39.09	843.61	2.45
	5.0	477	28.80	9.54	189.12	37.11	727.46	53.34

*Determined from the fitting function of $I_t = \sum A_i e^{-t/\tau_i}$ according to the phosphorescence decay curves, where A_i is the pre-exponential factor for lifetime τ_i.

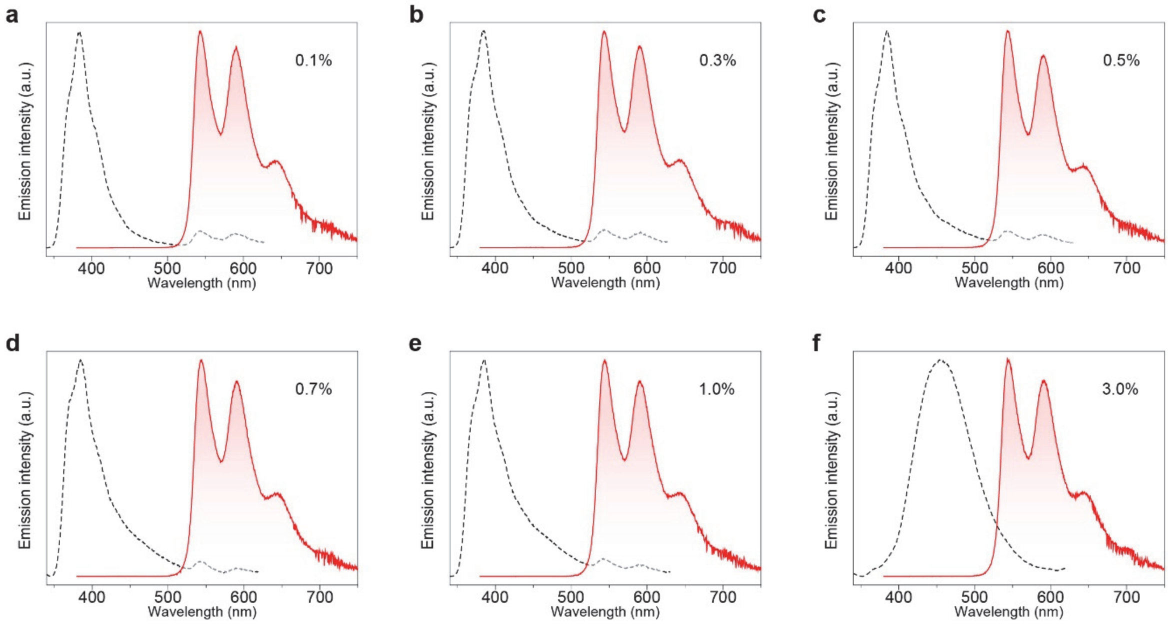

Supplementary Fig. 71 | Normalized steady-state photoluminescence (black dash lines) and phosphorescence (red lines) spectra of NTIA in PVA films at various doped ratios (wt./wt.) under ambient conditions. **a**, 0.1 wt.%. **b**, 0.3 wt.%. **c**, 0.5 wt.%. **d**, 0.7 wt.%. **e**, 1.0 wt.%. **f**, 3.0 wt.%.

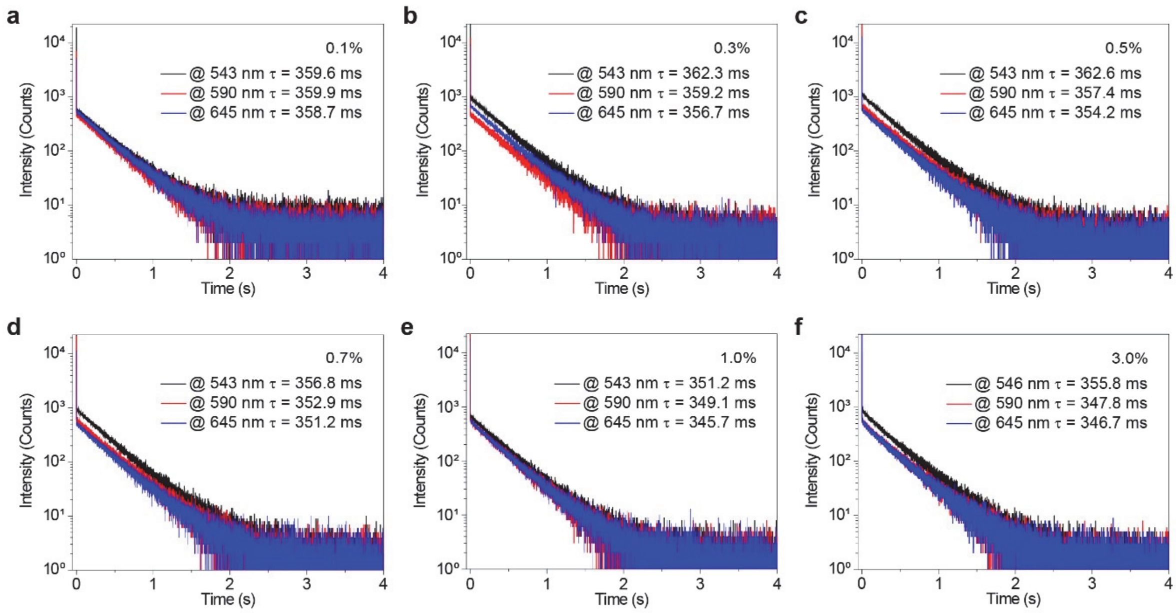

Supplementary Fig. 72 | Lifetime decay profiles of long-lived emission for NTIA in PVA films at different doped ratios (wt./wt.) under ambient conditions. a, 0.1 wt.%. **b**, 0.3 wt.%. **c**, 0.5 wt.%. **d**, 0.7 wt.%. **e**, 1.0 wt.%. **f**, 3.0 wt.%. The excitation wavelength is 340 nm.

Supplementary Table 9. Phosphorescence lifetimes of NTIA in PVA films at various ratios under ambient conditions.*

Films	Ratio (%)	Wavelength (nm)	τ (ms)	A (%)
NTIA/PVA	0.1	543	359.62	100.00
		591	359.87	100.00
		645	358.71	100.00
	0.3	543	362.25	100.00
		590	359.21	100.00
		645	356.75	100.00
	0.5	543	362.60	100.00
		590	357.42	100.00
		645	354.20	100.00
	0.7	543	356.81	100.00
		590	352.90	100.00
		645	351.22	100.00
	1.0	543	351.25	100.00
		590	349.13	100.00
		645	345.73	100.00
	3.0	543	355.83	100.00
		590	347.83	100.00
		645	346.40	100.00

*Determined from the fitting function of $I_t = \sum A_i e^{-t/\tau_i}$ according to the phosphorescence decay curves, where A_i is the pre-exponential factor for lifetime τ_i.

Supplementary Table 10. Phosphorescence lifetimes of CzA, FA, PXZA, NTIA, and EtCz in *m*-THF (1 × 10^{-5} M) at 77 K and in PVA matrix under ambient conditions.

	CzA	FA	PXZA	NTIA	EtCz
m-THF (77 K)	5.7 s	4.5 s	2.6 s	508.8 ms	6.3 s
PVA matrix	2.76 s	3.21 s	876.9 ms	362.6 ms	1.75 s
Ratio	48.4%	71.3%	33.7%	71.3%	27.5%

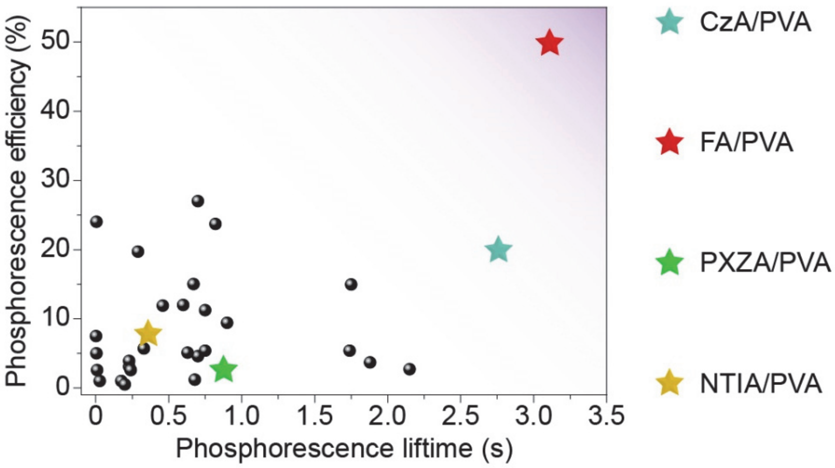

Supplementary Fig. 73 | The chart of phosphorescence lifetimes and efficiencies of base on polymer as a host. The dots and stars represent organic phosphor doped polymers in previous literature and in this work, respectively[4-14].

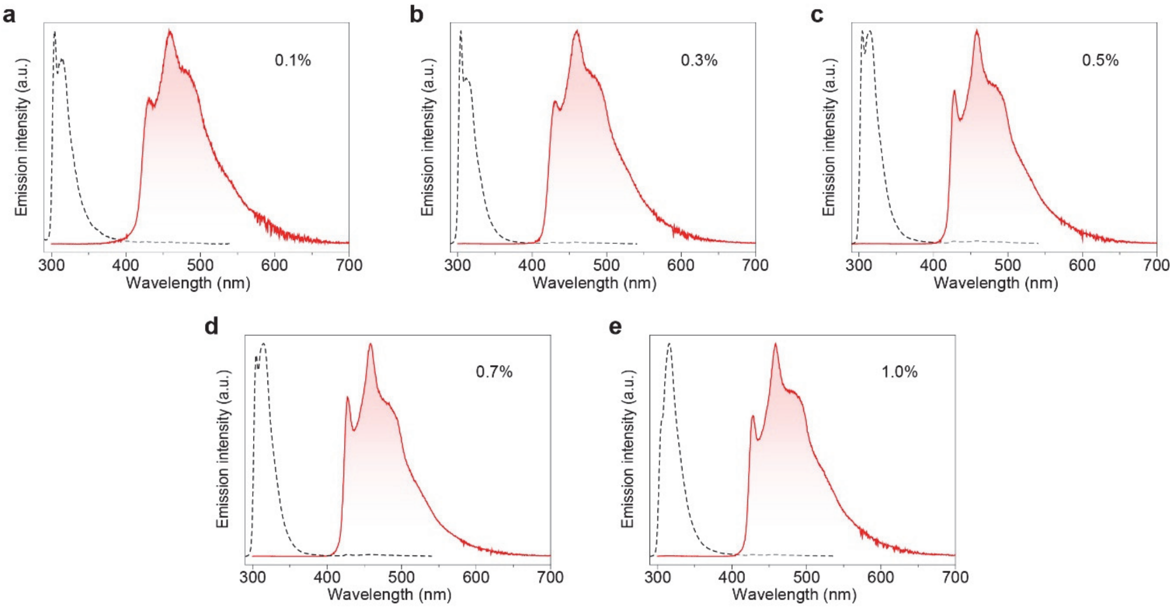

Supplementary Fig. 74 | Normalized steady-state photoluminescence (black dash lines) and phosphorescence (red lines) spectra of MeF in film at various doped ratios (wt./wt.) under ambient conditions. a, 0.1 wt.%. **b**, 0.3 wt.%. **c**, 0.5 wt.%. **d**, 0.7 wt.%. **e**, 1.0 wt.%.

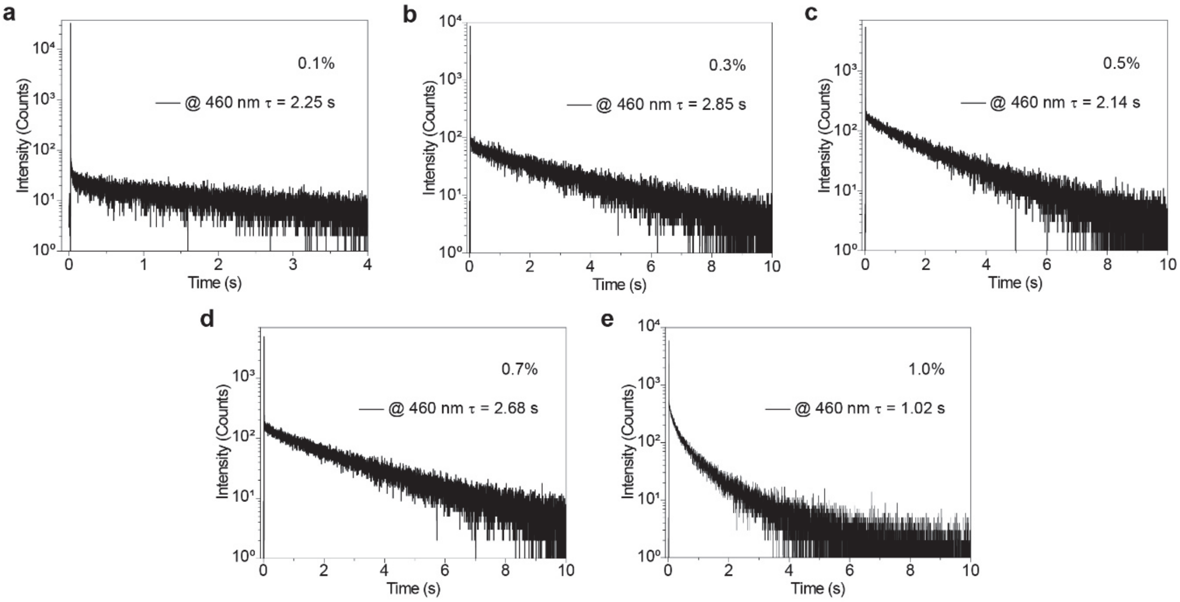

Supplementary Fig. 75 | Lifetime decay profiles of UOP band around 460 nm for MeF in film at various doped ratio (wt./wt.) upon excitation by 300 nm UV light under ambient conditions. a, 0.1 wt.%. **b**, 0.3 wt.%. **c**, 0.5 wt.%. **d**, 0.7 wt.%. **e**, 1.0 wt.%.

Supplementary Table 11. Phosphorescence lifetimes of MeF in film at various ratios under ambient conditions.*

Films	Ratio (%)	Wavelength (nm)	τ_1 (ms)	A_1 (%)	τ_2 (ms)	A_2 (%)
	0.1	460	21.29	3.04	2257.99	96.96
	0.3	460	324.52	3.24	2856.90	96.76
MeF/PVA	0.5	460	514.69	9.16	2145.24	90.84
	0.7	460	656.57	7.30	2682.81	92.70
	1.0	460	182.93	32.85	1024.24	67.15

*Determined from the fitting function of $I_t = \sum A_i e^{-t/\tau_i}$ according to the phosphorescence decay curves, where A_i is the pre-exponential factor for lifetime τ_i.

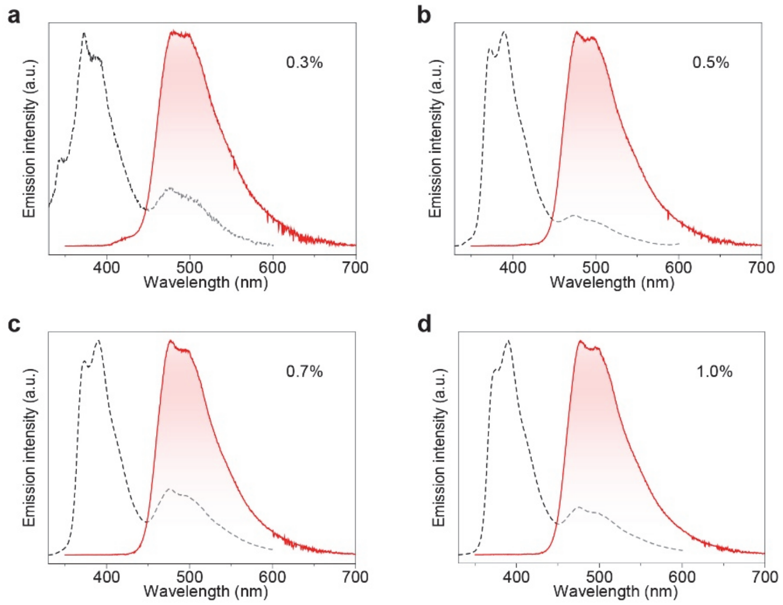

Supplementary Fig. 76 | Normalized steady-state photoluminescence (black dash lines) and phosphorescence (red lines) spectra of EtPXZ in PVA films with various doped ratios (wt./wt.) under ambient conditions. **a**, 0.3 wt.%. **b**, 0.5 wt.%. **c**, 0.7 wt.%. **d**, 1.0 wt.%.

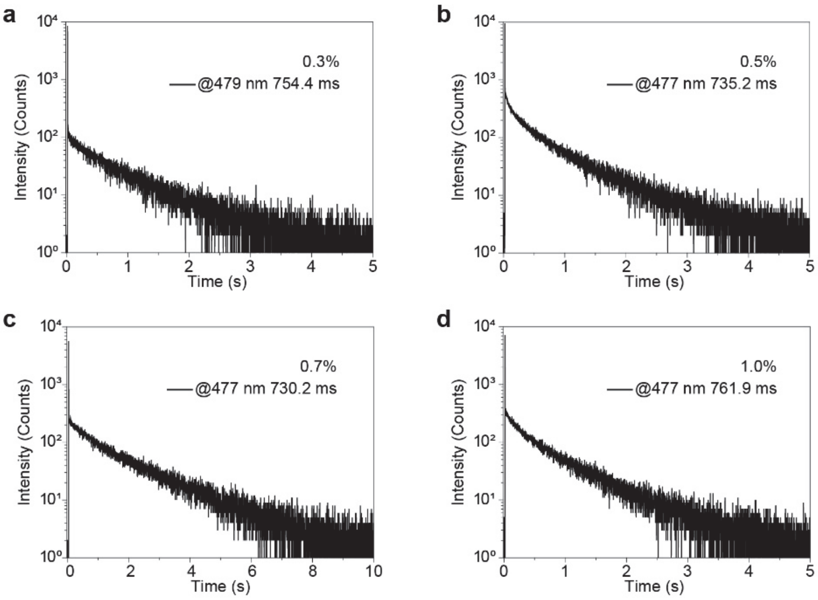

Supplementary Fig. 77 | Lifetime decay profiles of long-lived emission for EtPXZ chromophores in PVA films with various doped ratios (wt./wt.) under ambient conditions. a, 0.3 wt.%. **b**, 0.5 wt.%. **c**, 0.7 wt.%. **d**, 1.0 wt.%. The excitation wavelength is 340 nm.

Supplementary Table 12. Phosphorescence lifetimes of EtPXZ doped PVA films with various ratios under ambient conditions.*

Films	Ratio (%)	Wavelength (nm)	τ_1 (ms)	A_1 (%)	τ_2 (ms)	A_2 (%)
EtPXZ/PVA	0.3	479	104.44	6.98	754.45	93.02
	0.5	477	121.37	20.70	735.25	79.30
	0.7	477	730.22	100.00	–	–
	1.0	477	160.12	17.41	761.95	82.59

*Determined from the fitting function of $I_t = \sum A_i e^{-t/\tau_i}$ according to the phosphorescence decay curves, where A_i is the pre-exponential factor for lifetime τ_i.

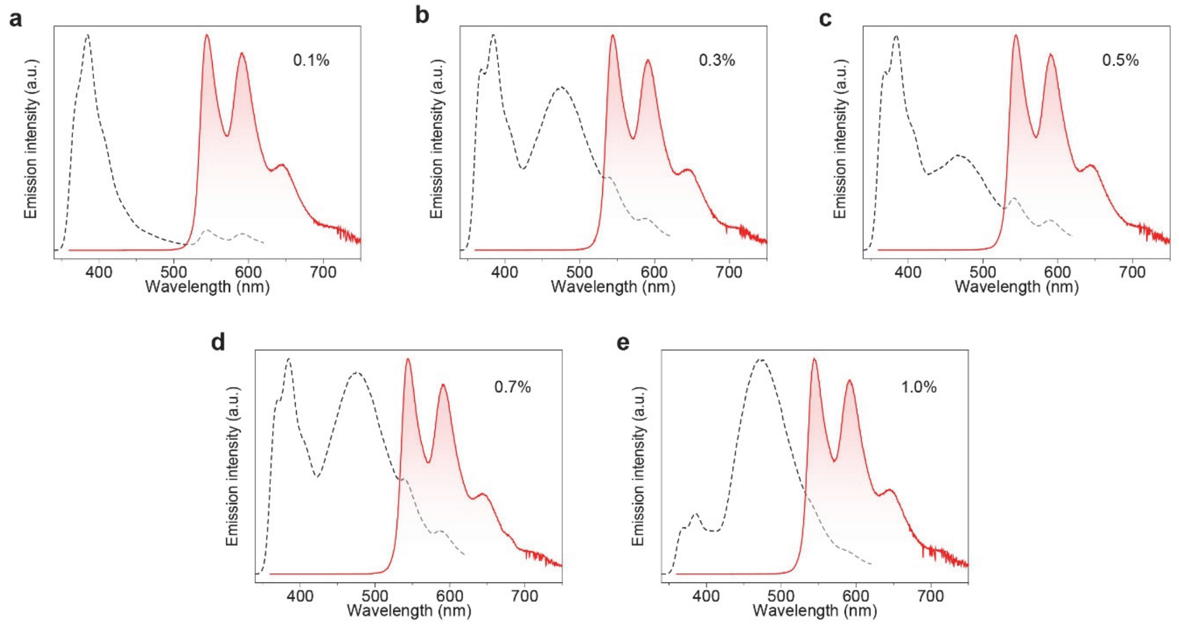

Supplementary Fig. 78 | Normalized steady-state photoluminescence (black dash lines) and phosphorescence (red lines) spectra of EtNTI-doped PVA films with different ratios (wt./wt.) under ambient conditions. a, 0.1 wt.%. **b**, 0.3 wt.%. **c**, 0.5 wt.%. **d**, 0.7 wt.%. **e**, 1.0 wt.%.

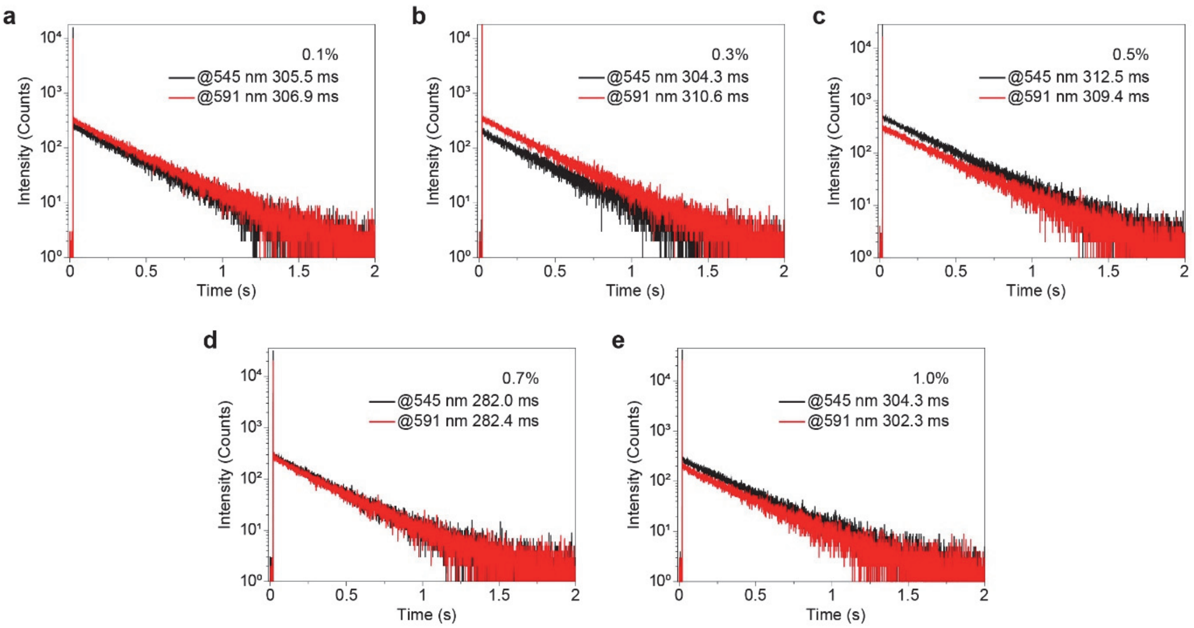

Supplementary Fig. 79 | Lifetime decay profiles of UOP bands around 545 and 591 nm for EtNTI-doped PVA films with different ratios (wt./wt.) upon excitation of 360 nm UV light under ambient conditions. a, 0.1 wt.%. **b**, 0.3 wt.%. **c**, 0.5 wt.%. **d**, 0.7 wt.%. **e**, 1.0 wt.%.

Supplementary Table 13. Phosphorescence lifetimes of EtNTI-doped PVA films with different ratios (wt./wt.) under ambient conditions.*

Films	Ratio (%)	Wavelength (nm)	τ (ms)	A (%)
EtNTI/PVA	0.1	545	305.51	100.00
		591	306.98	100.00
	0.3	545	304.32	100.00
		591	310.63	100.00
	0.5	545	312.54	100.00
		591	309.49	100.00
	0.7	545	282.01	100.00
		591	282.46	100.00
	1.0	545	304.49	100.00
		591	302.38	100.00

*Determined from the fitting function of $I_t = \sum A_i e^{-t/\tau_i}$ according to the phosphorescence decay curves, where A_i is the pre-exponential factor for lifetime τ_i.

Supplementary Table 14. Phosphorescence lifetimes of organic phosphor doped PVA films under ambient conditions.

Films	Phosphorescence lifetime (s)
CzA	2.76
EtCz	1.75
FA	3.21
MeF	2.86
PXZA	0.88
EtPXZ	0.76
NTIA	0.36
EtNTI	0.31

To gain deep insight into the influence of the carboxylic group on phosphorescence, a set of control emitters without carboxylic groups were designed and synthesized (**Supplementary Fig. 1**), which are named 9-methyl-9*H*-fluorene (MeF), 10-ethyl-10*H*-phenoxazine (EtPXZ), and 2-ethyl-1*H*-benzo[*de*]isoquinoline-1,3(2*H*)-dione (EtNTI), respectively. After elimination of carboxylic group, the corresponding repulsive interactions between emitters and PVA matrix disappeared (**Supplementary Fig. 59d-59f**), leading to decrease of UOP performance in turn (**Supplementary Fig. 74-79, Supplementary Table 14**).

Supplementary Table 15. Photophysical parameters of organic phosphor doped PVA films under ambient conditions.

Films	τ_F (ns)	τ_P (s)	Φ_F (%)	Φ_P (%)	k_{isc} (s^{-1})[b]	k_P (s^{-1})[c]	k_{nr}(s^{-1})[d]
CzA	10.7	2.73	74.70	20.10	2.36×10^7	0.29	0.08
EtCz	13	1.75	79.90	14.00	1.55×10^7	0.40	0.17
FA	4.9	3.21	49.00	50.00	1.04×10^8	0.31	0.01
PXZA	6.2	0.877	13.30	2.80	1.40×10^8	0.04	1.10
NTIA	7.9	0.363	85.70	8.00	1.81×10^7	1.54	1.21

[a)] $\Phi_{isc} = 1 - \Phi_F - \Phi_{ic} \approx 1 - \Phi_F$; [b)] $k_{isc} = \Phi_{isc}/\tau_F$; [c)] $k_p = \Phi_P/(\Phi_{isc} \times \tau_P)$; [d)] $k_{nr} = 1/\tau_P - k_p$.

VI. Applications

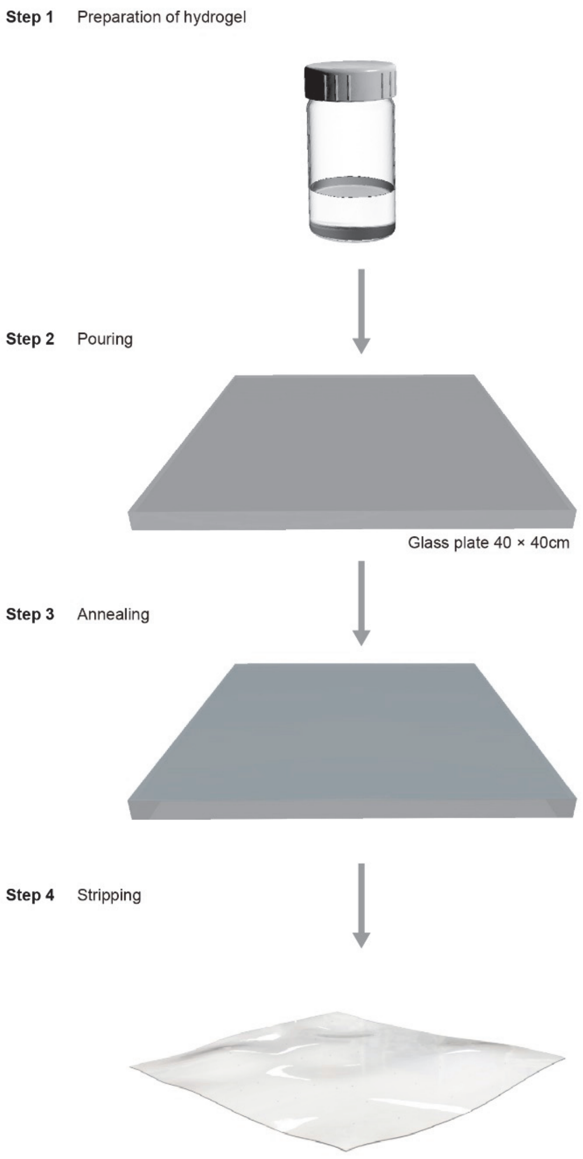

Supplementary Fig. 80 | Preparation process of the large-area transparent films with UOP. The transparent films are fabricated following the process of hydrogel preparation, pouring, annealing and stripping, respectively.

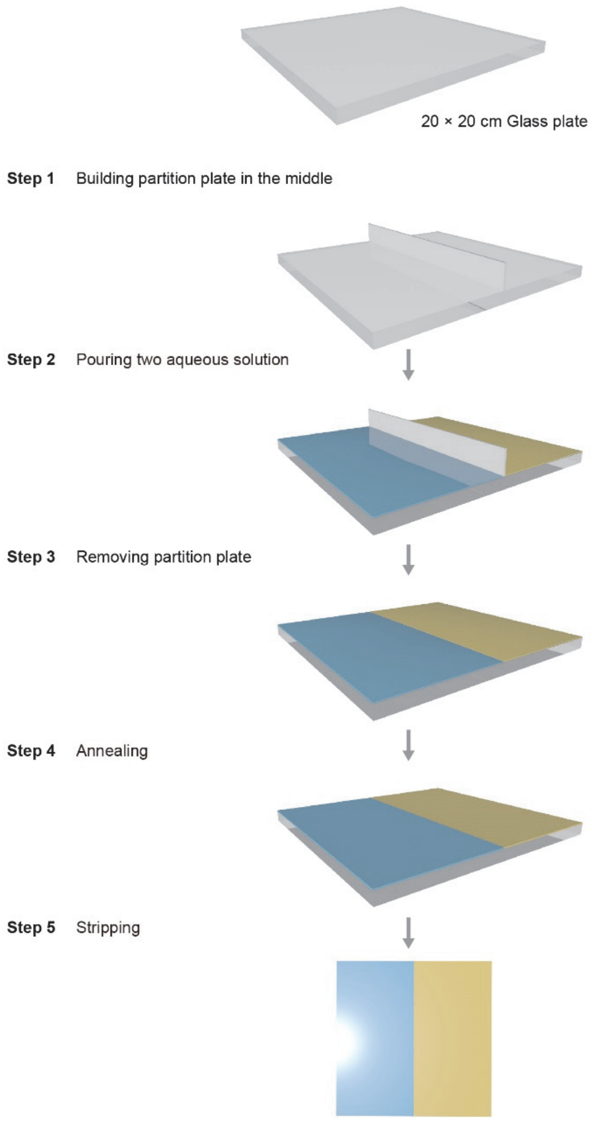

Supplementary Fig. 81 | Process for the preparation of the large-area transparent film with two UOP colors. Firstly, partition plate in the middle of glass was built. Two aqueous solutions were then poured on the glass substrate. After that, the partition plate was removed and the film was annealed at 60ºC for the target films with colorful ultralong phosphorescence feature.

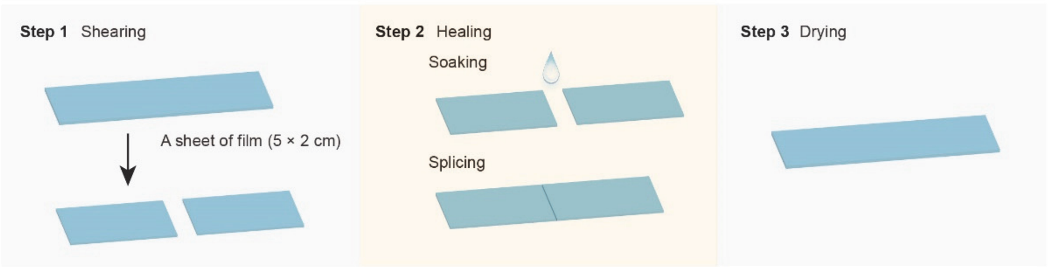

Supplementary Fig. 82 | Demonstration of the self-healing process for PVA films with UOP feature. There exist three steps, including shearing, healing and drying.

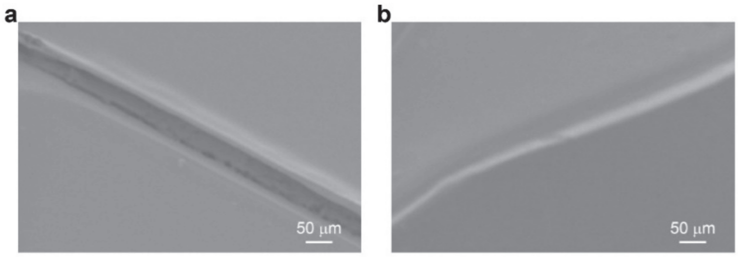

Supplementary Fig. 83 | SEM images of the scribed region of organic phosphor doped PVA film. a, Before self-healing. **b**, After self-healing.

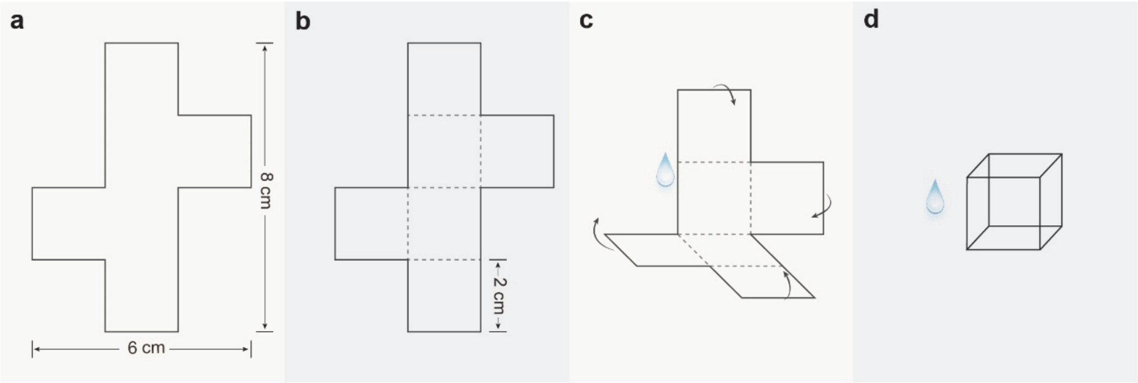

Supplementary Fig. 84 | Schematic diagram of fabricating a 3D cube (2 × 2 × 2 cm^3) with UOP feature by self-healing. a, Cutting. **b**, Scratching. **c**, Folding. **d**, Binding. The dotted lines mean shallow scratches.

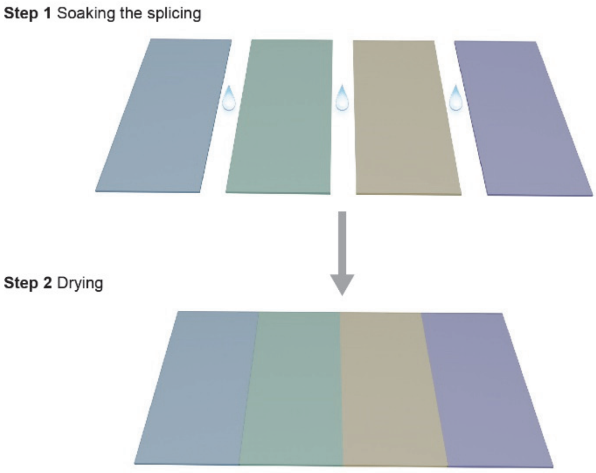

Supplementary Fig. 85 | Demonstration of ribbons fabricated by self-healing. There are two steps, which are soaking the splicing and drying, respectively.

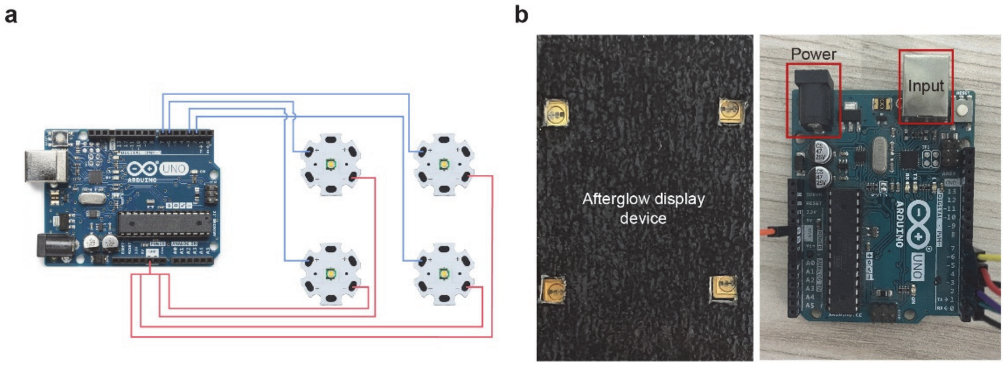

Supplementary Fig. 86 | Demonstration of multicolor afterglow display with electricity. **a**. Circuit diagram of afterglow display device. **b**. Photograph of afterglow display device.

Supplementary Table 16. Code of program for multicolor afterglow display.

```
   int a[4] = {2, 3, 5, 6};
int setTime[3] = {500, 1000, 5000};
void setup() {
   // put your setup code here, to run once:
   for (int i = 2; i < 8; i++)
   {
      pinMode(i, OUTPUT);
   }
}

void loop() {
   // put your main code here, to run repeatedly:
   {
      digitalWrite(a[i], HIGH);
      delay(200);
      digitalWrite(a[i], LOW);
      delay(200);
   }
}
```

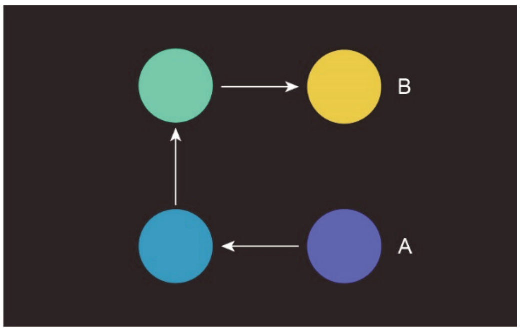

Supplementary Fig. 87. Electrical excitation sequence for afterglow devices with different UOP colors. From A to B, the sequences represent the CzA, FA, PXZA and NTIA doped films, respectively.

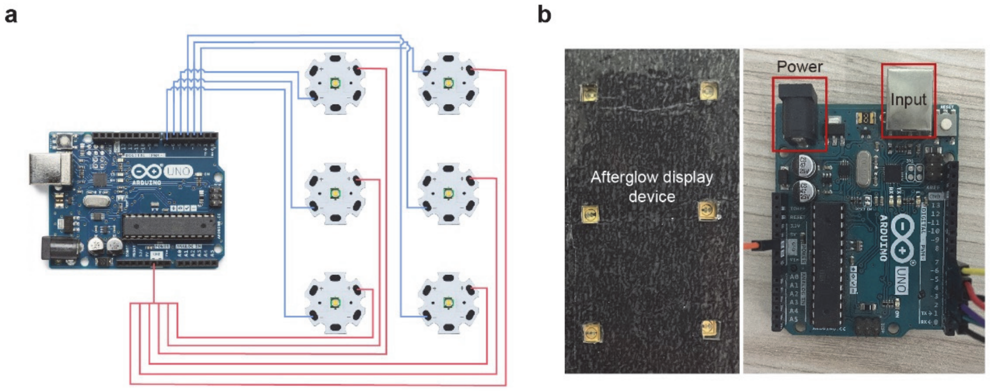

Supplementary Fig. 88 | Demonstration of afterglow display with electricity for path tracing and indicators. **a**, Circuit diagram of afterglow display device. **b**, Photograph of afterglow display device.

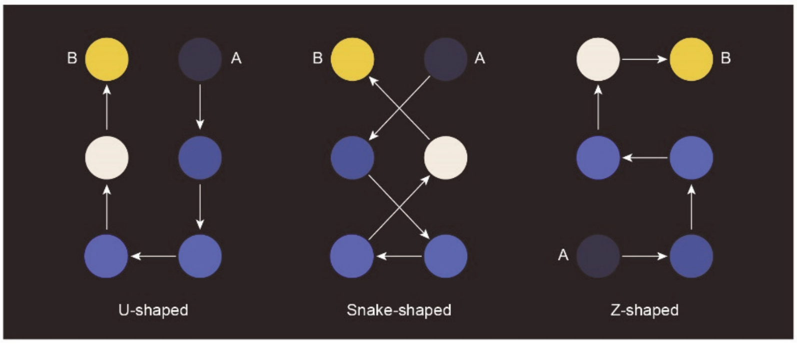

Supplementary Fig. 89 | Schematic diagram of path tracing. From A to B, it represents U-shaped (left), snake-shaped (middle) and Z-shaped (right) path tracing.

Supplementary Table 17. Code of program for U-shaped path track display.

```
int a[6] = {2, 3, 4, 7, 6, 5};
int setTime[3] = {500, 1000, 5000};
void setup() {
  // put your setup code here, to run once:
  for (int i = 2; i < 8; i++)
  {
    pinMode(i, OUTPUT);
  }
}

void loop() {
  // put your main code here, to run repeatedly:
  for (int t = 0; t < 3; t++)
  {
    for (int j = 0; j < 5; j++)
    {
      for (int i = 0; i < 6; i++)
      {
        digitalWrite(a[i], HIGH);
      }
      delay(200);
      for (int i = 0; i < 6; i++)
      {
        digitalWrite(a[i], LOW);
      }
      delay(setTime[t]);
    }
  }
```

Supplementary Table 18. Code of program for snake-shaped path track display.

```
int a[6] = {2, 6, 4, 7, 3, 5};
int setTime[3] = {500, 1000, 5000};
void setup() {
  // put your setup code here, to run once:
  for (int i = 2; i < 8; i++)
  {
    pinMode(i, OUTPUT);
  }
}

void loop() {
  // put your main code here, to run repeatedly:
  for (int t = 0; t < 3; t++)
  {
    for (int j = 0; j < 5; j++)
    {
      for (int i = 0; i < 6; i++)
      {
        digitalWrite(a[i], HIGH);
      }
      delay(200);
      for (int i = 0; i < 6; i++)
      {
        digitalWrite(a[i], LOW);
      }
      delay(setTime[t]);
    }
  }
```

Supplementary Table 19. Code of program for Z-shaped path track display.

```
int a[6] = {7, 4, 3, 6, 5, 2};
int setTime[3] = {500, 1000, 5000};
void setup() {
  // put your setup code here, to run once:
  for (int i = 2; i < 8; i++)
  {
    pinMode(i, OUTPUT);
  }
}

void loop() {
  // put your main code here, to run repeatedly:
  for (int t = 0; t < 3; t++)
  {
    for (int j = 0; j < 5; j++)
    {
      for (int i = 0; i < 6; i++)
      {
        digitalWrite(a[i], HIGH);
      }
      delay(200);
      for (int i = 0; i < 6; i++)
      {
        digitalWrite(a[i], LOW);
      }
      delay(setTime[t]);
    }
  }
```

Supplementary Table 20. Code of program for indicators.

```
int a[6] = {2, 3, 4, 5, 6, 7};
int setTime[3] = {500, 1000, 5000};
void setup() {
  // put your setup code here, to run once:
  for (int i = 2; i < 8; i++)
  {
    pinMode(i, OUTPUT);
  }
}

void loop() {
  // put your main code here, to run repeatedly:
for (int i = 0; i < 6; i++)
  {
    digitalWrite(a[i], HIGH);
  }
  delay(500);
  for (int i = 0; i < 6; i++)
  {
    digitalWrite(a[i], LOW);
  }
  delay(5000);
}
```

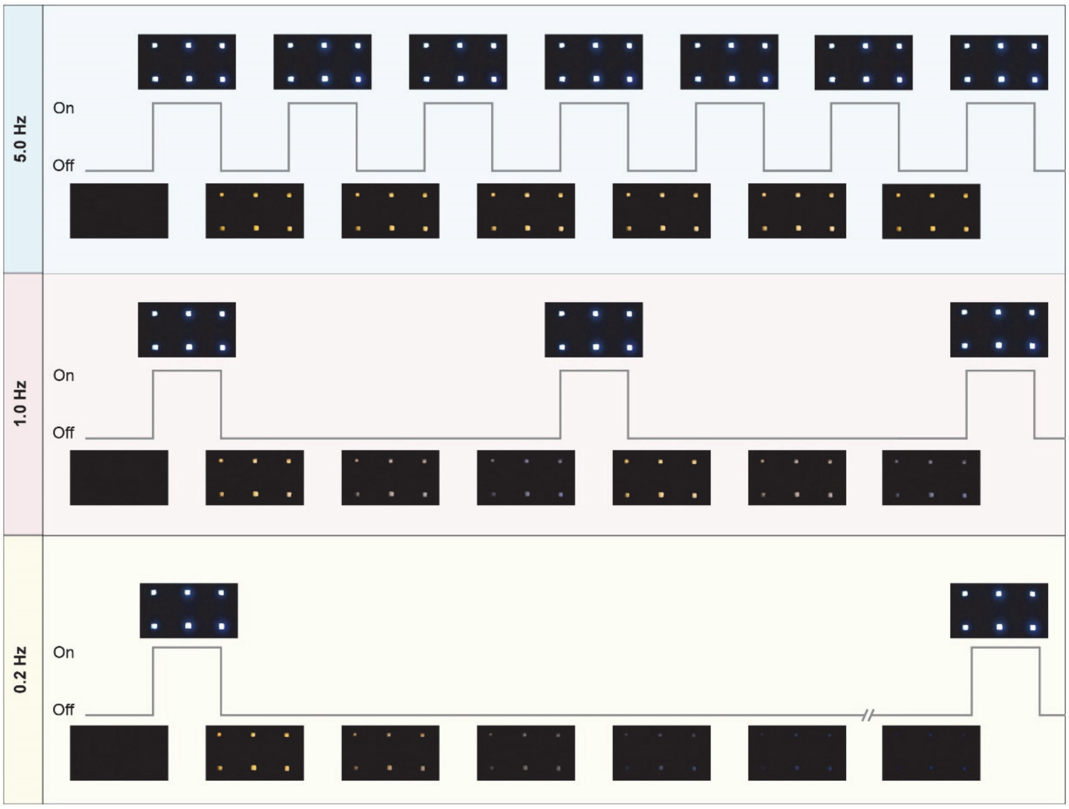

Supplementary Fig. 90 | Photography of indicators by reversible cycle of afterglow colors. The photographs were taken under different electrical excitation with 0.5 Hz (top), 1.0 Hz (middle) and 0.2 Hz (bottom).

VII. Supplementary Movies

The supplementary movies were recorded by a camera (Canon EOS 700D) under dark environment, including six supplementary movies from Movie 1 to Movie 6.

VIII. References

1. Chestnut, D. B., Wright, D. W. & Krizek, B. A. NMR chemical shifts and intramolecular Van der Waals interactions: carbonyl and ether systems. *J. Mol. Struct.* **190**, 99-111 (1988).

2. https://www.structbio.pitt.edu/images/sbl2014/notes/nmr_ref_notes_2011.pdf

3. Zarycz, M. N. C. & Fonseca Guerra, C. NMR ^{1}H-Shielding constants of hydrogen-bond donor reflect manifestation of the Pauli principle. *J. Phys. Chem. Lett.* **9**, 3720-3724 (2018).

4. Chen, X. *et al.* Versatile room-temperature-phosphorescent materials prepared from N-substituted naphthalimides: emission enhancement and chemical conjugation. *Angew. Chem. Int. Ed.* **128**, 10026-10030 (2016).

5. Wu, H. *et al.* Achieving amorphous ultralong room temperature phosphorescence by coassembling planar small organic molecules with polyvinyl alcohol. *Adv. Funct. Mater.* **29**, 1807243 (2019).

6. Louis, M. *et al.* Blue-light-absorbing thin films showing ultralong room-temperature phosphorescence. *Adv. Mater.* **31**, e1807887 (2019).

7. Thomas, H. *et al.* Aromatic phosphonates: a novel group of emitters showing blue ultralong room temperature phosphorescence. *Adv. Mater.* **32**, 2000880 (2020).

8. Lin, Z., Kabe, R., Nishimura, N., Jinnai, K. & Adachi, C. Organic long-persistent luminescence from a flexible and transparent doped polymer. *Adv. Mater.* **30**, 1803713 (2018).

9. Su, Y. *et al.* Excitation-dependent long-life luminescent polymeric systems under ambient conditions. *Angew. Chem. Int. Ed.* **59**, 9967-9971 (2020).

10. Kwon, M. S., Lee, D., Seo, S., Jung, J. & Kim, J. Tailoring intermolecular interactions for efficient room-temperature phosphorescence from purely organic materials in amorphous polymer matrices. *Angew. Chem. Int. Ed.* **53**, 11177-11181 (2014).

11. Salas Redondo, C. *et al.* Interplay of fluorescence and phosphorescence in organic biluminescent emitters. *J. Phys. Chem. C.* **121**, 14946-14953 (2017).

12. Lee, D. *et al.* Room temperature phosphorescence of metal-free organic materials in amorphous polymer matrices. *J. Am. Chem. Soc.* **135**, 6325-6329 (2013).

13. Gmelch, M., Thomas, H., Fries, F. & Reineke, S. Programmable transparent organic luminescent tags. *Sci. Adv.* **5**, u7310 (2019).

14. Su, Y. *et al.* Ultralong room temperature phosphorescence from amorphous organic materials toward confidential information encryption and decryption. *Sci. Adv.* **4**, s9732 (2018).
