# Supplementary material for: Ultralong organic phosphorescence from isolated molecules with repulsive interactions for multifunctional applications
Source: Nat Commun. 2022 Aug 19;13:4890. doi: 10.1038/s41467-022-32029-1 (PMC9391375; doi:10.1038/s41467-022-32029-1)
Supplement: Supplementary file 2 — Description of Additional Supplementary Files [file 41467_2022_32029_MOESM2_ESM.pdf]

Description of Additional Supplementary Files

File Name: Supplementary Movie 1
Description: Under excitation by a 302 nm UV-lamp, CzA/PVA, FA/PVA, PXZA/PVA, and NTIA/PVA films show blue, sky blue, blue-green and blue emission, respectively. After switching off the UV-lamp, we observed blue, sky blue, green, and yellow afterglow for several seconds by naked eyes. For PXZA/PVA and NTIA/PVA films, the afterglow emission disappears soon. When excited with a 365 nm UV-lamp, the afterglow was enhanced for PXZA/PVA and NTIA/PVA films. However, the afterglow disappears quickly for FA/PVA.

File Name: Supplementary Movie 2
Description: When excited with a 302 nm UV-lamp, the flexible Chinese paper-cutting emits a blue color. After switch-off of the UV-lamp, blue afterglow emission was clearly observed by the naked eye for several seconds.

File Name: Supplementary Movie 3
Description: This video demonstrates that 3D cube can emit visible blue UOP for dozens of seconds after excitation by a 302 nm lamp.

File Name: Supplementary Movie 4
Description: The video demonstrates multicolor afterglow display upon electrical power. After stoppage of the electrical power, blue, sky blue, green and yellow afterglow can be capture by the naked eye, respectively.

File Name: Supplementary Movie 5
Description: The afterglow display was used for path tracking. After ceasing DC power, tracks displayed with colorful afterglow from blue to white and then to yellow were clearly captured by the naked eye.

File Name: Supplementary Movie 6
Description: With frequency of electrical power change from 5.0, 1.0 to 0.2 Hz, the integrated device showed reversible cycle of colorful afterglow. The afterglow can show yellow (5.0 Hz), yellow to white (1.0 Hz), as well as yellow to white and then to blue (0.2 Hz).
